# Structure-Based Virtual Screening and Molecular Dynamics Simulation Assessments of Depsidones as Possible Selective Cannabinoid Receptor Type 2 Agonists

**DOI:** 10.3390/molecules28041761

**Published:** 2023-02-13

**Authors:** Gamal A. Mohamed, Abdelsattar M. Omar, Dana F. AlKharboush, Mona A. Fallatah, Ikhlas A. Sindi, Dina S. El-Agamy, Sabrin R. M. Ibrahim

**Affiliations:** 1 Department of Natural Products and Alternative Medicine, Faculty of Pharmacy, King Abdulaziz University, Jeddah 21589, Saudi Arabia; 2 Department of Pharmaceutical Chemistry, Faculty of Pharmacy, King Abdulaziz University, Jeddah 21589, Saudi Arabia; 3 Center for Artificial Intelligence in Precision Medicines, King Abdulaziz University, Jeddah 21589, Saudi Arabia; 4 King Abdulaziz Medical City, Jeddah 21423, Saudi Arabia; 5 Department of Biology, Faculty of Science, King Abdulaziz University, Jeddah 21589, Saudi Arabia; 6 Department of Pharmacology and Toxicology, Faculty of Pharmacy, Mansoura University, Mansoura 35516, Egypt; 7 Department of Chemistry, Preparatory Year Program, Batterjee Medical College, Jeddah 21442, Saudi Arabia; 8 Department of Pharmacognosy, Faculty of Pharmacy, Assiut University, Assiut 71526, Egypt

**Keywords:** depsidones, cannabinoid receptors, CB2 agonist, sustainable development goals, molecular docking, molecular dynamics, drug discovery

## Abstract

The discovery of natural drug metabolites is a leading contributor to fulfilling the sustainable development goal of finding solutions to global health challenges. Depsidones are a class of polyketides that have been separated from lichens, fungi, sponges, and plants and possess various bioactivities, including cytotoxic, antimicrobial, antimalarial, antituberculosis, acetylcholinesterase and α-glucosidase inhibition, and anti-inflammatory effects. Endocannabinoid receptors (CB1 and CB2) are G-protein-coupled receptors (GPCRs), and their activation mediates many physiological processes. CB1 is the dominant subtype in the central nervous system, while CB2 is mainly expressed in the immune system. The two receptors exhibit high heterogeneity, making developing selective ligands a great challenge. Attempts to develop CB2 selective agonists for treating inflammatory diseases and neuropathic pain have not been successful due to the high homology of the binding sites of the CB receptors. In this work, 235 depsidones from various sources were investigated for the possibility of identifying CB2-selective agonists by performing multiple docking studies, including induced fit docking and Prime/molecular mechanics–generalized Born surface area (MM-GBSA) calculations to predict the binding mode and free energy. Simplicildone J (**10**), lobaric acid (**110**), mollicellin Q (**101**), garcinisidone E (**215**), mollicellin P (**100**), paucinervin Q (**149**), and boremexin C (**161**) had the highest binding scores (−12.134 kcal/mol, −11.944 kcal/mol, −11.479 kcal/mol, −11.394 kcal/mol, −11.322 kcal/mol, −11.305 kcal/mol, and −11.254 kcal/mol, respectively) when screened against the CB2 receptor (PDB ID: 6KPF). The molecular dynamic simulation was performed on the compounds with the highest binding scores. The computational outcomes show that garcinisidone E (**215**) and paucinervin Q (**149**) could be substantial candidates for CB2 receptor activation and warrant further in vivo and in vitro investigations.

## 1. Introduction

Increasing drug discovery rates requires a research shift to advanced strategies for drug development. Natural products represent the basis for treating various human health disorders and will continue to be one of the fundamental areas of outlook therapeutics and medicine [1]. Currently, interest in natural products has been renewed because of the failure to discover safe and substantial lead agents with critical therapeutic properties, such as anti-infection, immunosuppression, and cancer and metabolic disorder prevention [2]. Several new tools, such as combinatorial chemistry, high-throughput screening, and automated separation techniques have been revolutionized to improve and accelerate drug discovery [3]. The profiteering of structurally diversified natural metabolite databases consisting of a wide variety of chemical classes, in combination with databases on proteins and target genes, will undoubtedly expedite the generation of new chemical entities via molecular computational modeling for bio-evaluation [4]. Therefore, the discovery of natural drug metabolites is a leading contributor to fulfilling the sustainable development goal of finding solutions to global health challenges.

Cannabinoid receptors (CB1 and CB2) are GPCRs (G protein-coupled receptors), which are renowned and have substantial roles in various human pathological and physiological conditions [5]. CB1R is fundamentally expressed in the CNS, while CB2R is principally expressed in peripheral tissues, regulating the immunological process, cytokine release, and cell migration [6]. CB2Rs are also found in different kinds of immuno-competent cells and inflammatory cells. It has been reported that an antinociceptive response is produced at the sites of neuropathic pain and inflammatory hyperalgesia due to peripheral CB2R activation [6,7,8]. Generally, activating CB1Rs is linked to unwanted consequences in the CNS, such as catalepsy and ataxia, while CB2R-selective agonists remarkably treat the pain without producing unwanted effects. Some of the developed CB1 receptor modulators possess serious side effects, for example, taranabant (MK-0364) and rimonabant (SR141716) are inverse CB1R agonists that progress as anti-obesity agents, causing CNS deleterious effects, such as suicidal ideation, anxiety, and depression [7], and consequently, they were withdrawn from clinical trials and the market. CB2 receptors have a notable role in neurological processes, including neuroinflammation and nociception [8].

Notably, some selectively developed CB2R agonists demonstrated considerable effectiveness in animal models and in vitro assays without possessing untoward psychoactive influences (e.g., GW-405833, JWH-015, and HU-308) [5]. Selective CB2R agonists are the main focus in the field of therapeutic uses because their modulation is a substantial approach to avoiding CNS-related untoward effects and treating inflammation, pain, arthritis, neuroprotection, addictions, and cancer [5]. Thus, it is clear that the discovery of CB2R-selective targets in natural products is a prominent area for treating a number of illnesses.

**Figure 1:**
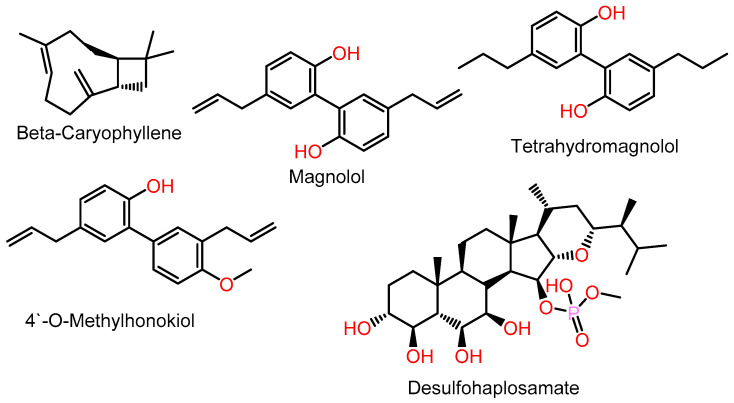
Examples of selective CB2R agonists.

Various studies have been carried out to evaluate the CB2R-modulating capacity of different natural metabolites (Figure 1). Trans β-caryophyllene, a CB2R agonist, displayed anti-inflammation potential mediated by CB2R and was found to have anti-cancer and anti-proliferation effects versus multiple myeloma (MM) cell lines, which could be utilized as antimyeloma therapy [9]. The main constituent of *Magnolia officinalis*, magnolol, and its metabolite tetrahydromagnolol, and some synthetic analogs revealed CB2R agonistic potential [10], whereas the minor constituent, 4′-O-methylhonokiol, was an inverse CB2 receptor agonist [11]. Chianese et al. stated that desulfohaplosamate had marked selectivity for CB2R at a low micromolar range [12].

Lichens are composite symbiotic organisms that consist of a fungus with one or more photosynthetic partners (e.g., cyanobacterium, green alga, or both) [13]. Lichens are encountered in various ecosystems, including drought, icebergs, hot deserts, rocky coasts, toxic heaps, continuous light, and prolonged darkness [14]. They can produce varied structures and unique metabolites, such as polyketides, and in particular, depsones, depsidones, dibenzofurans, depsides, and chromones [14,15].

**Figure 2:**
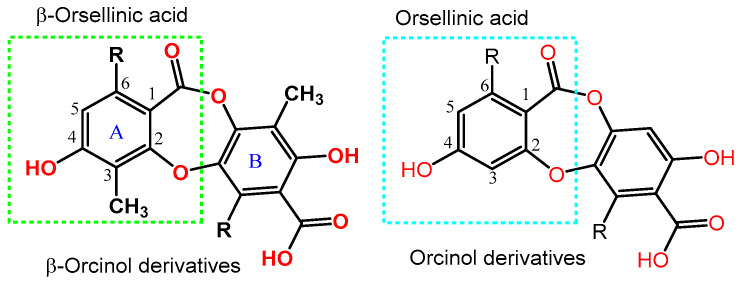
Basic skeleton of depsidones.

Among these metabolites, depsidones are phenolic polyketides predominantly biosynthesized by lichen, as well as by fungi and higher plants. Depsidones are cyclic ethers of polyphenolic depsides, with two 2,4-dihydroxy benzoic acid units connected by both ester and ether linkages to produce a rigid 11H-dibenzo[b,e][1,4]dioxepin-11-one core [16]. They are categorized as orcinol or ß-orcinol derivatives, relying on the C-3′s CH_3_ group on the rings (Figure 2) [17].

Depsidones have demonstrated diverse bioactivities, including anti-proliferative, antioxidant, antimalarial, antibacterial, cytotoxic, antihypertensive, anti-inflammatory, antifungal, and aromatase, protein tyrosine phosphatase, protein kinase, and HIV-1 integrase inhibitory properties [15,16,17].

In our continual interest to explore new bioactivities of the reported natural metabolites, 235 were depsidone derivatives, and 217 of them were reported to have naturally originated mainly from lichens (Figures S1–S9) inn addition, 18 nornidulin semisynthetic derivatives (Figure S10). They were investigated for their possible CB2R agonistic potential utilizing molecular docking and molecular dynamics simulations (Tables S1 and S2), with the hope that the findings could highlight new possible CB2R ligands that encourage further in vitro and in vivo research to prove this effect. Remarkably, no previous investigations have been published on the possible CB2R-modulating activity of this class of metabolites and its related derivatives.

The depsidone derivatives were screened by generating a grid from the CB2 receptor (PDB ID: 6KPF) and docking the compounds into the grid, generating different docking scores, including the XP GScore and induced fit docking score. In addition, MM-GBSA was used to calculate the binding free energy between the protein and the ligand. Compounds with the highest docking scores were then subjected to a molecular dynamic (MD) simulation.

## 2. Results and Discussion

### 2.1. Protein and Ligand Preparation

The low-energy ionization and tautomeric states of 3D structures were generated in 2D using LigPrep. The generated three-dimensional structures were docked into the CB2 receptor (PDB-ID: 6KPF) [18] prepared via the protein preparation wizard tool, where the H bonds were optimized, and the geometry minimized. Missing hydrogen atoms and the correct ionization states were added to confirm the correct formal charges and force field treatment assignment.

### 2.2. Molecular Docking Studies Analysis

**Figure 3:**
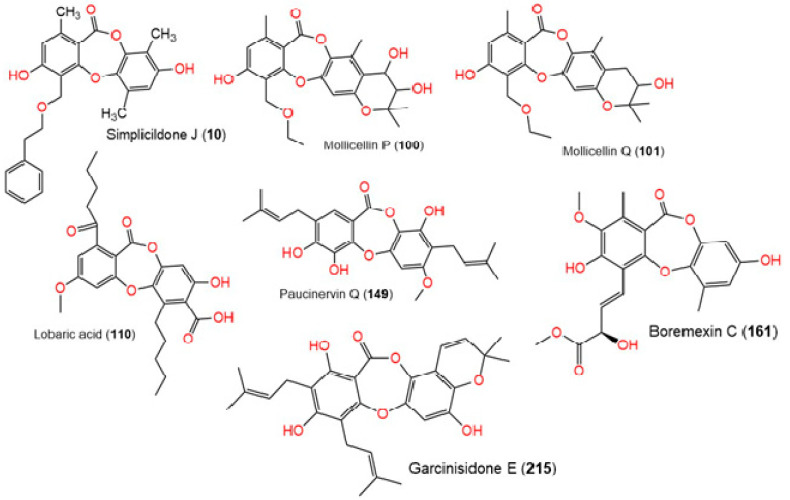
Chemical structure of depsidones with higher docking scores.

After preparing the protein and the ligands, the prepared 3D structures were docked into the selected grid box in the prepared protein (PDB-ID: 6KPF). The native agonist bound to the CB2 receptor (PDB-ID: 6KPF), 7-[(6aR,9R,10aR)-1-hydroxy-9-(hydroxymethyl)-6,6-dimethyl-6a,7,8,9,10,10a-hexahydro-6H-benzo[c]chromen-3-yl]-7-methyloctanenitrile (PDB-ID: **E3R**), and 235 depsidone ligands were prepared and docked into the co-crystallized binding site of the CB2 receptor. Table 1 outlines the docking scores based on the most negative XP scores. Different docking scores were calculated, such as the Glide Emodel, the GlideScore, and the XP GScore. Among the tested ligands, simplicildone J (**10**), lobaric acid (**110**), mollicellin Q (**101**), garcinisidone E (**215**), mollicellin P (**100**), paucinervin Q (**149**), and boremexin C (**161**) showed the best binding to the CB2 receptor according to the XP and Glide docking scores (−12.134 kcal/mol, −11.944 kcal/mol, −11.479 kcal/mol, −11.394 kcal/mol, −11.322 kcal/mol, −11.305 kcal/mol, and −11.254 kcal/mol, respectively) compared to the native agonist **E3R** (−12.240 kcal/mol) (Figure 3).

The binding interactions of these compounds with the protein were examined. To validate these findings, the native agonist **E3R** was redocked alongside the depsidone ligands, and the docking positions were examined.

**Figure 4:**
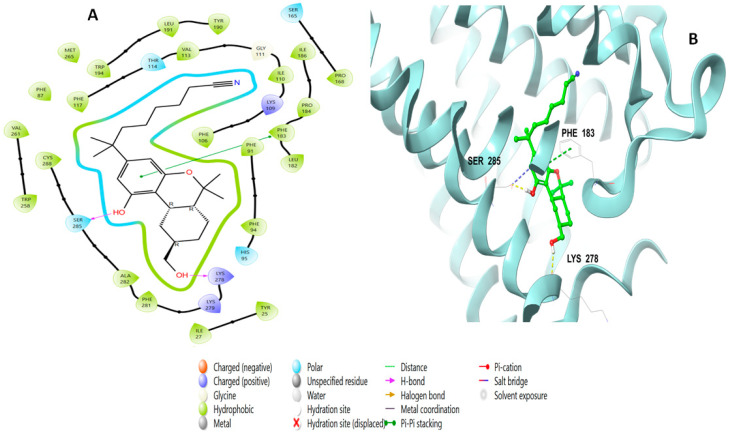
Putative binding mode of native agonist (PDB-ID: **E3R**) in the binding site of CB2 receptor (PDB-ID: 6KPF). **E3R** is displayed as green sticks. H bonds are displayed as yellow dotted lines and ionic bonds as blue dotted lines. (**A**) 2D depict; (**B**) 3D representation of CB2 complexed with **E3R**.

Figure 4 represents the binding interactions of the native agonist **E3R** with the CB2 receptor in 3D and 2D views.

**Figure 5:**
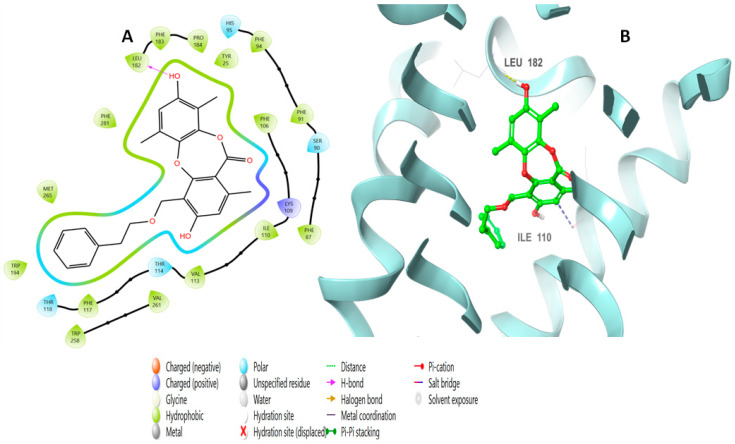
Putative binding mode of simplicildone J (**10**) at the binding site of CB2 receptor (PDB-ID: 6KPF). Simplicildone J (**10**) is displayed as green sticks. H bonds are displayed as yellow dotted lines, and ionic bonds as blue dotted lines. (**A**) Two-dimensional view; (**B**) three-dimensional view of CB2 complexed with simplicildone J (**10**).

The binding interactions consisted of a pi–pi stacking with Phe183 and hydrogen bonds with Ser285 at a distance of 1.76 Å and with Lys278 at a distance of 1.98 Å. Simplicildone J (**10**) had different interactions with the amino acid residues. Figure 5 shows the 2D and 3D views of these interactions, which included an H bond through its hydroxyl group with Leu182 at a distance of 1.94 Å, as well as ionic binding interactions with Ile110.

**Figure 6:**
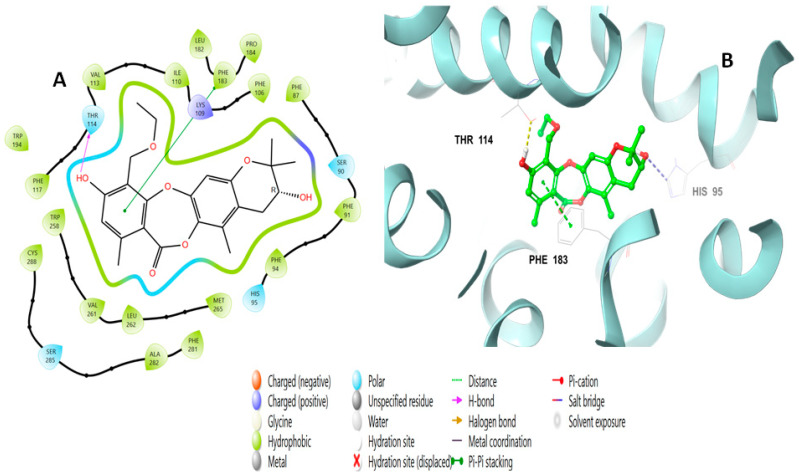
Putative binding mode of mollicellin Q (**101**) at the binding site of the CB2 receptor (PDB-ID: 6KPF). Mollicellin Q (**101**) is displayed as green sticks. H bonds are displayed as yellow dotted lines, and ionic bonds as blue dotted lines. (**A**) Two-dimensional view; (**B**) three-dimensional view of CB complexed with mollicellin Q (**101**).

The binding interactions of mollicellin Q (**101**) are shown in Figure 6, and included a pi–pi stacking at a distance of 4.92 Å with Phe183, a hydrogen bond with Thr114 at a distance of 2.15 Å, and an ionic bond with His95.

**Figure 7:**
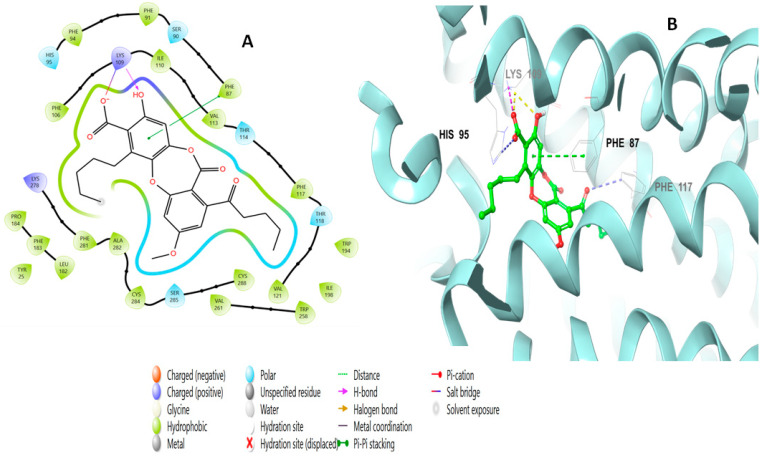
Putative binding mode of lobaric acid (**110**) at the binding site of CB2 receptor (PDB ID: 6KPF). Lobaric acid (**110**) is displayed as green sticks. H bonds are displayed as yellow dotted lines, and ionic bonds as blue dotted lines. (**A**) Two-dimensional view; (**B**) three-dimensional view of CB2 complexed with lobaric acid (**110**).

**Figure 8:**
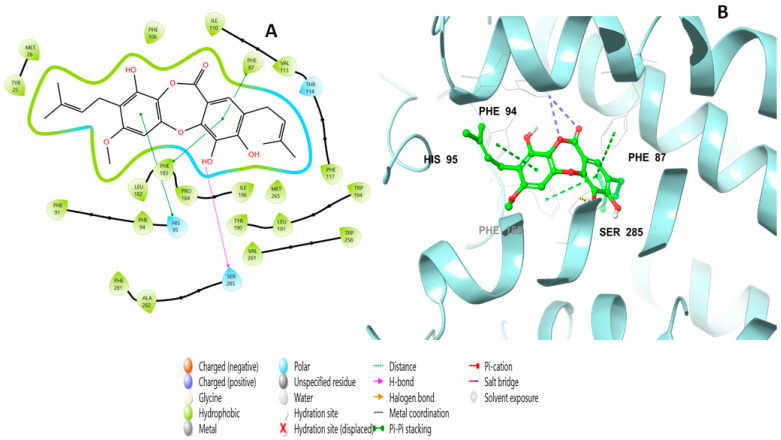
Putative binding mode of paucinervin Q (**149**) in the binding site of CB2 receptor (PDB-ID: 6KPF). Paucinervin Q (**149**) is displayed as green sticks. H bonds are displayed as yellow dotted lines, and ionic bonds as blue dotted lines. (**A**) Two-dimensional view; (**B**) three-dimensional view of CB2 complexed with paucinervin Q (**149**).

**Figure 9:**
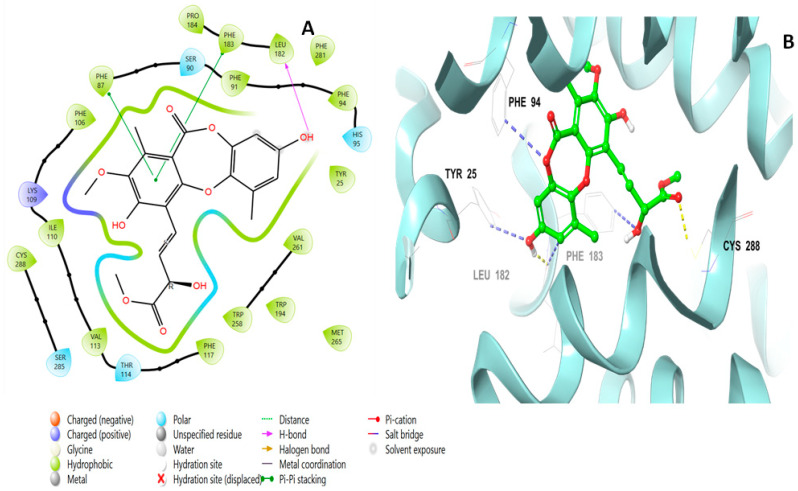
Putative binding mode of boremexin C (**161**) at the binding site of CB2 receptor (PDB-ID: 6KPF). Boremexin C (**161**) is displayed as green sticks. H bonds are displayed as yellow dotted lines, and ionic bonds as blue dotted lines. (**A**) Two-dimensional view; (**B**) three-dimensional view of CB2 complexed with boremexin C (**161**).

**Figure 10:**
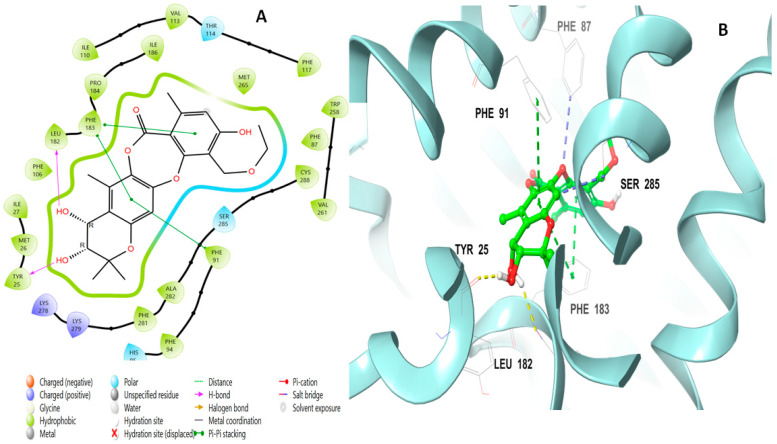
Putative binding mode of mollicellin P (**100**) at the binding site of CB2 receptor (PDB-ID: 6KPF). Mollicellin P (**100**) is displayed as green sticks. H bonds are displayed as yellow dotted lines, and ionic bonds as blue dotted lines. (**A**) Two-dimensional view; (**B**) three-dimensional view of CB2 complexed with mollicellin P (**100**).

Lobaric acid (**110**) also interacted through its aromatic ring, forming a pi–pi stacking interaction with Phe87 at a distance of 5.47 Å, as well as a hydrogen bond with Lys 109 through its hydroxyl group and two ionic bonds with His95 and Phe117 (Figure 7). Paucinervin Q (**149**) formed a hydrogen bond with Ser285; it also formed several pi–pi stacking interactions with Phe183, at a distance of 5.13 Å, and with Phe87, Phe94, and His95 (Figure 8). Figure 9 illustrates the binding interactions of boremexin C (**161**) with the residues, including a pi–pi stacking with Phe87, at a distance of 5.38 Å, and with Phe183 and a hydrogen bond with Leu182 at a distance of 1.78 Å. A pi–pi stacking interaction at a distance of 5.18 Å and with Phe183 at a distance of 5.10 Å, and hydrogen bonding interactions with Tyr25 at a distance of 1.67 Å and Leu182 with mollicellin P (**100**) are shown in Figure 10.

The Prime/MM–GBSA equation was used to calculate the binding free energies (DGbind) of each ligand based on the docking complex. The more negative values indicate a stronger binding (Table 1).

### 2.3. Induced Fit Docking Analysis

In reality, proteins undergo backbone or sidechain movements upon ligand binding, making it difficult to assume a rigid receptor and model the real binding process [19]. Induced fit binding is one of the most challenging factors in drug design. The IFD scores of the best binding ligands are listed in Table 2. According to the obtained IFD scores, all compounds show comparable results to the prepared ligand, which indicates good binding. Paucinervin Q (**149**), garcinisidone E (**215**), and lobaric acid (**110**) had better IFD scores than the prepared ligand, which is an indication of a better interaction with the CB2 receptor.

### 2.4. QM/MM (Quantum Mechanics/Molecular Mechanics) Analysis

After induced fit docking, the best position was chosen and introduced to the QM/MM computation. The QM method was used for the protein’s active site, while the rest of the proteins were treated by the MM method. This offers the advantage of obtaining accurate results by avoiding the demands of the computational work for calculating the QM for a large number of atoms [20]. The quantum mechanics method offers the advantage of accurately calculating the internal energies and HOMO/LUMO values [19]. The highest occupied molecular orbital (HOMO) and the lowest unoccupied molecular orbital (LUMO) are located at the outer boundaries of the molecule’s electrons. The ionization potential is related to the HOMO, while the electron affinity is linked to the LUMO [21]. The energy gap between the HOMO and LUMO in the native agonist was the lowest, indicating higher chemical reactivity [21]. As shown in Table 2, the QM/MM energy of the native agonist was higher than the tested depsidone derivatives, which indicates that they had a higher potential than the native agonist.

### 2.5. Molecular Dynamic Simulation

**Figure 11:**
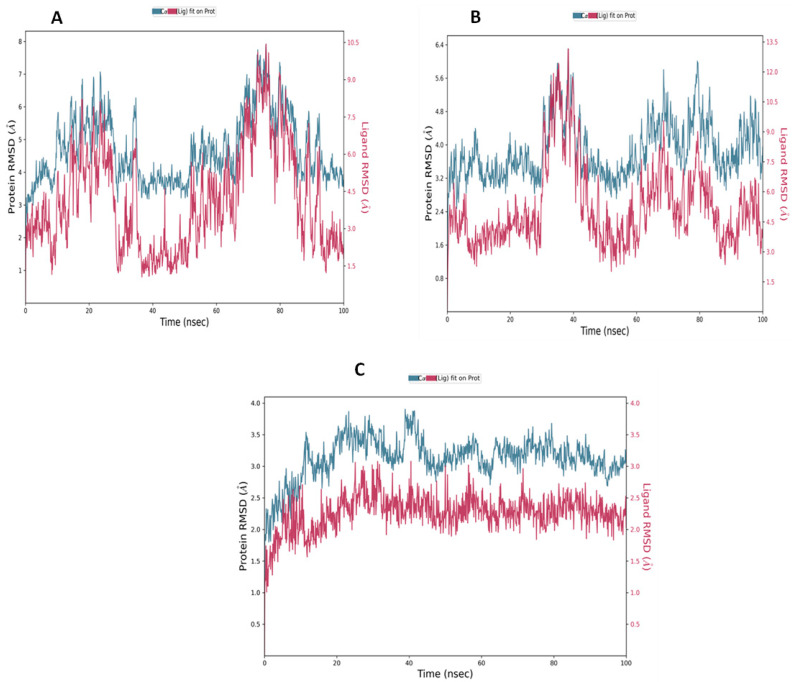
RMSD plot for (**A**) native agonist (PDB ID: E3R); (**B**) boremexin C (**161**); (**C**) garcinisidone E (**215**) with CB2 receptor (PDB ID: 6KPF) during the MD simulation.

Molecular dynamic simulations utilize Newtonian physics in order to study atomic motions and investigate the behavior of molecules in action [22]. A variety of biomolecular processes can be captured during a molecular dynamic simulation, including ligand binding, protein folding, and conformational changes [23]. The simulation is carried out under precisely controlled conditions. Molecular dynamic simulations were performed on the native agonist and the best binding agonists to validate the docking scores. The MD outcome of the native agonist was used as a control. The root mean square deviation (RMSD) may be utilized to study the conformational stability of a structure during the simulation by measuring the average change in the displacement of atoms concerning a reference [24]. The average change in the displacement of boremexin C (**161**) (Figure 11B) and garcinisidone E (**215**) (Figure 11C) concerning a reference agonist (PDB: E3R) (Figure 11A) was measured using the RMSD to predict the stability of the complex. The RMSD progression of the CB2 protein (left Y-axis) aligned on the reference frame backbone and the RMSD of the ligand (right Y-axis) are both shown in the plot. The RMSD plot of the native agonist (Figure 11A) reveals that there is high fluctuation, more than 1–3 Å, which indicates that the CB2 receptor and native agonist complex are unstable and there was large conformational change in the protein during the simulation. However, the RMSD plots of **161** (Figure 11B) and **215** (Figure 11C) show that both complexes were stable and the slight fluctuations that occurred at the time of the simulation were within the acceptable range (1–3 Å).

**Figure 12:**
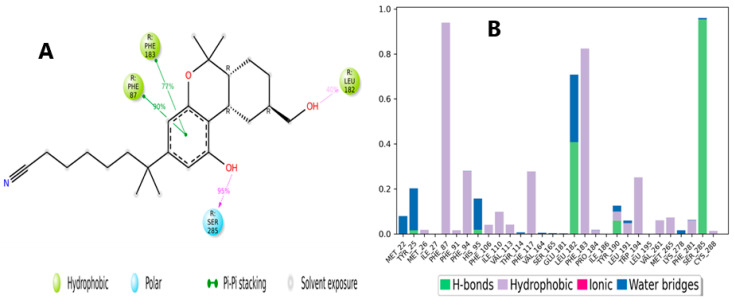
(**A**) Schematic detailed representation of native agonist (PDBID: **E3R**) interactions with the CB2 receptor (PDB ID: 6KPF) residues, where blue represents the polar residues and green represents the hydrophobic residues. (**B**) Normalized stacked bar chart representing the interaction of the CB2 binding site residues with the native agonist throughout the simulation: hydrophobic (violet), hydrogen bonds (green), and water bridges (blue). The stacked bar charts are normalized over the course of the trajectory.

Figure 12A represents a scheme of the binding interactions of the native agonist (PDB ID: **E3R**) and the amino acid residues of the CB2 receptor (PDB ID: 6KPF). Throughout the simulation, docked positions were maintained. The native agonist interacted through a hydrogen bond with Ser285, which was maintained for over 90% of the simulation time (100.00 ns), as well as a pi–pi stacking interaction with Phe87 (90%). Other interactions included a pi–pi stacking interaction with Phe183 (77%) and a hydrogen bond with Leu182 (40%). The interactions are also presented as stacked bars and categorized into three types: hydrogen bonds, hydrophobic interactions, and water bridges (Figure 12B). The major contacts between the ligand and the CB2 receptor included hydrophobic interactions with Phe87 and Phe183 for over 80% of the trajectory time. The protein–ligand interactions also included hydrogen bonding interactions with Ser285 and Leu182, which were maintained for over 90% of the simulation.

**Figure 13:**
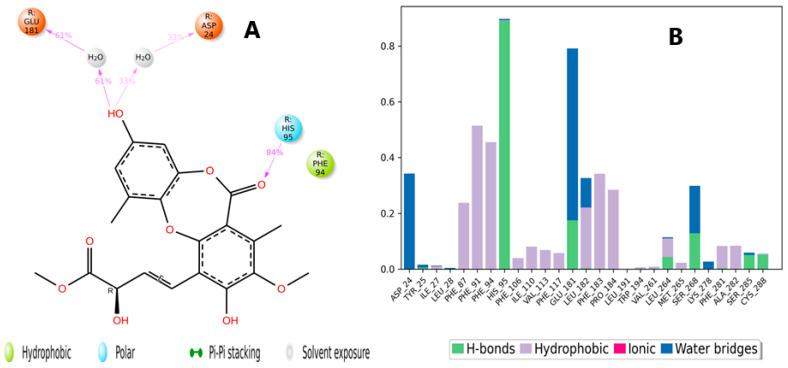
(**A**) Schematic detailed representation of boremexin C (**161**) interactions with the CB2 receptor (PDBID: 6KPF) residues, where blue represents the polar residues and green represents the hydrophobic residues. (**B**) Normalized stacked bar chart representing the interaction of the CB2 binding site residues with boremexin C (**161**) throughout the simulation: hydrophobic (violet), hydrogen boremexin (green), and water bridges (blue). The stacked bar charts are normalized over the course of the trajectory.

**Figure 14:**
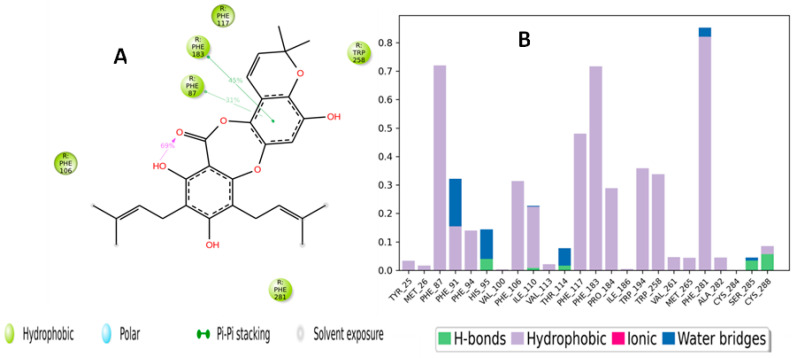
(**A**) Schematic detailed representation of garcinisidone E (**215**) interactions with the CB2 receptor (PDBID: 6KPF) residues, where blue represents the polar residues and green represents the hydrophobic residues. (**B**) Normalized stacked bar chart representing the interaction of the CB2 binding site residues with garcinisidone E (**215**) throughout the simulation: hydrophobic (violet), hydrogen bonds (green), and water bridges (blue). The stacked bar charts are normalized over the course of the trajectory.

The binding interactions of boremexin C (**161**) with the CB2 receptor are presented in Figure 13A and include a hydrogen bond between the carbonyl oxygen and His96, which lasted for 84% of the simulation time. In addition, the hydroxyl formed two water-bridged hydrogen bonds with Glu181 and Asp24, which lasted for 61% and 33% of the trajectory time, respectively. Figure 13B displays the residue contacts of **161** with the CB2 receptor in the form of stacked bars. As shown in Figure 13A, the hydrogen bond interaction with His95 was maintained for more than 80% of the trajectory time. Boremexin C (**161**) interacted through both a hydrogen bond and a water-bridged interaction with Glu18. Several hydrophobic interactions were formed with various residues, including Phe91, Phe94, and Phe183. Figure 14A is a detailed 2D schematic representation of garcinisidone E (**215**) interactions with CB2 receptor residues that occurred for more than 30.0% of the simulation time in the selected trajectory (0.00 through 100.00 nsec). Molecular interactions included two pi–pi stacking interactions with Phe183 and Phe87 for almost 45% and 31% of the simulation time, respectively. The scheme also shows an intramolecular H bond between the hydroxyl group and the carbonyl oxygen, which increased the rigidity of the compound and occurred for 69% of the simulation time. The contacts of boremexin C (**161**) and the CB2 receptor are illustrated as stacked bars, which are normalized over the course of the trajectory and were classified into three main types: hydrophobic, hydrogen bonds, and water-bridged interactions (Figure 14B). These contacts consisted mainly of hydrophobic interactions and a few hydrogen bonds. The hydrophobic interactions showed different degrees of maintenance throughout the simulation: Phe281 (80%), Phe183 (70%), and Phe117 (50%).

### 2.6. ADMET Properties

The ADMET properties, which include the absorption, distribution, metabolism, and excretion of the depsidone derivatives were examined using QikProp of the Schrodinger module and are listed in Table 3. This enables the prediction of many physiochemical properties and biological functions, which could help predict the usefulness of the drug and reduce failures throughout the discovery and development process. In addition to predicting the physiochemical properties of the compounds, other factors were also examined, including the molecular weight, metabolism, dipole moment, hydrogen bond donors and acceptors, CNS activity, human oral absorption, and the partition coefficient. It was found that most of the descriptors were within range except for a few, such as poor absorption and high metabolism.

## 3. Materials and Methods

### 3.1. Preparation of Protein

The CB2 receptor X-ray crystal structure (PDB ID:6KPF) [25] was downloaded from the protein data bank. Using the protein preparation wizard in Maestro Schrödinger [18], the protein was prepared by adding the missing hydrogens to the residues, correcting the metal ionization states, and removing water molecules beyond 5 Å. In addition, the correct charges were assigned, and the protein underwent restrained minimization using an OPLS4 force field.

### 3.2. Ligand Preparation

Schrödinger’s LigPrep tool [26] was used for docking 235 depsidone derivates, and the OPSL3 forefield was utilized for the energy-minimized 3-dimensional structures. Subsequently, Epik generated all the applicable ionization states and tautomeric forms at a pH of 7.0 ± 0.2 and added hydrogens. Additionally, PROPKA-optimized H bonds and any water molecules beyond 3 Å away from the HET groups were taken away.

### 3.3. Grid Generation and Molecular Docking

The ligand in the co-crystallized structure of 6KPF was selected to define the grid box, and the binding site was defined using Glide’s Receptor–Grid Generation tool [27]. The grid box had a length of 10 Å in each of the X, Y, and Z dimensions. The assigned grid box was used for docking the prepared ligands using the Ligand Docking Tool in the Schrödinger suite. The chosen protocol was the extra-precision (XP) protocol. The partial charge cut-off was 0.25, and the VdW radii scaling factor was set to 1.0. The co-crystallized ligand was redocked alongside depsidone derivatives to validate the docking method. The other settings were kept at their default. The G score (ranks the compounds), Emodel (ranks the conformers), and XP GScore were calculated to assess the docking scores. In order to approximate the ligand binding free energy and rank the positions of different ligands, the GlideScore was calculated. Glide uses Emodel to select the best position of the ligand and then uses the GlideScore to rank the best positions against each other. The XP Glide was used to rank the ability of the ligands to bind to a specific conformation of the protein receptor and is presented by the following equation:

XP GlideScore = E*_coul_* + E*_VdW_* + E*_bind_* + E*_penalty_*

Ebind = E*_hyd_enclosure_* + E*_hb_nn_motif_* + E*_hb_cc_motif_* + E*_PI_* + E*_hb_pair_* + E*_phobic_pair_*

Epenalty = E*_desolv_* + E*_ligand_strain_*

where E is the energy (calculated for each of the following descriptors); E*_coul_* is the Coulomb energy, E*_VdW_* is the van der Waal force, E*_bind_* is the energy that favors binding, E*_penalty_* is the penalty that disfavors binding, E*_hyd_enclosure_* is the hydrophobic enclosure, E*_hb_nn_motif_* is a special neutral–neutral hydrogen bond motif, E*_hb_cc_motif_* is a special charged–charged hydrogen bond motif, E*_PI_* is a pi–cation interaction, E*_hb_pair_* is a hydrogen bond pair, E*_phobic_pair_* is a lipophilic pair, and E*_desolv_* is the desolvation energy [28].

Prime was used to calculate the molecular mechanics–generalized born surface area (MM-GBSA) [27] for re-scoring the docked positions. The binding free energy was calculated using the following equation:

ΔGbind = E_Complex (minimized) − E_ligand (minimized) − E_protein (minimized)

where (Complex) is the protein–ligand complex, (Eprotein) is the free protein, and (Eligand) is the free ligands.

### 3.4. Induced Fit Docking

Induced fit docking (IFD) protocol was performed in order to efficiently model the flexibility of the receptor and the ligand using the induced fit docking function in Maestro v9.1. (Schrodinger, LLC) [29,30]. The complex was used to generate the centroid of the residues by picking up the ligand from the protein. The ligands were docked into the protein using Glide. The sidechains were trimmed automatically based on their B-factor, with receptor and ligand van der Waals forces of 0.50 each. Twenty positions were generated and used to test the protein’s plasticity. Residues were refined within 5.0 Å of the ligand positions and subjected to prime sidechain prediction and energy minimization, and then the sidechains were optimized. Ligand structures and conformations were induced to fit each position of the receptor. Then, redocking using Glide SP was performed on the best structures. Lastly, the ligand was docked into the induced fit receptor, and an IFD score for each position was generated.

### 3.5. Molecular Dynamic Simulation (MD)

Desmond software in the Schrödinger package [31] was used to run the molecular dynamic simulation of the compounds with the highest docking scores. A solvated system was prepared by immersing the protein–ligand complex into an orthorhombic water box, which was set to extend 10 Å beyond the atoms in the complex. The system was neutralized by adding Na and Cl counter ions. The simulation time was set at 100 ns, and the simulation was performed at a constant temperature of 300 K and constant pressure of 1.01325 bar.

### 3.6. Quantum Mechanics/Molecular Mechanics (QM/MM) Calculations

The geometries used for performing the QM/MM calculations were obtained from the induced fit docking protocol detailed above. The QM/MM calculations were run on the protein (PDB-ID: 6KPF), and the native agonist (PDB-ID: **E3R**) and the depsidone derivatives had the highest docking scores. The Q site program [32] was used to calculate the QM/MM by including the ligands and the residues in the interaction. The QM region was created by picking up the free ligands and sidechains. The DFT-B3LP method, which is a measure of electron density, was chosen to perform the QM calculations, and the QM system had a charge of −1. The OPLS-2005 force field was selected to treat the MM region, which consisted of the rest of the system. After the QM/MM run was completed, the highest occupied molecular orbital (HOMO), lowest unoccupied molecular orbital LUMO, and energy gaps were calculated.

### 3.7. Prediction of ADMET Properties

Predicting the ADMET properties of the compounds, which are absorption, distribution, metabolism, and elimination, provided some vital information related to the drugs/molecules. All compounds were assessed using the Maestro Schrodinger QikProp module to identify promising molecules according to their bioavailability properties and ADMET profiling [33].

## 4. Conclusions

Natural metabolites originating from various sources, e.g., animals, plants, and microorganisms display high structural complexity and diversity. Taking into consideration that a high-throughput in vivo/in vitro investigation for assessing the bioactivities of natural metabolites is costly and time-consuming, quite effective in silico tools could assist as rapid, auspicious, and cost-efficient strategies to predict the natural metabolites–target association before experimental validity. CB2R is a possible therapeutic target for treating various illnesses, such as neurodegenerative diseases (e.g., Huntington’s, Alzheimer’s, and Parkinson’s diseases and multiple and amyotrophic lateral sclerosis), as well as inflammation, pain, myocardial infarction, irritable bowel syndrome, and various cancer types. For the identification of potential CB2R agonists, 235 depsidone derivatives were virtually screened for their CB2R-binding potential using multiple virtual studies: extra precision docking, induced fit docking, and Prime/MM-GBSA calculation, as well as MD simulations. These methods proved the CB2R agonistic activity of simplicildone J (**10**), lobaric acid (**110**), mollicellin Q (**101**), garcinisidone E (**215**), mollicellin P (**100**), paucinervin Q (**149**), and boremexin C (**161**) and stability during the MD simulation studies. The variation in the binding affinity of the compounds based on the methods of calculation—for example, induced fit docking and QM/MM—was attributed to the difference in parameters and conditions used for each calculation; however, there were no significant differences amongthe results. The findings of this work reveal that **149** and **215** demonstrated the highest binding affinity to the CB2R. Therefore, they could be lead candidates for CB2 receptor activation, which warrants further in vivo and in vitro investigations.

## Figures and Tables

**Table 1:** Docking results of in silico screening against CB2 receptor (PDB-ID:6KPF) and binding free energy.

Title	Docking Score	XP GScore	GlideScore	Glide Emodel	Prime Energy	MMGBSA dG Bind	IFD Score
6KPF–prepared _ligand	−12.240	−12.240	−12.240	−79.066	−43,026.5	−86.26	−532.50
Simplicildone J (**10**)	−12.134	−12.174	−12.174	−17.822	−43,002.1	−52.76	−530.54
Lobaric acid (**110**)	−11.944	−11.944	−11.944	−37.511	−42,921.3	−20.56	−533.43
Mollicellin Q (**101**)	−11.479	−11.513	−11.513	55.427	−42,995.8	−56.65	−531.30
Garcinisidone E (**215**)	−11.394	−11.633	−11.633	15.908	−43,035.0	−54.14	−534.60
Mollicellin P (**100**)	−11.322	−11.356	−11.356	−16.759	−42,996.9	−65.65	−531.21
Paucinervin Q (**149**)	−11.305	−11.542	−11.542	−23.918	−42,958.9	−66.13	−536.89
Boremexin C (**161**)	−11.254	−11.376	−11.376	−12.854	−42,950.3	−61.63	−531.22

XP: extra precision; MMGBSA: molecular mechanics with generalized Born and surface area solvation; IFD: induced fit docking.

**Table 2:** HOMO, LUMO, energy gap, and QM/MM energies (kcal/mol).

Compound	Number of Canonical Orbitals	QM/MM Energy	HOMO	LUMO	Energy Gap
Paucinervin Q (**149**)	1018	−2655.605583	−0.437907	−0.278268	0.716175
Garcinisidone_E (**215**)	848	−2278.252156	−0.428884	−0.263329	0.692213
Lobaric_acid (**110**)	980	−2599.618583	−0.442702	−0.261762	0.708782
6KPF_Native Agonist	759	−1865.074602	−0.418376	−0.216867	0.635243

QM/MM: quantum mechanism/molecular mechanics; HUMO: highest occupied molecular orbital; LUMO: lowest unoccupied molecular orbital.

**Table 3:** In silico ADME properties of tested depsidone derivatives.

Molecule	#stars	#rtvFG	CNS	mol_MW	SASA	donorHB	accptHB	QPlogPo/w	QPlogHERG	QPPCaco	QPlogBB	#metab	QPlogKhsa	Percent Human Oral Absorption
**Recommended Range**	(0.0–5.0)	(0–2)	(−2 inactive) (+2 active)	(130–725)	(300–1000)	(0–6)	(2.0–20.0)	(−2–6.5)	concen below −5	<25 poor, >500 great)	(−3–1.2)	(1–8)	(−1.5–1.5)	(<25% poor; >80% high)
Boremexin C (**161**)	0	2	−2	416.384	650.358	3	8.95	1.511	−4.765	85.053	−1.935	6	−0.131	70.328
Garcinisidone E (**215**)	2	1	−2	478.541	778.681	2	5	5.471	−5.428	603.731	−1.194	9	1.25	95.791
Lobaric acid (**110**)	0	1	−2	456.491	782.184	1	7.5	4.226	−3.811	28.355	−2.352	4	0.325	77.691
Mollicellin P (**100**)	0	1	−2	430.454	702.313	3	9.6	2.213	−5.061	403.047	−1.284	7	−0.021	86.531
Mollicellin Q (**101**)	0	1	−2	414.454	698.882	2	7.9	3.048	−5.079	657.833	−1.004	7	0.283	95.231
Paucinervin Q (**149**)	1	1	−2	426.465	733.431	3	6	3.82	−5.24	307.837	−1.578	10	0.607	93.85
Simplicildone J (**10**)	0	1	−2	420.461	708.462	2	6.2	4.133	−6.007	660.471	−1.12	8	0.578	100

Number of stars: property or descriptor values that fall outside the 95% range of similar values for known drugs. More stars indicate that a molecule is less drug-like than molecules with fewer stars; SASA: total solvent-accessible surface area in square angstroms utilizing a probe with a 1.4 Å radius; dipole: computed dipole moment of the molecule; acceptor H bond: estimated number of hydrogen bonds that the solute would accept from water molecules in an aqueous solution; donor H bond: H bonds estimated number that the solute would donate to H2O molecules in an aqueous solution; QPlogS: predicted aqueous solubility, log S; QPlogPo/w: predicted octanol/water partition coefficient; QPlogkhsa: prediction of binding to human serum albumin; QplogBB: predicted brain/blood partition coefficient; No. of metabolites: number of likely metabolic reactions; % Human Oral Absorption: predicted human oral absorption on 0 to 100% scale; CNS: predicted central nervous system activity on a −2 (inactive) to +2 (active) scale; QPlogHERG: predicted IC50 value for blockage of HERG K+ channels; #rtvFG: reactive functional groups number; the specific groups are listed in the jobname.out file. The presence of these groups can lead to false positives in HTS assays and to reactivity, decomposition, or toxicity problems in vivo.

## Data Availability

The data presented in this study are available in the article.

## Supplementary Materials

The following supporting information can be downloaded at: https://www.mdpi.com/article/10.3390/molecules28041761/s1, Figures S1–S10: Chemical structures of all tested depsidone derivatives: title; Table S1: ADMET properties table for all tested depsidone derivatives; Table S2: Docking results for all tested compounds against native ligands of CB2 agonist (PDB: 6KPF) Details of molecular dynamics simulation for **E3R**, simplicildone J (**10**), lobaric acid (**110**), mollicellin Q (**101**), garcinisidone E (**215**), mollicellin P (**100**), paucinervin Q (**149**), and boremexin C (**161**); ADMET properties table for all tested depsidone derivatives; docking results for all tested compounds against native ligands of CB2 agonist (PDB: 6KPF).

## References

[1] Brahmachari G. (2011). Natural Products in Drug Discovery: Impacts and Opportunities ? An Assessment. Bioactive Natural Products.

[2] Natural Products in Drug Discovery: Advances and Opportunities|Nature Reviews Drug Discovery. https://www.nature.com/articles/s41573-020-00114-z.

[3] Kiriiri G.K., Njogu P.M., Mwangi A.N. (2020). Exploring different approaches to improve the success of drug discovery and development projects: A review. Future J. Pharm. Sci..

[4] Contemporary Computational Applications and Tools in Drug Discovery|ACS Medicinal Chemistry Letters. https://pubs.acs.org/doi/10.1021/acsmedchemlett.1c00662.

[5] An D., Peigneur S., Hendrickx L.A., Tytgat J. (2020). Targeting Cannabinoid Receptors: Current Status and Prospects of Natural Products. Int. J. Mol. Sci..

[6] Zou S., Kumar U. (2018). Cannabinoid Receptors and the Endocannabinoid System: Signaling and Function in the Central Nervous System. Int. J. Mol. Sci..

[7] Proietto J., Rissanen A., Harp J.B., Erondu N., Yu Q., Suryawanshi S., Jones M.E., Johnson-Levonas A.O., Heymsfield S.B., Kaufman K.D. (2010). A clinical trial assessing the safety and efficacy of the CB1R inverse agonist taranabant in obese and overweight patients: Low-dose study. Int. J. Obes..

[8] Morales P., Hernandez-Folgado L., Goya P., Jagerovic N. (2016). Cannabinoid receptor 2 (CB2) agonists and antagonists: A patent update. Expert Opin. Ther. Pat..

[9] Mannino F., Pallio G., Corsaro R., Minutoli L., Altavilla D., Vermiglio G., Allegra A., Eid A.H., Bitto A., Squadrito F. (2021). Beta-Caryophyllene Exhibits Anti-Proliferative Effects through Apoptosis Induction and Cell Cycle Modulation in Multiple Myeloma Cells. Cancers.

[10] Fuchs A., Rempel V., Müller C.E. (2013). The natural product magnolol as a lead structure for the development of potent cannabinoid receptor agonists. PLoS ONE.

[11] Schuehly W., Paredes J.M.V., Kleyer J., Huefner A., Anavi-Goffer S., Raduner S., Altmann K.H., Gertsch J. (2011). Mechanisms of osteoclastogenesis inhibition by a novel class of biphenyl-type cannabinoid CB(2) receptor inverse agonists. Chem. Biol..

[12] Chianese G., Fattorusso E., Taglialatela-Scafati O., Bavestrello G., Calcinai B., Dien H.A., Ligresti A., Di Marzo V. (2011). Desulfohaplosamate, a new phosphate-containing steroid from *Dasychalina* sp., is a selective cannabinoid CB2 receptor ligand. Steroids.

[13] Thadhani V.M., Karunaratne V. (2017). Potential of Lichen Compounds as Antidiabetic Agents with Antioxidative Properties: A Review. Oxid. Med. Cell. Longev..

[14] White P.A.S., Oliveira R., Oliveira A.P., Serafini M.R., Araújo A.A., Gelain D.P., Moreira J.C., Almeida J.R., Quintans J.S., Quintans-Junior L.J. (2014). Antioxidant activity and mechanisms of action of natural compounds isolated from lichens: A systematic review. Molecules.

[15] Lichens as a Potential Natural Source of Bioactive Compounds: A Review|SpringerLink. https://link.springer.com/article/10.1007/s11101-010-9189-6.

[16] Ibrahim S.R.M., Mohamed G.A., Al Haidari R.A., El-Kholy A.A., Zayed M.F., Khayat M.T. (2018). Biologically active fungal depsidones: Chemistry, biosynthesis, structural characterization, and bioactivities. Fitoterapia.

[17] Singh G., Armaleo D., Dal Grande F., Schmitt I. (2021). Depside and Depsidone Synthesis in Lichenized Fungi Comes into Focus through a Genome-Wide Comparison of the Olivetoric Acid and Physodic Acid Chemotypes of *Pseudevernia furfuracea*. Biomolecules.

[18] Sastry G.M., Adzhigirey M., Day T., Annabhimoju R., Sherman W. (2013). Protein and ligand preparation: Parameters, protocols, and influence on virtual screening enrichments. J. Comput. Aided Mol. Des..

[19] Structure-Based Virtual Screening, Molecular Docking and Dynamics Studies of Natural Product and Classical Inhibitors against Human Dihydrofolate Reductase|SpringerLink. https://link.springer.com/article/10.1007/s13721-020-00244-9.

[20] Philipp D.M., Friesner R.A. (1999). Mixed ab initio QM/MM modeling using frozen orbitals and tests with alanine dipeptide and tetrapeptide. J. Comput. Chem..

[21] Charanya C., Sampathkrishnan S., Balamurugan N. (2017). Quantum mechanical analysis, spectroscopic (FT-IR, FT-Raman, UV-Visible) study, and HOMO-LUMO analysis of (1S,2R)-2-amino-1-phenylpropan-1-ol using Density Functional Theory. J. Mol. Liq..

[22] Adcock S.A., McCammon J.A. (2006). Molecular dynamics: Survey of methods for simulating the activity of proteins. Chem. Rev..

[23] Hollingsworth S.A., Dror R.O. (2018). Molecular dynamics simulation for all. Neuron.

[24] Sargsyan K., Grauffel C., Lim C. (2017). How Molecular Size Impacts RMSD Applications in Molecular Dynamics Simulations. J. Chem. Theory Comput..

[25] Hua T., Li X., Wu L., Iliopoulos-Tsoutsouvas C., Wang Y., Wu M., Shen L., Brust C.A., Nikas S.P., Song F. (2020). Activation and Signaling Mechanism Revealed by Cannabinoid Receptor-Gi Complex Structures. Cell.

[26] (2021). Schrödinger, LigPrep. Schrödinger Release 2021-4.

[27] Glide (2021). Schrödinger Release 2021-4: Glide.

[28] Friesner R.A., Murphy R.B., Repasky M.P., Frye L.L., Greenwood J.R., Halgren T.A., Sanschagrin P.C., Mainz D.T. (2006). Extra Precision Glide:  Docking and Scoring Incorporating a Model of Hydrophobic Enclosure for Protein−Ligand Complexes. J. Med. Chem..

[29] Prime (2022). Schrödinger Release 2022–3: Prime.

[30] Induced Fit Docking (2021). Schrödinger Suite 2009 Induced Fit Docking Protocol.

[31] (2021). Schrödinger Release 2021-4: Desmond Molecular Dynamics System.

[32] Qsite (2021). Schrödinger Release 2022-4.

[33] QikProp (2021). Schrödinger Release 2021-4: QikProp.

