# Supplementary material for: Structure-Based Virtual Screening and Molecular Dynamics Simulation Assessments of Depsidones as Possible Selective Cannabinoid Receptor Type 2 Agonists
Source: Molecules. 2023 Feb 13;28(4):1761. doi: 10.3390/molecules28041761 (PMC9965315; doi:10.3390/molecules28041761)
Supplement: Supplementary file 1 [file molecules-28-01761-s001.zip › molecules-2187374-supplementary.pdf]

Table S1: ADMET properties table for all tested depsidone derivatives

Molecule	#stars	#rtvFG	CNS	mol_MW	SASA	donorHB	accptHB	QPlogPo/w	QPlogHERG	QPPCaco	QPlogBB	#metab	QPlogKhsa	PercentHumanOralAbsorptio
**Recommended Range**	(0.0-5.0)	(0-2)	(-2 inactive) (+2 active)	(130-725)	(300-1000)	(0-6)	(2.0-20.0)	(-2-6.5)	conern below -5	<25 poor, >500 great)	(-3-1.2)	(1-8)	(-1.5-1.5)	(<25% poor; >80% high)
**(2RS)-Creticine**	2	1	-2	494.583	792.204	2	5	5.798	-5.399	464.403	-1.532	13	1.248	95.666
**1-[(E)-but-2-en-2-yl]-2,8-dichloro-3,9-d**	0	1	0	395.238	590.953	2	4.5	3.666	-4.079	665.471	-0.462	6	0.522	100
**11H-Dibenzo[b,e][1,4]dioxepin-11-one,3,8**	0	1	-2	396.439	655.594	3	6.2	2.964	-4.584	248.844	-1.395	8	0.384	87.18
**11H-Dibenzo[b,e][1,4]dioxepin-4-carboxal**	0	1	-2	394.423	644.206	1	5.5	3.414	-4.49	240.188	-1.329	7	0.585	89.544
**1H-Dibenzo[b,e][1,4]dioxepin-11-one,3,8-**	0	1	-2	302.283	493.355	3	6.2	0.858	-3.926	146.762	-1.286	5	-0.291	70.748
**1-Hydroxy-11-(hydroxymethyl)-4,10-**	0	2	-2	402.357	576.632	2	10.9	0.513	-3.635	205.812	-1.24	5	-0.561	71.359
**2,4-Dichlorounguinol**	0	1	0	395.238	587.212	2	4.5	3.483	-4.19	592.919	-0.571	6	0.489	96.972
**2,7-Dichlorounguinol**	0	1	0	409.265	597.064	2	4.5	3.879	-3.896	747.82	-0.401	7	0.608	100
**2-Chloro-7-bromounguinol**	0	1	0	439.689	613.582	2	4.5	3.84	-4.383	676.579	-0.492	6	0.579	100
**2-Chlorounguinol**	0	1	-1	360.793	586.544	2	4.5	3.23	-4.383	520.085	-0.716	6	0.435	94.471
**3,1`-Cichlorounguinol**	0	1	0	395.238	590.953	2	4.5	3.666	-4.079	665.471	-0.462	6	0.522	100
**3,8-Dihydroxy-4-(hydroxymethyl)-9-methyl**	0	1	-2	396.439	652.911	3	6.2	2.958	-4.531	248.741	-1.384	8	0.384	87.147
**3,8-Dihydroxy-4-(methoxymethyl)-9-methyl**	1	1	-1	410.466	669.203	2	6.2	3.721	-4.557	700.331	-0.942	9	0.549	100
**3-Hydroxyphysodic acid**	1	2	-2	486.518	799.75	3	8.25	3.531	-3.71	6.837	-3.225	6	0.149	62.559
**3'-O-Demethylcryptostictinolide**	0	1	-2	358.304	552.468	1	8.2	0.811	-4.06	98.861	-1.524	5	-0.396	67.402
**4,7-Dichlorounguinol**	0	1	0	395.238	581.047	2	4.5	3.443	-4.07	613.813	-0.55	6	0.478	100
**4-Formyl-3,8-dihydroxy-9-methyl-1,6-bis(**	0	1	-2	394.423	640.508	1	5.5	3.405	-4.415	241.184	-1.312	7	0.583	89.521
**4-Methylunguinol**	0	1	-1	340.375	576.172	2	4.5	3.009	-4.238	547.52	-0.802	7	0.427	93.576
**7-Bromofolipastatin**	0	1	0	459.336	646.86	2	4.5	4.391	-4.165	727.606	-0.632	8	0.859	100
**7-Bromounguinol**	0	1	0	405.244	578.108	2	4.5	3.28	-4.13	573.904	-0.641	6	0.453	95.531
**7-Carboxyfolipastatin**	0	1	-2	424.449	663.536	2	5.5	3.966	-2.506	35.864	-1.543	8	0.553	77.993
**7-Chlorofolipastatin=Emeguisin A**	0	1	0	414.885	646.469	2	4.5	4.337	-4.201	716.928	-0.654	8	0.845	100
**8`-O-Ethylstictic acid**	0	2	-2	414.368	633.419	0	10.2	0.642	-4.41	149.315	-1.542	4	-0.957	69.62
**8`-O-Methylstictic acid**	0	2	-2	400.341	592.297	0	10.2	0.167	-4.056	140.724	-1.416	4	-1.118	66.372
**8'-O-methylprotocetraric acid**	0	1	-2	388.33	584.779	1	8.2	1.353	-2.296	7.493	-2.177	6	-0.468	50.52
**8'-O-methylsalazinic acid**	0	2	-2	402.314	570.024	1	10.9	-0.494	-3.935	20.064	-2.268	5	-0.864	47.366
**Aspergillusidone A**	0	1	-2	370.358	591.807	2	5.5	2.831	-2.42	27.708	-1.539	6	0.149	69.342
**Aspergillusidone B**	0	1	0	409.265	609.69	1	4.5	4.28	-4.152	1718.34	-0.149	6	0.681	100
**Aspergillusidone C**	0	1	0	395.238	590.953	2	4.5	3.666	-4.079	665.472	-0.462	6	0.522	100
**Aspergillusidone D**	0	1	0	484.14	598.168	2	4.5	3.81	-4.119	684.99	-0.43	6	0.561	100
**Aspergillusidone E**	0	1	0	405.244	590	2	4.5	3.3	-4.398	527.419	-0.7	6	0.454	94.992

**Aspergillusidone F**	0	1	0	484.14	584.741	2	4.5	3.565	-4.059	642.081	-0.507	6	0.508	100
**Aspergisidone**	0	1	-2	424.449	665.005	2	5.5	3.97	-2.531	35.873	-1.549	8	0.554	78.017
**Aspersidone**	0	1	0	409.265	609.458	1	4.5	4.374	-4.062	1539.611	-0.134	6	0.704	100
**Aspersidone B**	0	1	0	374.82	618.966	1	4.5	4.076	-4.518	1406.769	-0.323	6	0.646	100
**Asperunguissidone A**	0	1	-2	342.348	560.584	3	6.2	1.764	-4.229	198.376	-1.31	5	-0.028	78.394
**Asperunguissidone B**	0	1	-2	376.793	587.506	3	6.2	2.326	-4.329	276.876	-1.082	5	0.071	84.28
**Auranticin A**	0	2	-2	440.449	732.749	3	8.2	3.173	-3.211	21.426	-2.153	7	0.134	69.348
**Auranticin B**	0	2	-2	438.433	685.458	1	7.5	3.243	-2.827	19.867	-1.997	6	0.155	69.168
**Bailesidone**	0	2	-2	414.368	640.153	1	9.25	1.368	-4.543	107.834	-1.716	7	-0.252	71.341
**Baillonic acid**	0	1	-2	374.303	578.103	2	8.5	1.823	-0.841	5.859	-1.66	3	-0.644	51.363
**Boremexin A**	0	2	-2	398.368	625.758	1	7.25	2.534	-4.817	341.097	-1.063	5	0.179	87.114
**Boremexin B**	0	2	-2	398.368	625.758	1	7.25	2.534	-4.817	341.097	-1.063	5	0.179	87.114
**Boremexin C**	0	2	-2	416.384	650.358	3	8.95	1.511	-4.765	85.053	-1.935	6	-0.131	70.328
**Botryorhodine A=Botryosphaerone A**	0	1	-2	300.267	485.886	1	5.5	1.177	-3.897	119.953	-1.291	4	-0.19	71.045
**Botryorhodine B=Botryosphaerone B**	0	1	-2	314.294	511.733	1	5.5	1.561	-3.88	158.075	-1.218	5	-0.053	75.444
**Botryorhodine C=Botryosphaerone C**	0	1	-2	316.31	519.61	3	6.2	1.224	-3.918	194.777	-1.21	6	-0.177	75.088
**Botryorhodine D=Botryosphaerone D**	0	1	-2	302.283	493.355	3	6.2	0.858	-3.926	146.762	-1.286	5	-0.291	70.748
**Botryorhodine E**	0	1	-2	288.256	474.673	3	5.25	1.005	-3.938	192.591	-1.114	5	-0.241	73.722
**Botryorhodine F**	0	1	-2	302.283	501.393	3	5.25	1.378	-3.935	257.659	-1.034	6	-0.122	78.165
**Botryorhodine G**	0	1	-1	316.31	521.727	2	6.2	1.694	-4.087	455.616	-0.879	6	-0.145	84.448
**Botryorhodine H**	0	1	-1	441.277	628.313	2	4.5	4.04	-5.448	529.157	-0.763	5	0.609	100
**Botryorhodine I**	0	1	-2	318.282	498.346	4	6.95	0.35	-3.748	80.774	-1.565	6	-0.405	63.132
**Ceratinalone**	0	2	-2	402.357	621.9	1	8.95	1.554	-4.444	256.039	-1.312	5	-0.278	79.148
**Cetraric acid**	0	1	-2	402.357	619.114	1	8.2	1.726	-2.551	8.06	-2.306	6	-0.37	53.271
**Chaetosidone A = Corynesidone D**	0	1	-2	316.267	499.699	2	5.5	1.507	-2.131	14.455	-1.601	4	-0.273	56.531
**Conhypoprotocetraric acid**	0	1	-2	360.32	557.838	3	7.2	1.222	-2.258	8.405	-2.048	6	-0.358	50.648
**Conneotricone**	0	1	-2	388.287	537.372	2	9.2	0.152	-1.854	2.618	-2.376	5	-0.674	35.313
**Connorstictic acid**	0	1	-2	356.331	541.521	2	8.45	0.879	-3.801	161.997	-1.206	5	-0.299	71.639
**Cordidepsine**	0	1	-2	330.293	536.456	1	6.25	1.564	-4.144	214.568	-1.223	5	-0.151	77.833
**Cordycepsidone A=Excelsional**	0	1	-2	356.288	520.774	0	7.5	0.261	-3.573	50.491	-1.619	5	-0.758	58.959
**Cordycepsidone B**	0	1	-2	372.287	534.678	1	7.5	1.132	-1.938	8.953	-1.802	5	-0.432	50.609
**Corynesidone A**	0	1	-1	272.257	469.182	2	4.5	1.519	-4.012	330.461	-0.848	4	-0.097	80.926
**Corynesidone B**	0	1	-2	332.266	507.062	3	6.25	0.932	-2.047	6.563	-1.974	5	-0.4	47.026
**Corynesidone C**	0	1	-2	288.256	477.316	3	5.25	0.932	-3.936	148.029	-1.222	5	-0.235	71.246
**Corynesidone E**	0	1	-1	302.283	509.997	2	5.25	1.75	-4.178	442.899	-0.854	5	-0.074	84.555
**Cryptostictic acid**	0	2	-2	388.33	561.317	2	9.9	0.311	-3.688	75.978	-1.634	5	-0.505	62.427
**Curdepsidone A**	0	1	-2	332.266	528.485	1	6	1.137	-4.206	57.976	-1.811	5	-0.2	65.162
**Curdepsidone B**	0	2	-2	448.426	666.085	3	10.65	1.381	-4.469	103.727	-1.906	8	-0.289	71.113
**Curdepsidone C**	0	2	-2	448.426	681.097	3	10.65	1.433	-4.669	110.911	-1.943	8	-0.286	71.938
**Curdepsidone D**	0	2	-2	462.452	717.196	3	10.65	1.853	-4.929	126.222	-2.016	8	-0.195	75.402

**Curdepsidone E**	0	2	-2	462.452	717.82	3	10.65	1.816	-4.974	129.687	-2.02	8	-0.215	75.394
**Curdepsidone F**	0	1	-1	302.283	495.309	2	5.25	1.697	-3.901	431.111	-0.813	5	-0.086	84.035
**Curdepsidone G**	0	1	-2	346.336	552.179	2	6.95	1.718	-4.036	371.266	-1.042	6	-0.143	82.999
**Diaporthol B**	0	1	-1	368.385	593.609	2	6.2	2.687	-4.545	576.166	-0.792	4	0.251	92.085
**Diffractione B**	0	2	-2	444.394	667.445	0	10.9	0.534	-4.588	61.333	-2.147	5	-1.048	62.071
**Dioxepin-11-one**	0	1	0	300.31	505.844	2	4.5	2.168	-3.783	544.107	-0.666	6	0.129	88.605
**Emeguisin B**	1	1	0	428.911	680.548	1	4.5	5.193	-4.385	1919.282	-0.268	8	1.069	100
**Emeguisin C**	1	1	0	463.357	686.95	1	4.5	5.507	-4.313	2057.369	-0.132	8	1.131	100
**Emeguisin D**	0	1	0	449.33	665.828	2	4.5	4.798	-4.24	891.371	-0.463	8	0.94	100
**Excelsione=Phomopsidone**	0	1	-2	358.304	523.153	2	8.2	0.665	-3.539	93.652	-1.435	6	-0.366	66.126
**Flavicansone**	0	1	1	383.227	572.681	0	4.5	4.129	-3.925	3905.263	0.336	5	0.369	100
**Folipastatin**	0	1	-1	380.44	643.982	2	4.5	3.922	-4.545	611.583	-0.876	8	0.752	100
**Fumarprotocetraric acid**	3	3	-2	472.361	662.526	2	10.5	1.025	-0.772	0.071	-3.827	5	-0.644	0
**Fumarprotocetraric acid lactone 1**	3	3	-2	502.344	639.77	3	13.2	-0.106	-0.456	0.075	-3.681	5	-1.074	0
**Fumarprotocetraric acid lactone 2**	3	3	-2	486.345	693.236	2	11.5	0.581	-1.386	0.056	-4.198	5	-0.8	0
**Galbinic acid**	2	3	-2	430.324	597.901	1	11.2	-0.609	-4.016	5.943	-2.791	4	-0.767	24.275
**Gangaleoidin**	0	2	0	413.21	611.588	1	6.5	2.97	-4.521	518.191	-0.652	4	0.2	92.919
**Garcinisidone A**	1	1	-2	426.465	705.696	2	5	4.307	-5.112	367.052	-1.426	10	0.786	100
**Garcinisidone B**	0	1	-1	424.449	675.281	1	5	4.426	-5.056	682.18	-0.915	6	0.846	100
**Garcinisidone C**	1	1	-2	476.525	764.741	1	5	5.489	-5.624	529.127	-1.063	5	1.367	94.872
**Garcinisidone D**	1	1	-1	476.525	752.671	1	5	5.529	-5.459	741.828	-0.876	5	1.342	100
**Garcinisidone E**	2	1	-2	478.541	778.681	2	5	5.471	-5.428	603.731	-1.194	9	1.25	95.791
**Glomellonic acid**	3	3	-2	458.334	660.934	2	11	0.579	-1.16	0.086	-3.873	4	-0.833	0
**Guanxidone A**	0	1	-2	372.331	557.347	1	8.2	1.229	-3.813	218.704	-1.173	7	-0.315	76.023
**Guanxidone B**	0	1	-2	342.304	517.384	1	6.5	1.399	-3.571	200.385	-1.032	6	-0.109	76.337
**Himantormione A**	0	1	-2	356.418	618.564	2	4.5	3.524	-4.595	439.007	-1.236	4	0.442	94.872
**Himantormione B**	0	1	-2	384.471	715.746	2	4.5	4.356	-5.504	375.511	-1.619	4	0.713	100
**Hyperwightin A**	0	1	0	324.332	541.842	1	4.5	2.975	-4.527	822.973	-0.486	2	0.372	96.547
**Hypoprotocetraric acid**	0	1	-2	344.32	546.63	2	5.5	2.189	-2.185	24.104	-1.477	6	-0.048	64.499
**Lasiodiplodiaone A**	0	1	-2	374.39	607.593	3	7.9	1.816	-4.536	238.871	-1.409	7	-0.13	80.144
**Lasiodiplodiaone B**	0	1	-2	388.416	635.537	3	7.9	2.247	-4.536	372.449	-1.256	8	-0.006	86.121
**Livistone A**	0	1	-2	302.283	493.355	3	6.2	0.858	-3.926	146.762	-1.286	5	-0.291	70.748
**Livistone B**	0	1	-2	316.31	519.61	3	6.2	1.224	-3.918	194.777	-1.21	6	-0.177	75.088
**Lobaric acid**	0	1	-2	456.491	782.184	1	7.5	4.226	-3.811	28.355	-2.352	4	0.325	77.691
**Maldoxone**	0	2	-1	364.738	587.467	0	5.5	2.713	-4.714	502.526	-0.768	3	-0.01	91.176
**Menegazziaic acid**	0	2	-2	374.303	525.265	3	9.9	-0.339	-3.42	30.318	-1.886	5	-0.542	51.482
**Methyl psoromate**	0	2	-2	372.331	592.293	0	7.5	1.511	-4.32	209.009	-1.291	4	-0.45	77.321
**Mollicellin A.**	0	1	-2	382.369	594.493	0	7.5	1.61	-4.321	188.461	-1.192	4	-0.317	77.092
**Mollicellin B**	0	1	-2	382.369	604.248	0	7.5	1.705	-4.298	177.331	-1.23	4	-0.257	77.178
**Mollicellin C**	0	2	-2	412.395	642.265	1	8.25	1.984	-4.49	120.397	-1.692	7	-0.031	75.803

**Mollicellin D**	0	1	-2	404.846	636.698	3	6.2	2.949	-4.49	317.987	-1.142	8	0.265	89.004
**Mollicellin E**	0	2	-2	446.84	653.625	1	8.25	2.418	-4.349	140.94	-1.485	7	0.054	79.565
**Mollicellin F**	0	1	-2	432.813	611.589	1	8.25	1.829	-3.931	86.457	-1.408	5	0.014	72.322
**Mollicellin G**	0	1	-2	368.385	608.169	1	5.5	2.777	-4.559	163.948	-1.471	7	0.351	82.842
**Mollicellin H**	0	1	-2	368.385	621.833	1	5.5	2.839	-4.66	151.245	-1.548	7	0.392	82.58
**Mollicellin I**	0	1	-2	370.401	620.024	3	6.2	2.503	-4.526	273.215	-1.329	8	0.182	85.208
**Mollicellin J**	0	1	-2	402.83	636.034	1	5.5	3.287	-4.575	174.519	-1.355	7	0.483	86.317
**Mollicellin K**	0	2	-2	382.369	631.973	0	6.5	2.14	-4.742	77.056	-1.87	6	-0.031	73.245
**Mollicellin L**	0	2	-2	396.396	661.747	0	7.5	2.359	-4.828	212.461	-1.462	6	-0.12	82.414
**Mollicellin M**	0	1	-2	416.814	622.057	0	7.5	2.178	-4.343	236.271	-1.007	4	-0.172	82.176
**Mollicellin N**	0	1	-2	398.368	604.436	1	8.25	1.373	-4.213	74.757	-1.644	5	-0.085	68.517
**Mollicellin O**	1	1	-1	398.455	666.498	2	6.2	3.656	-4.662	806.954	-0.953	9	0.456	100
**Mollicellin P**	0	1	-2	430.454	702.313	3	9.6	2.213	-5.061	403.047	-1.284	7	-0.021	86.531
**Mollicellin Q**	0	1	-2	414.454	698.882	2	7.9	3.048	-5.079	657.833	-1.004	7	0.283	95.231
**Mollicellin R**	0	1	-1	366.37	599.977	0	5.5	2.813	-4.661	365.578	-0.95	3	0.207	89.29
**Mollicellin S**	0	1	-1	416.857	669.772	0	5.5	3.959	-4.726	530.025	-0.912	7	0.479	100
**Mollicellin T**	0	1	-1	418.873	681.463	2	6.2	3.721	-4.836	575.306	-0.957	8	0.484	100
**Mollicellin U**	0	1	-1	340.375	599.653	2	4.5	3.171	-4.796	492.557	-0.967	7	0.437	93.701
**Mollicellin V**	0	1	-2	384.385	628.388	0	6.5	2.239	-4.576	98.806	-1.787	5	-0.059	75.759
**Mollicellin W**	0	1	-2	412.438	663.511	0	7.5	2.685	-4.466	278.567	-1.34	6	-0.045	86.427
**Mollicellin X**	0	1	-2	446.883	656.397	0	7.5	3.01	-4.044	307.23	-1.101	6	0.007	89.092
**Mollicellin Y**	0	2	-2	412.438	661.767	2	8.2	2.524	-4.456	306.42	-1.297	8	0.146	86.224
**Neotricone**	0	1	-2	372.287	524.945	1	7.5	1.045	-1.758	7.926	-1.796	5	-0.446	49.155
**Nidulin**	0	1	0	443.71	616.284	1	4.5	4.607	-4.022	1615.222	-0.043	6	0.755	100
**Norcolensoic acid**	0	1	-2	428.481	720.93	2	5.5	4.324	-3.332	22.931	-2.281	4	0.474	76.61
**Norlobaridone**	0	1	-2	398.455	696.314	2	6.5	3.292	-5.141	172.874	-1.896	4	0.353	86.273
**Nornidulin S15**	1	1	0	509.813	712.405	1	4.5	5.935	-4.802	1623.793	-0.179	7	1.262	93.243
**Nornidulin S16**	1	1	0	519.808	734.216	1	4.5	6.295	-5.591	1617	-0.259	7	1.307	95.314
**Nornidulin S17**	3	1	0	545.846	806.273	1	4.5	7.075	-6.265	1623.081	-0.381	7	1.578	100
**Nornidulin S18**	12	2	-2	913.458	1164.158	2	9	9.661	-6.524	264.782	-1.597	12	2.484	100
**Nornidulin S19**	2	1	0	520.796	727.493	1	6	5.34	-5.381	872.976	-0.533	9	0.961	84.932
**Nornidulin S20**	2	1	0	520.796	728.211	1	6	5.342	-5.398	873.462	-0.534	9	0.961	84.95
**Nornidulin S25**	1	2	0	533.791	758.179	1	6.25	5.535	-5.885	869.882	-0.513	5	1.048	86.049
**Nornidulin S26**	1	1	-2	550.779	750.864	1	5.25	5.372	-5.429	192.713	-1.258	6	1.204	73.381
**Nornidulin S27**	1	1	-2	568.769	744.445	1	5.25	5.395	-5.243	192.514	-1.2	6	1.205	73.505
**Nornidulin S28**	2	1	-2	585.224	764.945	1	5.25	5.78	-5.252	192.257	-1.126	6	1.309	75.749
**Nornidulin S29**	2	1	-1	618.777	788.962	1	5.25	6.367	-5.349	266.685	-0.892	6	1.445	81.729
**Nornidulin S30**	1	1	-2	550.779	745.515	1	5.25	5.449	-5.421	249.136	-1.12	6	1.197	75.824
**Nornidulin S31**	1	1	-1	548.781	744.022	1	5.75	5.338	-5.402	336.105	-0.953	5	1.078	77.501
**Nornidulin S32**	1	1	-2	551.766	751.901	1	6.25	4.865	-5.458	174.137	-1.321	7	0.976	82.58

**Nornidulin S5**	1	1	0	485.791	719.099	1	4.5	5.777	-4.744	1615.851	-0.314	6	1.127	100
**Nornidulin S6**	1	1	0	499.817	752.069	1	4.5	6.162	-4.943	1616.321	-0.399	6	1.249	100
**Nornidulin S8**	3	1	-1	569.951	912.948	1	4.5	8.043	-5.778	1612.509	-0.82	6	1.847	100
**Nornidulin S9**	9	1	-2	682.166	1174.471	1	4.5	11.14	-6.766	1618.884	-1.486	6	2.862	100
**Nornidulin=Ustin**	0	1	0	429.683	597.482	2	4.5	3.896	-4.03	700.651	-0.369	6	0.57	100
**Norperistictic acid**	0	2	-2	388.287	540.648	2	9.2	0.239	-1.987	3.57	-2.233	4	-0.643	38.236
**Norstictic acid**	0	2	-2	372.287	529.863	1	9.2	-0.308	-3.694	18.864	-2.086	4	-0.644	47.972
**Oxolobaric acid**	0	2	-2	470.475	760.598	1	9.5	2.929	-3.469	9.26	-2.793	5	-0.121	61.394
**Parmosidone A**	0	1	-2	374.303	548.591	2	8.2	0.71	-2.02	3.861	-2.346	5	-0.544	41.602
**Parmosidone B**	0	1	-2	362.292	540.712	4	7.95	0.545	-2.128	5.738	-2.215	6	-0.572	43.717
**Parmosidone C**	2	2	-2	552.49	733.965	2	9	3.153	-2.532	3.942	-2.723	9	0.285	30.155
**Parmosidone D**	3	2	-2	538.464	747.809	2	9	2.774	-3.076	1.639	-3.273	8	0.214	21.11
**Parmosidone E**	0	1	-2	358.304	545.906	1	6.5	1.538	-2.12	7.309	-1.999	5	-0.264	51.411
**Parmosidone F**	0	2	-2	428.395	664.45	2	9.2	1.981	-2.881	12.333	-2.268	6	-0.321	58.072
**Parmosidone F1**	2	2	-2	494.411	626.227	3	10.7	0.275	-4.014	5.114	-2.861	7	-0.286	28.28
**Parmosidone G**	1	2	-2	508.481	736.962	2	8	2.909	-4.824	21.856	-2.644	9	0.548	54.994
**Parmosidone G**	1	2	-2	508.481	765.452	2	8	3.099	-5.334	30.468	-2.664	9	0.562	58.691
**Parmosidone G1**	2	3	-2	534.475	731.786	4	11.7	0.985	-5.066	9.428	-3.123	7	-0.203	24.234
**Parmosidone H**	1	3	-2	548.545	796.764	3	9	3.627	-5.177	97.705	-2.186	9	0.56	70.838
**Parmosidone I**	3	2	-2	566.474	779.787	1	10	2.302	-3.248	0.428	-4.08	8	0.06	7.899
**Parmosidone J**	0	2	-2	428.395	628.457	2	7.95	2.519	-2.606	32.255	-1.558	5	-0.078	68.696
**Parmosidone K**	2	1	-2	480.427	700.089	3	8	2.171	-3.3	1.356	-3.308	8	0.058	42.021
**Paucinervin Q**	1	1	-2	426.465	733.431	3	6	3.82	-5.24	307.837	-1.578	10	0.607	93.85
**Perisalazinic acid**	2	2	-2	404.286	551.996	3	10.9	-0.65	-2.057	1.179	-2.822	4	-0.863	11.459
**Peristictic acid**	0	2	-2	402.314	574.963	2	10.2	0.499	-2.177	9.1	-1.953	4	-0.677	47.034
**Phomopsidone A**	0	1	-1	340.289	489.73	0	7.75	0.57	-3.276	500.181	-0.571	5	-0.881	78.592
**Physodalic acid**	0	2	-2	402.314	582.525	1	9	0.843	-2.203	3.81	-2.357	4	-0.565	42.278
**Pilobolusone A**	1	1	-2	424.493	707.702	2	6.2	4.161	-4.789	717.796	-1.034	9	0.684	100
**Pilobolusone B**	0	1	-1	370.401	603.537	2	6.2	2.857	-4.223	606.269	-0.881	8	0.257	93.48
**Pilobolusone C**	0	2	-2	454.476	709.359	2	8.2	3.613	-2.818	57.635	-1.587	8	0.169	79.614
**Pilobolusone D**	1	2	-2	468.502	748.91	2	8.2	3.539	-4.932	195.85	-1.713	9	0.519	88.69
**Polyanthadepsidone A**	0	1	-1	346.336	547.247	2	5	2.421	-3.742	445.098	-0.897	8	0.193	88.522
**Protocetraric acid**	0	1	-2	374.303	557.125	2	8.2	0.596	-2.181	2.422	-2.589	5	-0.537	37.312
**Psoromic acid**	0	1	-2	358.304	560.103	1	7.5	1.539	-2.358	18.285	-1.688	4	-0.414	58.544
**Psoromic acid'**	0	1	-2	358.304	560.103	1	7.5	1.539	-2.358	18.285	-1.688	4	-0.414	58.544
**Purpactin A**	0	2	-1	414.454	692.936	1	7	3.745	-4.992	796.989	-0.933	5	0.465	100
**Purpactin C`**	0	2	-2	412.438	699.412	0	7.75	2.935	-5.099	317.12	-1.377	3	0.01	88.895
**Salazinic acid**	2	2	-2	388.287	542.953	2	10.9	-1.149	-3.806	6.213	-2.682	4	-0.822	34.418
**Salazinic acid**	2	2	-2	388.287	539.161	2	10.9	-1.106	-3.719	7.123	-2.594	4	-0.817	35.731
**Salazinin A**	0	2	-2	416.34	619.273	1	10.9	0.201	-4.253	27.257	-2.309	5	-0.678	53.816

**Simpliciildone A**	0	1	-1	330.337	548.478	2	6.2	2.072	-4.087	601.581	-0.796	7	-0.018	88.824
**Simpliciildone B**	0	1	-1	344.363	575.599	2	6.2	2.404	-4.254	609.27	-0.878	7	0.067	90.864
**Simpliciildone C**	0	2	0	356.374	574.937	1	5.25	3.199	-4.203	1458.321	-0.274	5	0.423	100
**Simpliciildone D**	1	1	-2	436.46	685.475	3	6	3.63	-5.131	400.404	-1.226	9	0.555	94.782
**Simpliciildone E**	1	1	-2	436.46	682.014	3	6	3.536	-5.001	273.06	-1.372	9	0.571	91.253
**Simpliciildone F**	2	3	-2	450.444	694.172	3	9.25	1.868	-4.89	64.005	-1.995	9	0.124	70.208
**Simpliciildone G**	1	2	-2	426.465	710.926	2	8.2	2.908	-5.074	251.147	-1.496	9	0.3	86.929
**Simpliciildone H**	1	2	-2	614.604	840.679	4	10.7	3.406	-5.609	53.125	-2.376	13	0.595	51.849
**Simpliciildone I**	1	1	-2	528.557	761.918	4	6.5	4.303	-5.38	147.556	-1.799	10	0.854	78.002
**Simpliciildone J**	0	1	-2	420.461	708.462	2	6.2	4.133	-6.007	660.471	-1.12	8	0.578	100
**Simpliciildone K**	2	2	-2	546.616	800.43	3	6.75	5.061	-5.102	414.679	-1.205	10	1.208	77.514
**Siphulellic acid**	0	2	-2	402.314	624.423	2	9.5	0.656	-2.966	2.02	-2.948	4	-0.549	36.251
**Spiromastixone A**	0	1	-2	328.364	569.887	2	4.5	2.864	-4.38	413.186	-1.096	4	0.26	90.537
**Spiromastixone B**	0	1	-1	362.809	602.176	2	4.5	3.352	-4.706	469.748	-0.988	4	0.365	94.395
**Spiromastixone C**	0	1	-2	362.809	591.221	2	4.5	3.205	-4.54	451.864	-1.007	4	0.332	93.234
**Spiromastixone D**	0	1	-1	397.254	619.021	2	4.5	3.758	-4.679	541.673	-0.832	4	0.454	100
**Spiromastixone E**	0	1	-1	397.254	610.557	2	4.5	3.692	-4.587	542.496	-0.826	4	0.436	100
**Spiromastixone F**	0	1	0	431.699	615.724	2	4.5	4.073	-4.408	652.331	-0.607	4	0.501	100
**Spiromastixone G**	0	1	0	445.726	653.171	1	4.5	4.977	-4.593	1804.647	-0.204	4	0.738	100
**Spiromastixone H**	0	1	0	431.699	632.037	2	4.5	4.232	-4.565	687.754	-0.59	4	0.543	100
**Spiromastixone I**	1	1	0	466.144	649.459	2	4.5	4.672	-4.531	800.581	-0.41	4	0.636	100
**Spiromastixone J**	1	1	0	480.171	647.849	1	4.5	5.247	-4.231	1933.119	-0.038	4	0.787	100
**Spiromastixone K**	0	1	0	445.726	660.203	1	4.5	5.112	-4.627	2003.034	-0.136	4	0.765	100
**Spiromastixone L**	0	1	0	480.171	653.417	1	4.5	5.267	-4.332	2018.426	-0.054	4	0.803	100
**Spiromastixone M**	0	1	-1	397.254	617.925	2	4.5	3.808	-4.6	595.303	-0.767	4	0.456	100
**Spiromastixone N**	0	1	0	431.699	603.217	2	4.5	4.16	-4.061	649.301	-0.512	4	0.517	100
**Spiromastixone O**	1	1	0	466.144	647.056	2	4.5	4.658	-4.503	827.258	-0.414	4	0.642	100
**Spiromastixone P**	0	1	-1	407.26	604.019	2	4.5	3.436	-4.62	456.775	-0.974	4	0.398	94.666
**Spiromastixone P!**	0	1	-1	362.809	601.011	2	4.5	3.368	-4.609	449.155	-0.992	4	0.38	94.14
**Spiromastixone Q**	0	1	-1	407.26	603.552	2	4.5	3.491	-4.534	496.314	-0.908	4	0.399	95.633
**Spiromastixone Q1**	0	1	-1	362.809	599.275	2	4.5	3.41	-4.507	483.174	-0.928	4	0.378	94.954
**Spiromastixone R**	0	1	-1	407.26	598.617	2	4.5	3.333	-4.63	474.784	-0.984	4	0.366	94.364
**Spiromastixone R1**	0	1	0	431.699	609.827	2	4.5	4.115	-4.218	723.241	-0.508	4	0.491	100
**Spiromastixone S**	0	1	-1	486.156	606.258	2	4.5	3.767	-4.402	561.706	-0.757	4	0.451	100
**Spiromastixone S1**	0	1	-2	372.374	621.511	2	5.5	2.957	-2.956	19.433	-1.981	4	0.095	67.321
**Spiromastixone T**	0	1	0	500.183	642.859	1	4.5	4.671	-4.633	1562.563	-0.356	4	0.68	100
**Stictic acid**	0	2	-2	386.314	565.452	1	10.2	-0.05	-3.915	48.413	-1.809	4	-0.689	56.811
**Succinprotocetraric acid**	3	2	-2	474.377	642.136	2	10.5	1.1	-0.32	0.111	-3.456	7	-0.647	3.344
**Talaromyone A**	0	1	-1	372.417	631.062	2	7.15	2.779	-4.704	706.038	-0.992	4	0.086	94.204
**Unguinol=Tridechloronornidulin**	0	1	-1	326.348	569.211	2	4.5	2.778	-4.388	443.257	-0.905	6	0.35	90.582

**Variolaric acid**	0	1	-2	314.251	481.973	1	6.5	0.728	-3.855	119.348	-1.227	4	-0.344	68.377
**Vicanicin**	0	1	0	369.201	539.022	2	4.5	3.075	-3.726	724.262	-0.303	6	0.307	96.14

Table S2: Docking results for all tested compounds against native ligands of CB2 agonist (PDB: 6KPF)

Title	PubChem CID/ChemSpider ID	docking score	XP GScore	glide gscore	glide emodel
6KPF - prepared	-				
6KPF - prepared_ligand	-	-12.240	-12.240	-12.240	-79.066
Simplicildone J	146683462	-12.134	-12.174	-12.174	-17.822
Lobaric acid	73157	-11.944	-11.944	-11.944	-37.511
Mollicellin Q	146684102	-11.479	-11.513	-11.513	55.427
Garcinisidone E	10838374	-11.394	-11.633	-11.633	15.908
Mollicellin P	146684101	-11.322	-11.356	-11.356	-16.759
Paucinervin Q	-	-11.305	-11.542	-11.542	-23.918
Boremexin C	156582387	-11.254	-11.376	-11.376	-12.854
Mollicellin P	146684101	-11.020	-11.054	-11.054	-19.112
Garcinisidone F	8888728*	-11.019	-11.514	-11.514	7.508
Garcinisidone F	8888728*	-10.910	-11.359	-11.359	-28.677
Norlobaridone	233380	-10.804	-11.053	-11.053	-79.671
7-Chlorofolipastatin = Emeguisin A	14164492	-10.741	-11.433	-11.433	35.197
Simplicildone E	139590884	-10.711	-11.144	-11.144	-101.506
Lasiodiplodiaone A	-	-10.674	-10.729	-10.729	-37.464
Diaporthol B	139591243	-10.628	-10.639	-10.639	-32.493
Mollicellin O	146684100	-10.614	-10.657	-10.657	-37.814
Himantormione B	-	-10.578	-10.636	-10.636	-67.389
Spiromastixone S1	-	-10.569	-10.599	-10.599	-13.643
Mollicellin I	24787299	-10.535	-10.573	-10.573	-47.218
Garcinisidone A	10741092	-10.518	-10.796	-10.796	-68.683
Menegazziaic acid	71438918	-10.512	-11.356	-11.356	40.590
Himantormione A	-	-10.494	-10.552	-10.552	-30.135
3,8-Dihydroxy-4-(hydroxymethyl)-9-methyl-1,6-bis(1-methyl-1-propenyl)-11H-dibenzo[b,e][1,4]dioxepin-11-one	-	-10.466	-10.521	-10.521	28.903
Corynesidone B	78434980*	-10.460	-10.510	-10.510	-47.345
Lasiodiplodiaone B	-	-10.456	-10.492	-10.492	-20.127
Norlobaridone	233380	-10.372	-11.013	-11.013	-76.779
Pilobolusone A	-	-10.370	-10.433	-10.433	34.445
Polyanthadepsidone A	-	-10.362	-10.437	-10.437	-15.492
Botryorhodine I	156582113	-10.340	-11.034	-11.034	-32.233
Mollicellin T	-	-10.328	-10.818	-10.818	-10.050
Asperunguissidone A	109107853*	-10.301	-10.366	-10.366	-12.591
7-Chlorofolipastatin = Emeguisin A	14164492	-10.286	-10.540	-10.540	30.056
3-Hydroxyphysodic acid	171308	-10.279	-10.390	-10.390	-22.700
Spiromastixone A	90670406	-10.204	-10.263	-10.263	-60.098
Spiromastixone P1	-	-10.165	-10.607	-10.607	-25.082
Pilobolusone B	-	-10.165	-10.207	-10.207	27.455
Parmosidone B	132553221	-10.158	-10.275	-10.275	-34.757
11H-Dibenzo[b,e][1,4]dioxepin-11-one,3,8-dihydroxy-4-	-	-10.130	-10.185	-10.185	29.474

Title	PubChem CID/ChemSpider ID	docking score	XP GScore	glide gscore	glide emodel
(hydroxymethyl)-9-methyl-1,6-bis[(1E)-1-methyl-1-propen-1-yl]					
Botryorhodine E	122214817	-10.086	-10.214	-10.214	-51.416
Livistone A	-	-10.053	-10.106	-10.106	-41.581
Menegazziaic acid	71438918	-10.037	-10.881	-10.881	1.322
Botryorhodine G	122214818	-10.030	-10.087	-10.087	-47.863
Emeguisin C	-	-10.028	-10.349	-10.349	315.944
7-Bromofolipastatin	146684025	-10.005	-10.258	-10.258	39.897
Oxolobaric acid	139584339	-9.986	-9.986	-9.986	-19.659
Garcinisidone B	10526339	-9.974	-10.136	-10.136	37.153
1H-Dibenzo[b,e][1,4]dioxepin-11-one,3,8-dihydroxy-4-(methoxymethyl)-1,6-dimethyl	-	-9.911	-9.964	-9.964	-42.929
Mollicellin T	-	-9.905	-10.246	-10.246	-2.287
Corynesidone C	60154238	-9.874	-9.957	-9.957	-53.736
11H-Dibenzo[b,e][1,4]dioxepin -11-one,3,8-dihydroxy-4-(methoxymethyl)-9-methyl-1,6-bis[(1E)-1-methyl-1-propen-1-yl	-	-9.855	-9.916	-9.916	97.000
Mollicellin V	-	-9.853	-11.572	-11.572	-40.150
Mollicellin D	152840	-9.839	-10.155	-10.155	-17.637
Corynesidone E	132512651	-9.829	-9.875	-9.875	-51.451
Curdepsidone D	146682938	-9.818	-9.868	-9.868	512.756
Curdepsidone F	146682940	-9.763	-9.825	-9.825	-57.491
Simplicildone G	139590881	-9.751	-9.792	-9.792	15.670
4-Formyl-3,8-dihydroxy-9-methyl-1,6-bis(1-methyl-1-propenyl)-11H-dibenzo[b,e][1,4] dioxepin-11-one	-	-9.745	-9.831	-9.831	46.610
3,8-Dihydroxy-4-(methoxymethyl) -9-methyl-1,6-bis(1-methyl-1-propenyl)-11Hdibenzo[b,e][1,4] dioxepin-11-one	-	-9.735	-9.795	-9.795	84.369
Botryorhodine D = Botryosphaerone D	42637419	-9.733	-9.786	-9.786	-43.265
Nornidulin S15	-	-9.716	-9.732	-9.732	28.680
Physodic acid	65751	-9.674	-9.699	-9.699	-40.465
Mollicellin D	152840	-9.663	-10.196	-10.196	-28.127
Mollicellin U	-	-9.656	-9.701	-9.701	-42.702
Mollicellin J	24787300	-9.640	-9.646	-9.646	0.192
Connorstictic acid	-	-9.616	-9.641	-9.641	38.137
Cryptostictic acid	14991098	-9.597	-10.650	-10.650	4.946
Mollicellin V	-	-9.592	-10.043	-10.043	-57.454
Mollicellin H	153009	-9.565	-9.689	-9.689	-54.482
Simplicildone G	139590881	-9.554	-11.316	-11.316	-19.090

Title	PubChem CID/ChemSpider ID	docking score	XP GScore	glide gscore	glide emodel
Livistone B	-	-9.532	-9.566	-9.566	-33.287
Parmosidone A	132553220	-9.531	-10.434	-10.434	-23.852
Spiromastixone C	90670408	-9.517	-9.815	-9.815	-27.233
Spiromastixone J	86302535	-9.513	-9.529	-9.529	2.923
2,7-Dichlorounguinol	-	-9.501	-9.940	-9.940	35.351
Cryptostictic acid	14991098	-9.475	-10.529	-10.529	38.842
Purpactin A	10341722	-9.474	-9.503	-9.503	-43.037
Botryorhodine C= Botryosphaerone C	46916752	-9.471	-9.506	-9.506	-31.729
Mollicellin H	153009	-9.464	-10.492	-10.492	-31.126
Mollicellin Y	-	-9.451	-9.477	-9.477	-2.261
Lasiodiplodiaone B	-	-9.433	-11.289	-11.289	-10.717
Spiromastixone T	-	-9.425	-9.942	-9.942	6.717
Mollicellin G	129296	-9.421	-9.546	-9.546	-26.746
Simplicildone B	139590880	-9.420	-9.459	-9.459	-27.890
Protocetraric acid	5489486	-9.418	-10.321	-10.321	7.081
Garcinisidone A	10741092	-9.399	-11.061	-11.061	-33.803
3'-O-Demethylcryptostictinolide	-	-9.395	-9.445	-9.445	-49.684
Lasiodiplodiaone A	-	-9.392	-11.126	-11.126	-51.513
Garcinisidone F	8888728*	-9.389	-10.858	-10.858	-27.751
Simplicildone D	139590883	-9.385	-9.989	-9.989	19.684
Conhypoprotocetraric acid	71355957	-9.376	-9.387	-9.387	2.820
Connorstictic acid	-	-9.355	-9.380	-9.380	16.353
Spiromastixone C	90670408	-9.348	-9.965	-9.965	-45.438
Paucinervin Q	-	-9.327	-10.744	-10.744	-19.422
Folipastatin	6439424	-9.273	-9.303	-9.303	29.557
Mollicellin V	-	-9.262	-9.802	-9.802	-41.328
Simplicildone A	139590082	-9.262	-9.300	-9.300	-8.533
Botryorhodine F	139584782	-9.257	-9.374	-9.374	-36.757
Chaetosidone A = Corynesidone D	71816715	-9.242	-9.274	-9.274	-50.253
8'-O-methylprotocetraric acid	-	-9.239	-9.384	-9.384	-7.456
Norperistictic acid	101249643	-9.238	-10.190	-10.190	27.353
Simplicildone C	139590882	-9.198	-9.211	-9.211	8.896
Spiromastixone R1	-	-9.197	-9.809	-9.809	-0.543
Salazinic acid	5320418	-9.183	-10.340	-10.340	-3.412
Spiromastixone D	90670409	-9.156	-9.814	-9.814	-46.579
Dioxepin-11-one	487556	-9.153	-9.174	-9.174	26.395
Flavicansone	-	-9.143	-9.143	-9.143	-9.708
Hyperwightin A	-	-9.132	-9.178	-9.178	-52.755
Physodic acid	65751	-9.130	-11.018	-11.018	-20.451
Aspersidone	132820032	-9.107	-9.416	-9.416	0.566
Spiromastixone P	-	-9.100	-9.538	-9.538	-19.037
Curdepsidone G	146682941	-9.098	-9.130	-9.130	-2.470
Talaromyone A	156581493	-9.084	-9.084	-9.084	0.333
3'-O-Demethylcryptostictinolide	-	-9.059	-10.551	-10.551	-60.390
Aspergillusidone A	139291823	-9.033	-9.065	-9.065	-15.414
Spiromastixone P1	-	-9.031	-9.492	-9.492	-42.753

Title	PubChem CID/ChemSpider ID	docking score	XP GScore	glide gscore	glide emodel
Mollicellin W	-	-9.031	-9.948	-9.948	6.902
Aspergillusidone C	29214189*	-9.022	-9.296	-9.296	74.421
Spiromastixone D	90670409	-9.006	-9.708	-9.708	-49.121
Spiromastixone O	90670418	-8.990	-9.016	-9.016	2.985
2,4-Dichlorounguinol	29215420*	-8.983	-9.015	-9.015	18.844
11H-Dibenzo[b,e][1,4]dioxepin-4-carboxaldehyde,3,8-dihydroxy-9-methyl-1,6-bis[(1E)-1-methyl -1-propen-1-yl]-11-oxo	-	-8.975	-9.061	-9.061	225.869
Cordidepsine	-	-8.948	-9.810	-9.810	-19.383
Mollicellin G	129296	-8.948	-9.972	-9.972	1.597
Menegazziaic acid	71438918	-8.932	-11.493	-11.493	40.207
Botryorhodine A = Botryosphaerone A	46178006	-8.922	-9.055	-9.055	28.171
Mollicellin K	135565186	-8.916	-9.415	-9.415	36.681
Spiromastixone R	-	-8.906	-9.197	-9.197	-41.713
Mollicellin K	135565186	-8.896	-10.317	-10.317	-54.869
Conneotricone	-	-8.882	-10.140	-10.140	12.496
3,1`-Dichlorounguinol	-	-8.878	-9.153	-9.153	62.077
Spiromastixone S1	-	-8.876	-10.656	-10.656	-55.906
Aspergillusidone B	53468883	-8.874	-9.217	-9.217	26.830
Corynesidone A	42611455	-8.867	-8.929	-8.929	-51.094
Himantormione A	-	-8.866	-10.534	-10.534	-62.893
Garcinisidone A	10741092	-8.847	-9.626	-9.626	-49.784
(2RS)-Creticine	-	-8.835	-8.968	-8.968	-13.520
Asperunguissidone A	109107853*	-8.830	-10.452	-10.452	-8.641
Parmosidone B	132553221	-8.824	-10.050	-10.050	-33.195
Spiromastixone P	-	-8.818	-9.276	-9.276	-30.131
Spiromastixone Q	-	-8.806	-9.245	-9.245	-25.326
Parmosidone B	132553221	-8.802	-10.532	-10.532	-33.798
1-[(E)-but-2-en-2-yl]-2,8-dichloro-3,9-dihydroxy-4,7 -dimethylbenzo[b]-[1,4]- benzodioxepin-6-one	71494413	-8.781	-9.056	-9.056	11.300
Curdepsidone E	146682939	-8.777	-8.828	-8.828	78.314
Mollicellin C	50200	-8.773	-10.042	-10.042	25.002
Menegazziaic acid	71438918	-8.760	-8.905	-8.905	24.134
Spiromastixone A	90670406	-8.752	-10.421	-10.421	-29.743
7-Bromounguinol	146684023	-8.751	-9.033	-9.033	86.243
Vicanicin	324269	-8.718	-8.969	-8.969	52.162
Spiromastixone T	-	-8.710	-9.031	-9.031	-9.332
Garcinisidone F	8888728*	-8.708	-11.298	-11.298	-24.696
Botryorhodine A = Botryosphaerone A	46178006	-8.692	-9.724	-9.724	-43.100
Spiromastixone R1	-	-8.690	-9.101	-9.101	-17.179
Unguinol = Tridechloronornidulin	14131420	-8.667	-8.714	-8.714	27.332
Pilobolusone D	-	-8.666	-9.965	-9.965	11.448

Title	PubChem CID/ChemSpider ID	docking score	XP GScore	glide gscore	glide emodel
Spiromastixone Q1	-	-8.656	-9.098	-9.098	-24.788
Hypoprotocetraric acid	627044	-8.653	-8.664	-8.664	23.461
Menegazziaic acid	71438918	-8.641	-11.202	-11.202	5.012
Spiromastixone E	90670410	-8.630	-8.680	-8.680	-0.914
Asperunguissidone B	109107854*	-8.627	-8.887	-8.887	-12.623
Spiromastixone N	90670417	-8.620	-9.182	-9.182	1.692
Guanxidone A	-	-8.606	-8.664	-8.664	-14.661
Vicanicin	324269	-8.582	-9.002	-9.002	42.282
7-Bromounguinol	146684023	-8.577	-9.269	-9.269	17.166
Spiromastixone F	90670411	-8.574	-8.868	-8.868	-4.807
4-Methylunguinol	-	-8.561	-8.587	-8.587	16.608
Menegazziaic acid	71438918	-8.560	-8.753	-8.753	38.724
Spiromastixone I	90670414	-8.559	-8.588	-8.588	12.154
Botryorhodine B = Botryosphaerone B	46209444	-8.554	-8.677	-8.677	-35.161
Mollicellin L	44254337	-8.551	-9.516	-9.516	35.665
Mollicellin K	135565186	-8.533	-9.784	-9.784	-54.429
Maldoxone	10713846	-8.522	-8.654	-8.654	5.309
Guanxidone A	-	-8.521	-10.182	-10.182	9.383
Emeguisin D	-	-8.517	-9.047	-9.047	181.341
Spiromastixone S	-	-8.512	-8.541	-8.541	-4.384
Curdepsidone A	146684467	-8.509	-8.774	-8.774	15.588
Botryorhodine H	155518186	-8.506	-8.540	-8.540	-20.450
Excelsione = Phomopsidone	16109859	-8.503	-10.163	-10.163	6.772
Spiromastixone D	90670409	-8.478	-9.618	-9.618	-46.169
Aspersidone B	-	-8.473	-9.006	-9.006	9.219
Spiromastixone M	90670416	-8.428	-8.473	-8.473	-20.591
Spiromastixone H	90670413	-8.400	-8.991	-8.991	9.815
4,7-Dichlorounguinol	146684022	-8.400	-8.875	-8.875	3.421
Spiromastixone P	-	-8.399	-10.334	-10.334	-56.827
Spiromastixone D	90670409	-8.385	-9.287	-9.287	-33.451
Spiromastixone Q	-	-8.384	-8.842	-8.842	-23.924
Corynesidone B	78434980*	-8.378	-10.041	-10.041	-46.804
4,7-Dichlorounguinol	146684022	-8.373	-9.358	-9.358	92.518
Norstictic acid	5379540	-8.370	-8.774	-8.774	7.515
Norstictic acid	5379540	-8.369	-9.390	-9.390	9.364
Garcinisidone A	10741092	-8.367	-10.413	-10.413	-32.395
Spiromastixone Q1	-	-8.357	-8.817	-8.817	-20.627
Spiromastixone L	86302536	-8.351	-8.358	-8.358	9.740
Norcolensoic acid	71440531	-8.347	-10.140	-10.140	-43.483
Menegazziaic acid	71438918	-8.342	-9.245	-9.245	8.935
Simplicildone D	139590883	-8.327	-10.720	-10.720	21.718
Guanxidone B	-	-8.321	-8.358	-8.358	25.566
Boremexin C	156582387	-8.319	-9.439	-9.439	9.848
Parmosidone E	132553222	-8.318	-9.221	-9.221	-6.772
Spiromastixone F	90670411	-8.313	-8.905	-8.905	2.829
7-Bromofolipastatin	146684025	-8.298	-8.988	-8.988	26.242
Spiromastixone R	-	-8.287	-8.898	-8.898	-17.997
Spiromastixone B	90670407	-8.286	-8.564	-8.564	-22.258
2-Chlorounguinol	5387596	-8.286	-8.542	-8.542	-17.791

Title	PubChem CID/ChemSpider ID	docking score	XP GScore	glide gscore	glide emodel
Botryorhodine C = Botryosphaerone C	46916752	-8.284	-10.177	-10.177	27.892
Simplicildone G	139590881	-8.274	-10.726	-10.726	-14.317
Lasiodiplodiaone A	-	-8.242	-10.246	-10.246	-11.457
Salazinic acid	5320418	-8.240	-10.296	-10.296	8.544
Cetraric acid	5464155	-8.226	-9.129	-9.129	33.582
Mollicellin C	50200	-8.218	-9.877	-9.877	-10.787
Mollicellin I	24787299	-8.218	-10.502	-10.502	-30.368
Aspergillusidone E	29216360*	-8.209	-8.472	-8.472	-7.221
Aspergillusidone E	29216360*	-8.201	-8.835	-8.835	8.772
Simplicildone J	146683462	-8.179	-10.630	-10.630	1.254
Botryorhodine B = Botryosphaerone B	46209444	-8.161	-9.183	-9.183	-37.007
Nornidulin S5	-	-8.143	-8.159	-8.159	-4.413
Mollicellin O	146684100	-8.138	-9.930	-9.930	-54.534
Nornidulin S15	-	-8.117	-10.286	-10.286	54.850
2-Chlorounguinol	5387596	-8.111	-8.739	-8.739	89.998
Garcinisidone B	10526339	-8.105	-9.893	-9.893	-5.840
Pilobolusone A	-	-8.099	-9.567	-9.567	37.982
Asperunguissidone B	109107854*	-8.099	-8.729	-8.729	-14.394
Mollicellin I	24787299	-8.091	-9.981	-9.981	-33.014
Norcolensoic acid	71440531	-8.087	-8.116	-8.116	-64.468
Spiromastixone B	90670407	-8.070	-8.667	-8.667	-12.046
2,7-Dichlorounguinol	-	-8.020	-8.553	-8.553	10.962
Boremexin C	156582387	-8.014	-10.063	-10.063	17.586
Aspersidone	132820032	-8.005	-8.539	-8.539	33.091
Aspergillusidone A	139291823	-8.000	-9.731	-9.731	1.565
Chaetosidone A = Corynesidone D	71816715	-7.998	-9.752	-9.752	-43.699
Guanxidone B	-	-7.994	-9.868	-9.868	14.574
11H-Dibenzo[b,e][1,4]dioxepin-11-one,3,8-dihydroxy-4-(hydroxymethyl)-9-methyl-1,6-bis[(1E)-1-methyl-1-propen-1-yl]	-	-7.994	-9.556	-9.556	16.336
Aspergillusidone D	73350841	-7.992	-9.218	-9.218	8.105
3,8-Dihydroxy-4-(hydroxymethyl) -9-methyl-1,6-bis(1-methyl-1 -propenyl)- 11H-dibenzo [b,e][1,4]dioxepin-11-one	-	-7.978	-9.541	-9.541	14.942
Spiromastixone J	86302535	-7.950	-10.092	-10.092	11.747
Curdepsidone B	146682936	-7.940	-9.917	-9.917	-309.555
Nornidulin S15	-	-7.937	-10.106	-10.106	45.865
Vicanicin	324269	-7.932	-8.562	-8.562	24.604
Curdepsidone E	146682939	-7.922	-9.900	-9.900	-22.361
Mollicellin O	146684100	-7.903	-10.192	-10.192	-50.647
Botryorhodine E	122214817	-7.893	-9.129	-9.129	-44.637
2-Chloro-7-bromounguinol	146684024	-7.881	-8.172	-8.172	-19.480

Title	PubChem CID/ChemSpider ID	docking score	XP GScore	glide gscore	glide emodel
Vicanicin	324269	-7.878	-8.392	-8.392	20.743
Corynesidone C	60154238	-7.875	-9.419	-9.419	-49.707
Mollicellin W	-	-7.870	-8.012	-8.012	-0.661
Paucinervin Q	-	-7.841	-9.897	-9.897	-6.973
Mollicellin R	146684103	-7.822	-8.787	-8.787	26.698
Emeguisin B	6451094	-7.821	-8.163	-8.163	72.100
Aspergillusidone F	29216648*	-7.815	-8.251	-8.251	36.466
Botryorhodine I	156582113	-7.795	-10.346	-10.346	-33.379
Botryorhodine D = Botryosphaerone D	42637419	-7.790	-9.792	-9.792	-46.581
1H-Dibenzo[b,e][1,4]dioxepin-11-one,3,8-dihydroxy-4-(methoxymethyl)-1,6-dimethyl	-	-7.790	-9.792	-9.792	-46.462
Livistone A	-	-7.789	-9.791	-9.791	-44.704
11H-Dibenzo[b,e][1,4]dioxepin-11-one,3,8-dihydroxy-4-(methoxymethyl)-9-methyl-1,6-bis[(1E)-1-methyl-1-propen-1-yl	-	-7.777	-9.277	-9.277	74.020
Cordidepsine	-	-7.772	-8.001	-8.001	-5.271
Curdepsidone G	146682941	-7.772	-9.530	-9.530	-41.421
Variolaric acid	12444681	-7.762	-9.842	-9.842	-60.143
11H-Dibenzo[b,e][1,4]dioxepin -11-one,3,8-dihydroxy-4-(hydroxymethyl)-9-methyl-1,6-bis[(1E)-1-methyl-1-propen-1-yl]	-	-7.748	-10.171	-10.171	42.286
Botryorhodine D = Botryosphaerone D	42637419	-7.714	-9.484	-9.484	-39.107
Botryorhodine F	139584782	-7.699	-8.925	-8.925	12.536
Curdepsidone F	146682940	-7.696	-9.340	-9.340	-54.456
Polyanthadepsidone A	-	-7.692	-9.417	-9.417	-8.574
Lasiodiplodiaone B	-	-7.689	-10.137	-10.137	-17.161
Aspergillusidone F	29216648*	-7.686	-8.582	-8.582	6.624
Aspergillusidone F	29216648*	-7.669	-8.888	-8.888	3.612
Spiromastixone Q1	-	-7.664	-9.552	-9.552	-11.340
Himantormione B	-	-7.655	-9.336	-9.336	-47.630
1H-Dibenzo[b,e][1,4]dioxepin-11 -one,3,8-dihydroxy-4-(methoxymethyl)-1,6-dimethyl	-	-7.643	-9.413	-9.413	-40.741
Menegazziaic acid	71438918	-7.632	-9.824	-9.824	-12.765
Cordycepsidone A = Excelsional	57382387	-7.581	-10.007	-10.007	-1.518
Curdepsidone A	146684467	-7.579	-8.424	-8.424	15.556
Curdepsidone C	146682937	-7.550	-9.526	-9.526	-204.873
Purpactin A	10341722	-7.543	-9.358	-9.358	15.603

Title	PubChem CID/ChemSpider ID	docking score	XP GScore	glide gscore	glide emodel
Corynesidone E	132512651	-7.540	-9.242	-9.242	-16.457
3,8-Dihydroxy-4-(hydroxymethyl) -9-methyl-1,6-bis(1-methyl-1-propenyl)- 11H-dibenzo[b,e][1,4] dioxepin-11-one	-	-7.534	-9.956	-9.956	37.479
4,7-Dichlorounguinol	146684022	-7.524	-8.662	-8.662	17.151
Curdepsidone D	146682938	-7.522	-9.362	-9.362	21.955
Botryorhodine E	122214817	-7.498	-9.238	-9.238	-44.014
Botryorhodine H	155518186	-7.498	-9.481	-9.481	-7.974
Spiromastixone M	90670416	-7.493	-9.255	-9.255	-26.328
3,1`-Dichlorounguinol	-	-7.490	-8.275	-8.275	-32.738
Aspergillusidone C	29214189*	-7.480	-8.264	-8.264	-2.146
2,7-Dichlorounguinol	-	-7.479	-9.004	-9.004	21.987
Spiromastixone N	90670417	-7.477	-9.355	-9.355	-26.572
11H-Dibenzo[b,e][1,4]dioxepin-4-carboxaldehyde,3,8-dihydroxy-9-methyl-1,6-bis[(1E)-1-methyl-1-propen-1-yl]-11-oxo	-	-7.454	-8.648	-8.648	55.455
Pilobolusone A	-	-7.430	-9.861	-9.861	30.642
2-Chloro-7-bromounguinol	146684024	-7.423	-8.174	-8.174	6.292
Variolaric acid	12444681	-7.421	-7.461	-7.461	3.798
11H-Dibenzo[b,e][1,4]dioxepin-11-one,3,8-dihydroxy-4-(methoxymethyl)-9-methyl-1,6-bis[(1E)-1-methyl-1-propen-1-yl	-	-7.395	-9.822	-9.822	37.431
Polyanthadepsidone A	-	-7.391	-9.015	-9.015	-14.712
Nornidulin S5	-	-7.376	-9.545	-9.545	33.347
Mollicellin U	-	-7.371	-9.133	-9.133	-52.942
Corynesidone B	78434980*	-7.367	-9.676	-9.676	12.995
Botryorhodine G	122214818	-7.366	-9.372	-9.372	-39.311
4-Formyl-3,8-dihydroxy-9-methyl-1,6-bis(1-methyl-1-propenyl)-11H-dibenzo[b,e][1,4]dioxepin-11-one	-	-7.362	-8.557	-8.557	50.563
Mollicellin J	24787300	-7.361	-10.060	-10.060	-7.015
1-[(E)-but-2-en-2-yl]-2,8-dichloro-3,9-dihydroxy-4,7-dimethylbenzo[b]-[1,4]-benzodioxepin-6-one	71494413	-7.353	-8.138	-8.138	8.129
Mollicellin V	-	-7.340	-8.868	-8.868	20.236
Curdepsidone D	146682938	-7.329	-9.307	-9.307	-37.279
Spiromastixone R1	-	-7.322	-9.168	-9.168	-11.974
Botryorhodine E	122214817	-7.278	-9.724	-9.724	-39.310
Himantormione A	-	-7.275	-9.295	-9.295	-44.084

Title	PubChem CID/ChemSpider ID	docking score	XP GScore	glide gscore	glide emodel
3,8-Dihydroxy-4-(methoxymethyl) -9-methyl-1,6-bis(1-methyl-1-propenyl)-11Hdibenzo[b,e][1,4] dioxepin-11-one	-	-7.272	-9.699	-9.699	35.296
Nidulin	6450195	-7.263	-7.279	-7.279	83.965
Spiromastixone S	-	-7.252	-9.530	-9.530	-12.052
Connorstictic acid	-	-7.246	-9.129	-9.129	54.740
Spiromastixone N	90670417	-7.238	-9.608	-9.608	9.870
Aspergillusidone D	73350841	-7.230	-8.124	-8.124	8.829
Protocetraric acid	5489486	-7.222	-7.367	-7.367	3.439
Corynesidone A	42611455	-7.210	-8.854	-8.854	-49.897
Livistone B	-	-7.164	-9.057	-9.057	-32.734
Spiromastixone G	90670412	-7.129	-7.145	-7.145	14.297
Livistone A	-	-7.100	-8.870	-8.870	-44.984
Mollicellin U	-	-7.067	-9.334	-9.334	-50.245
Simplicildone B	139590880	-7.052	-9.504	-9.504	-18.176
Spiromastixone C	90670408	-7.032	-9.302	-9.302	-45.437
Diaporthol B	139591243	-7.026	-9.399	-9.399	78.929
Unguinol = Tridechloronornidulin	14131420	-7.022	-8.768	-8.768	-6.944
Conhypoprotocetraric acid	71355957	-7.019	-9.357	-9.357	-6.582
Spiromastixone Q	-	-7.009	-8.944	-8.944	-29.095
Spiromastixone O	90670418	-6.998	-9.025	-9.025	2.889
Spiromastixone P1	-	-6.990	-8.878	-8.878	-33.427
Botryorhodine F	139584782	-6.989	-8.720	-8.720	-31.928
Spiromastixone A	90670406	-6.983	-8.990	-8.990	-31.929
Spiromastixone C	90670408	-6.974	-9.283	-9.283	-43.040
Emeguisin D	-	-6.964	-8.580	-8.580	47.373
1-[(E)-but-2-en-2-yl]-2,8-dichloro -3,9-dihydroxy-4,7-dimethylbenzo[b]-[1,4]-benzodioxepin-6-one	1494413	-6.962	-8.605	-8.605	8.210
Aspergillusidone C	29214189*	-6.961	-8.604	-8.604	8.590
Spiromastixone P1	-	-6.958	-9.148	-9.148	-48.638
Unguinol = Tridechloronornidulin	14131420	-6.899	-9.143	-9.143	36.087
Simpliciildone A	139590082	-6.891	-9.341	-9.341	-2.806
Spiromastixone G	90670412	-6.884	-9.026	-9.026	3.785
Simpliciildone A	139590082	-6.861	-8.689	-8.689	-34.227
Corynesidone C	60154238	-6.861	-8.836	-8.836	-25.724
Nidulin	6450195	-6.860	-9.029	-9.029	38.361
Spiromastixone R	-	-6.845	-9.318	-9.318	-46.124
Spiromastixone M	90670416	-6.840	-9.157	-9.157	-20.965
Curdepsidone A	146684467	-6.838	-9.236	-9.236	-21.757
Botryorhodine G	122214818	-6.837	-8.542	-8.542	-46.360
Spiromastixone E	90670410	-6.835	-8.518	-8.518	-14.751
Folipastatin	6439424	-6.834	-9.090	-9.090	24.599
Curdepsidone F	146682940	-6.828	-8.817	-8.817	-28.549

Title	PubChem CID/ChemSpider ID	docking score	XP GScore	glide gscore	glide emodel
Curdepsidone C	146682937	-6.824	-8.697	-8.697	19.832
Asperunguissidone A	109107853*	-6.818	-8.786	-8.786	-9.215
7-Bromounguinol	146684023	-6.813	-8.563	-8.563	9.088
Curdepsidone B	146682936	-6.806	-8.679	-8.679	38.495
Spiromastixone R	-	-6.784	-9.219	-9.219	-40.867
2,4-Dichlorounguinol	29215420*	-6.779	-9.111	-9.111	31.956
Spiromastixone E	90670410	-6.765	-9.064	-9.064	3.243
Spiromastixone Q1	-	-6.744	-8.933	-8.933	-26.068
Spiromastixone I	90670414	-6.737	-9.037	-9.037	-2.057
2-Chloro-7-bromounguinol	146684024	-6.730	-8.568	-8.568	37.436
Corynesidone A	42611455	-6.686	-8.668	-8.668	-39.084
Livistone B	-	-6.623	-9.070	-9.070	-11.805
Spiromastixone Q	-	-6.613	-8.849	-8.849	-34.813
Spiromastixone P	-	-6.602	-8.838	-8.838	-49.982
Botryorhodine C = Botryosphaerone C	46916752	-6.590	-9.037	-9.037	-11.569
2,7-Dichlorounguinol	-	-6.548	-8.449	-8.449	-22.176
4-Methylunguinol	-	-6.543	-8.871	-8.871	13.339
Spiromastixone R1	-	-6.452	-7.814	-7.814	-22.862
Spiromastixone H	90670413	-6.440	-8.864	-8.864	0.858
Simplicildone B	139590880	-6.424	-8.219	-8.219	-26.183
1-[(E)-but-2-en-2-yl]-2,8-dichloro -3,9-dihydroxy-4,7-dimethylbenzo [b]-[1,4]- benzodioxepin-6-one	1494413	-6.408	-8.279	-8.279	30.702
Mollicellin Q	146684102	-6.395	-8.107	-8.107	-12.046
4-Methylunguinol	-	-6.389	-8.641	-8.641	12.390
Aspergillusidone C	29214189*	-6.358	-8.229	-8.229	29.854
3,1`-Dichlorounguinol	-	-6.358	-8.229	-8.229	29.854
Spiromastixone K	90670415	-6.278	-8.478	-8.478	6.484
Gangaleoidin	4565183	-6.257	-8.426	-8.426	1.636
Spiromastixone L	86302536	-6.246	-8.888	-8.888	7.757
Garcinisidone A	10741092	-6.217	-8.764	-8.764	-55.441
Spiromastixone F	90670411	-6.210	-8.634	-8.634	-1.560
Dioxepin-11-one	487556	-6.169	-8.517	-8.517	-26.080
Vicanicin	324269	-6.089	-8.157	-8.157	19.001
Mollicellin X	-	-6.014	-8.725	-8.725	40.746
Corynesidone C	60154238	-6.009	-8.388	-8.388	-15.913
Corynesidone E	132512651	-6.007	-8.423	-8.423	-14.100
Excelsione = Phomopsidone	16109859	-6.001	-6.057	-6.057	-25.837
Nornidulin = Ustin	20056625	-5.993	-8.622	-8.622	14.310
7-Bromounguinol	146684023	-5.993	-8.435	-8.435	14.208
Spiromastixone O	90670418	-5.919	-8.580	-8.580	10.939
Curdepsidone A	146684467	-5.892	-7.327	-7.327	-27.977
Curdepsidone C	146682937	-5.845	-5.894	-5.894	4.381
Guanxidone B	-	-5.725	-8.083	-8.083	0.000
Dioxepin-11-one	487556	-5.620	-8.054	-8.054	-8.220
3-Hydroxyphysodic acid	171308	-5.566	-7.430	-7.430	-29.363
Spiromastixone I	90670414	-5.432	-7.546	-7.546	15.128

Title	PubChem CID/ChemSpider ID	docking score	XP GScore	glide gscore	glide emodel
8'-O-methylprotocetraric acid	-	-5.281	-6.184	-6.184	34.308
Purpactin A	10341722	-5.101	-5.129	-5.129	1.194
Botryorhodine I	156582113	-5.013	-5.246	-5.246	-37.685
Siphulellic acid	-	-4.861	-5.928	-5.928	25.759
Botryorhodine A = Botryosphaerone A	46178006	-4.841	-7.177	-7.177	-8.247
Himantormione B	-	-4.753	-6.772	-6.772	-33.138
3,8-Dihydroxy-4-(methoxymethyl) -9-methyl-1,6-bis(1-methyl-1-propenyl)-11Hdibenzo[b,e][1,4] dioxepin-11-one	-	-4.683	-6.183	-6.183	126.318
Curdepsidone B	146682936	-4.644	-4.693	-4.693	13.217
3,1`-Dichlorounguinol	-	-4.588	-6.231	-6.231	13.251
Parmosidone K	-	-4.442	-6.872	-6.872	116.281
Simplicildone D	139590883	-4.368	-4.650	-4.650	17.466
Nornidulin S25	-	-4.318	-6.629	-6.629	66.905
Cordycepsidone A = Excelsional	57382387	-4.168	-4.942	-4.942	24.865
Psoromic acid	23725	-4.094	-5.011	-5.011	-466.169
Gangaleoidin	4565183	-3.954	-3.970	-3.970	-16.329
Cordidepsine	-	-3.931	-6.113	-6.113	-27.148
Auranticin A	6439369	-3.714	-3.731	-3.731	-112.503
Simplicildone J	146683462	-3.612	-5.400	-5.400	19.016
Simplicildone E	139590884	-3.391	-3.780	-3.780	21.459
Variolaric acid	12444681	-3.346	-5.336	-5.336	0.000
Emeguisin D	-	-3.221	-5.027	-5.027	33.650
(2RS)-Creticine	-	-3.050	-4.227	-4.227	127.568
Nornidulin S6	-	-3.018	-3.034	-3.034	247.503
Psoromic acid	23725	-2.864	-3.781	-3.781	7.956
Mollicellin P	146684101	-2.618	-4.330	-4.330	41.470
Neotricone	101249642	-2.545	-3.414	-3.414	35.939
Nornidulin S19	-	-2.460	-4.629	-4.629	34.278
Parmosidone E	132553222	-2.430	-2.575	-2.575	20.675
Botryorhodine H	155518186	-2.313	-4.613	-4.613	-34.340
Boremexin A	156582385	-2.238	-3.996	-3.996	19.213
Boremexin B	156582386	-2.238	-3.996	-3.996	19.213
Nornidulin = Ustin	20056625	-2.196	-2.574	-2.574	20.855
(2RS)-Creticine	-	-2.172	-4.748	-4.748	81.552
Parmosidone A	132553220	-1.986	-2.131	-2.131	15.109
Nornidulin S16	-	-1.879	-4.047	-4.047	37.714
Nornidulin S6	-	-1.758	-3.927	-3.927	32.986
7-Chlorofolipastatin = Emeguisin A	14164492	-1.673	-3.764	-3.764	33.707
Hypoprotocetraric acid	627044	-1.465	-3.803	-3.803	18.577
Pilobolusone B	-	-1.416	-3.684	-3.684	58.962
Folipastatin	6439424	-1.322	-3.469	-3.469	19.026
Excelsione = Phomopsidone	16109859	-1.318	-3.459	-3.459	-30.007
Vicanicin	324269	-0.924	-2.616	-2.616	3.540

Title	PubChem CID/ChemSpider ID	docking score	XP GScore	glide gscore	glide emodel
4,7-Dichlorounguinol	146684022	-0.920	-1.829	-1.829	45.086
Curdepsidone E	146682939	-0.817	-2.657	-2.657	-120.266
3-Hydroxyphysodic acid	171308	-0.373	-1.591	-1.591	-7.839
Pilobolusone B	-	0.555	-1.254	-1.254	20.757
Physodalic acid	5489369	0.612	-0.291	-0.291	38.322
Physodalic acid	5489369	2.200	2.054	2.054	30.971
Nornidulin S20	-	3.189	1.020	1.020	34.977

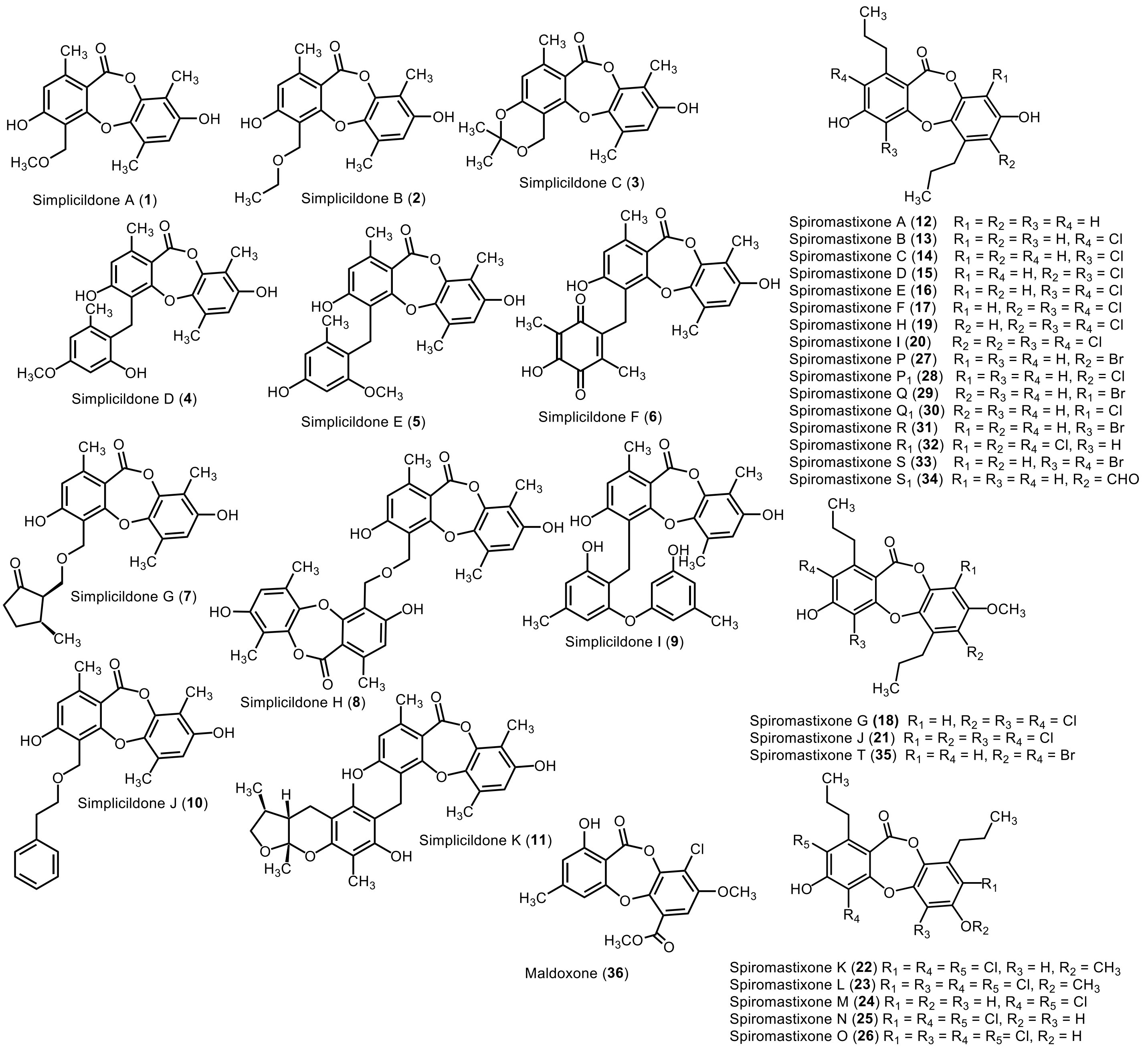

Figure S1. Chemical structures of depsidones (**1–36**).

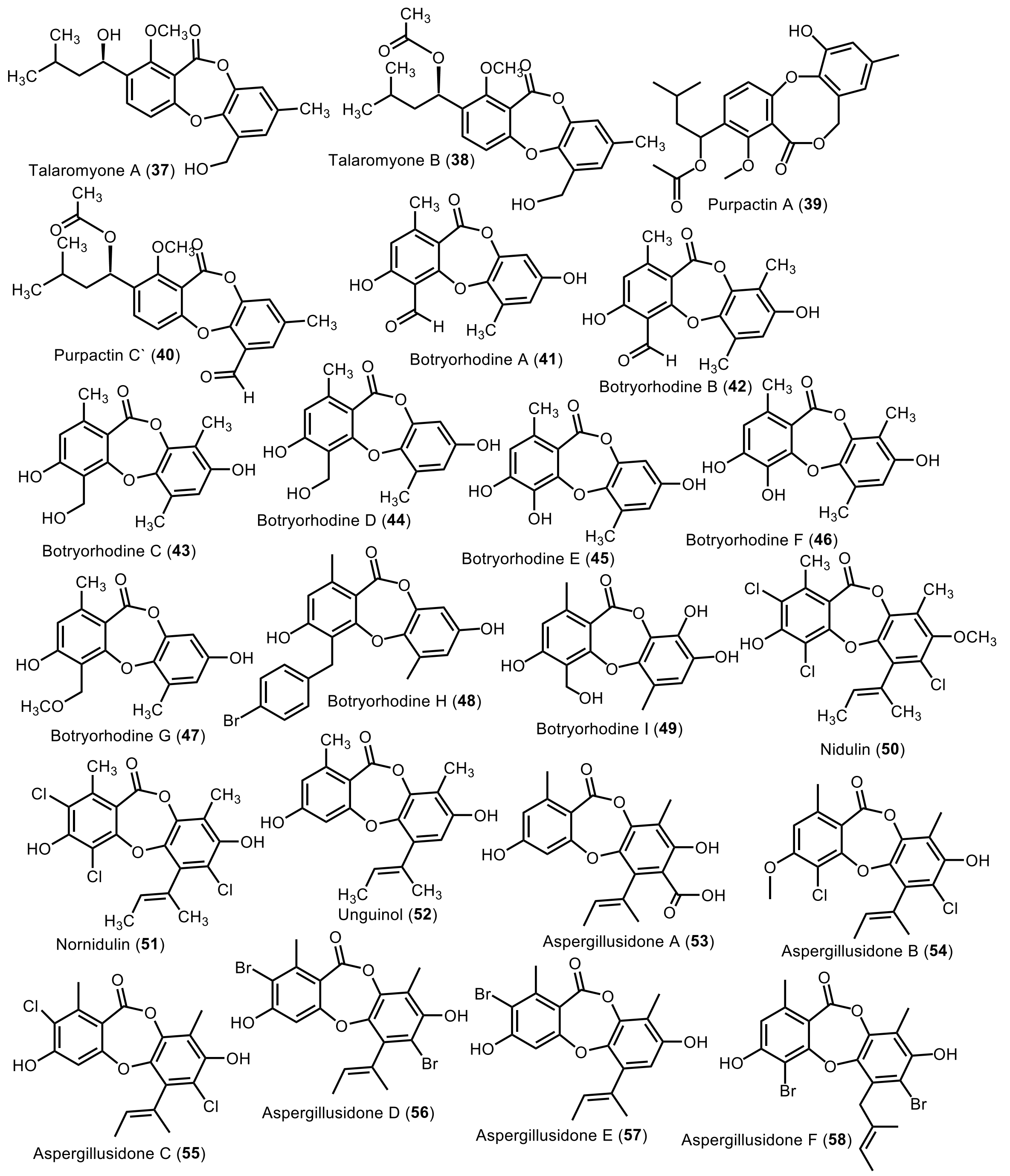

Figure S2. Chemical structures of depsidones (**37–58**).

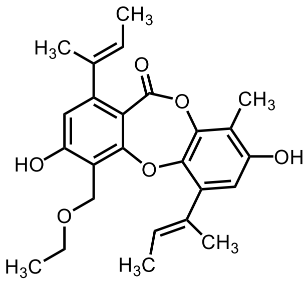

Pilobolusone A (**61**)

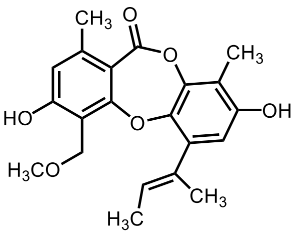

Pilobolusone B (**62**)

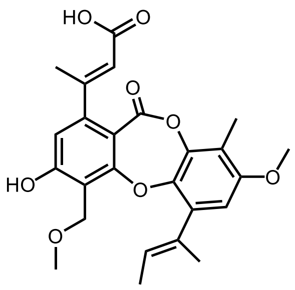

Pilobolusone C (**63**)

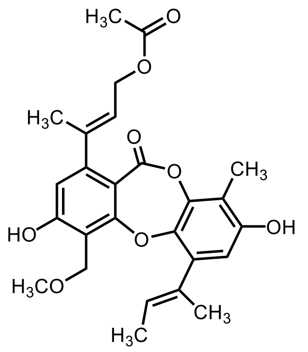

Pilobolusone D (**64**)

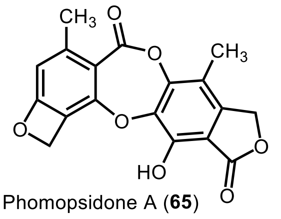

Phomopsidone A (**65**)

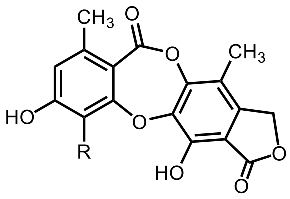

Excelsione (**66**) R = CH2OH
Cordycepsidone A (**67**) R = CHO
Cordycepsidone B (**68**) R = COOH

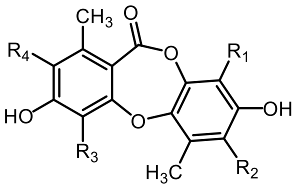

Corynesidone A (**69**) $R_1 = R_2 = R_3 = R_4$ = H
Corynesidone B (**70**) R1 = R3 = H, R2 = COOH, R4 = OH
Corynesidone C (**71**) R1 = R2 = R3 = H, R4 = OH
Corynesidone D (**72**) R1 = R3 = R4 = H, R4 = COOH
Corynesidone E (**73**) R1 = R2 = R3 = H, R4 = OH

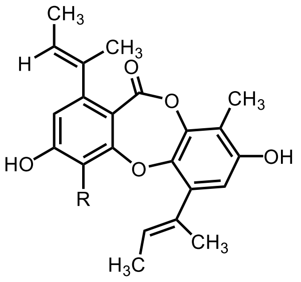

11H-Dibenzo[b,e][1,4]dioxepin-4-carboxaldehyde, 3,8-dihydroxy- 9-methyl-1,6-bis[(1E)-1-methyl-1-propen-1-yl]-11-oxo (**74**) R= CHO
11H-Dibenzo[b,e][1,4]dioxepin-11-one,3,8-dihydroxy-4-(hydroxymethyl)-9-methyl-1,6-bis[(1E)-1-methyl-1-propen-1-yl] (**75**) R= CH2OH
11H-Dibenzo[b,e][1,4]dioxepin-11-one,3,8-dihydroxy-4-(methoxymethyl)-9-methyl-1,6-bis[(1E)-1-methyl-1-propen-1-yl (**76**) R= CH2OCH3
Folipastatin (**77**) R= CH3

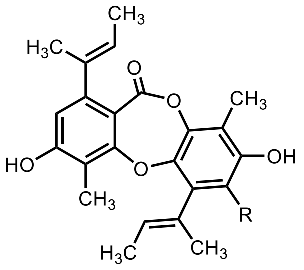

7-Chlorofolipastatin (**78**) R = Cl
7-Bromofolipastatin (**79**) R = Br
7-Carboxyfolipastatin (**80**) R = COOH

Figure S3. Chemical structures of depsidones (**59–80**).

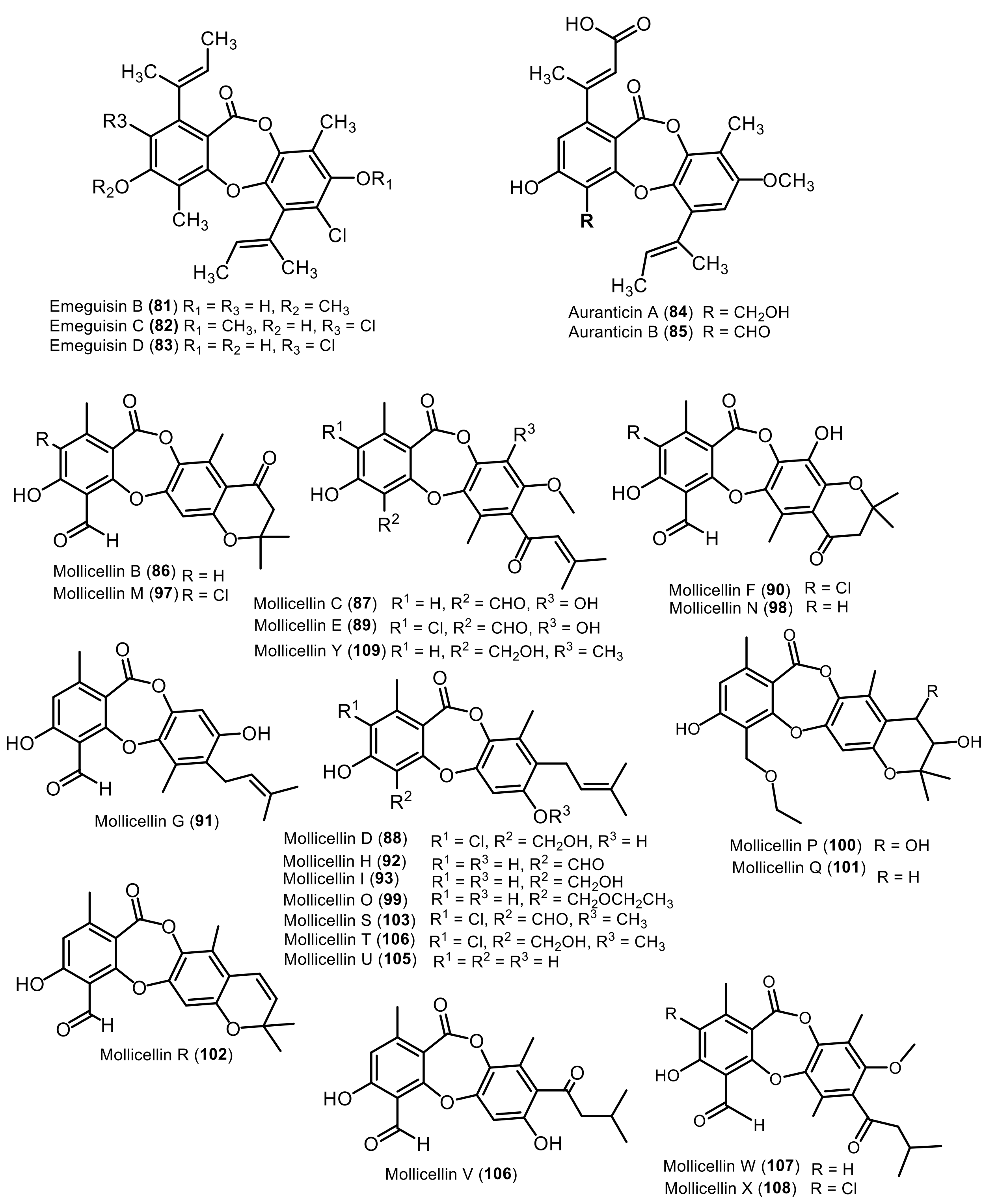

Figure S4. Chemical structures of depsidones (**81–109**).

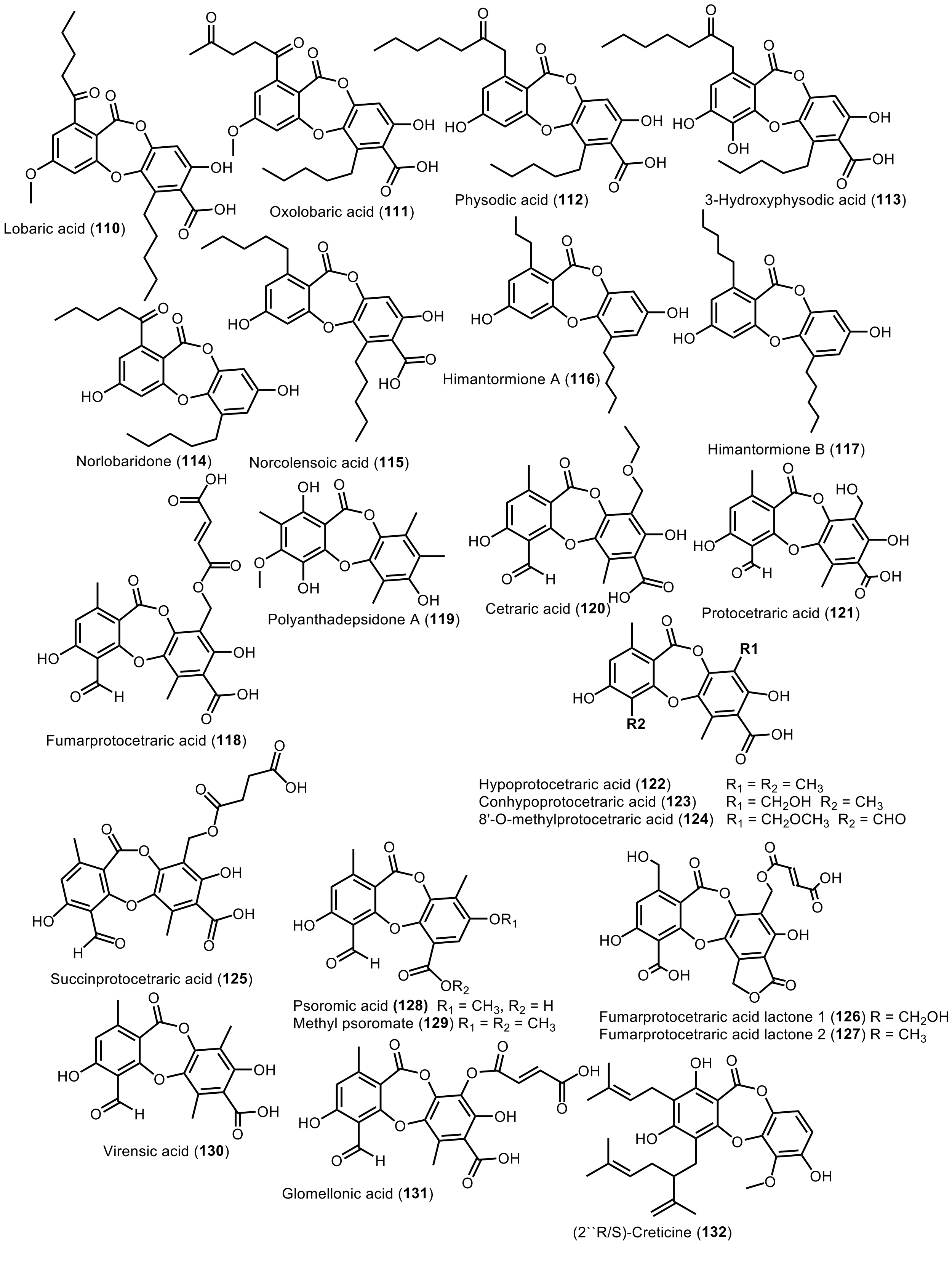

Figure S5. Chemical structures of depsidones (**110–132**).

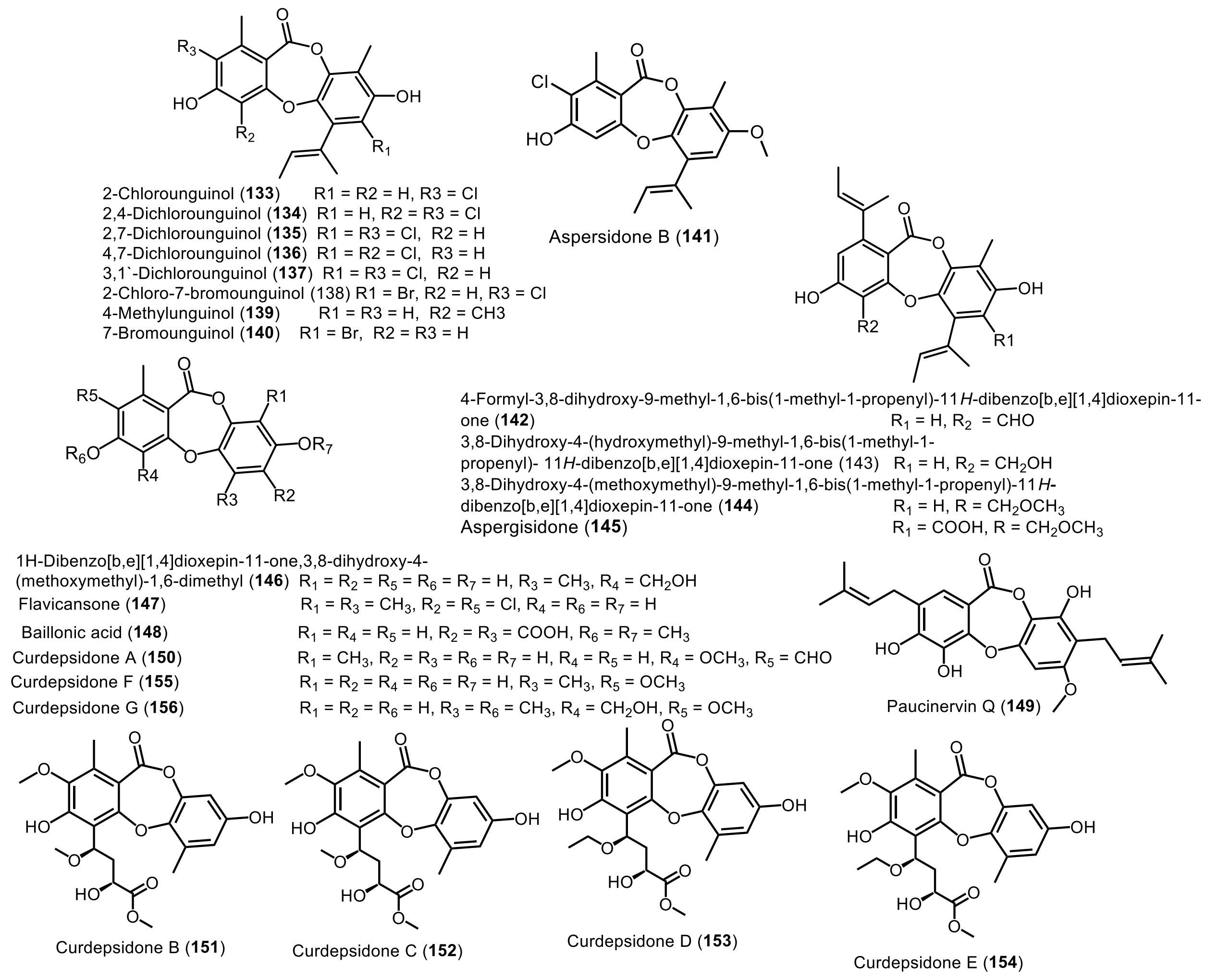

Figure S6. Chemical structures of depsidones (**133–156**).

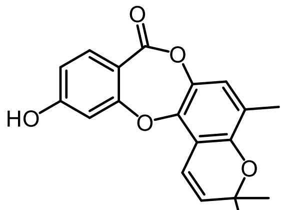

Hyperwightin A (**157**)

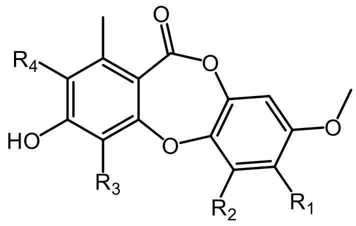

Cordidepsine (**158**) R1 = CH3 R2 = OH R3 = CHO, R4 = H
Gangaleoidin (**162**) R1 = COCH3 R2 = CH3 R3 = R4 = Cl

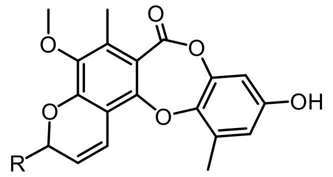

Boremexin A (**159**) R = α-$COCH_3$
Boremexin B (**160**) R = β-$COCH_3$

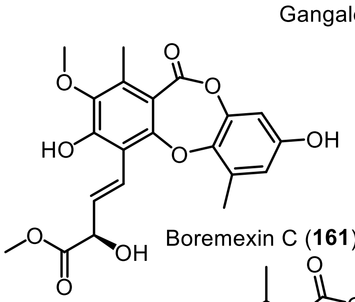

Boremexin C (**161**)

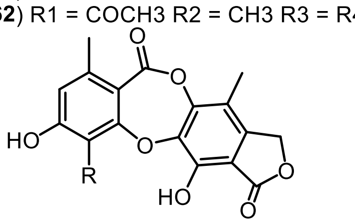

Guanxidone A (**163**) R = CH_2OCH_3
Guanxidone B (**164**) R = CH_3

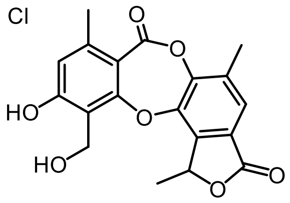

Connorstictic acid (**165**)

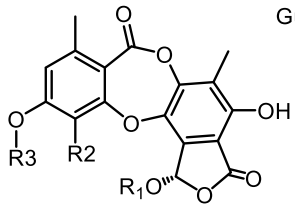

Ceratinalone (**166**) R1 = CH2CH3, R2 = OH, R3 = CH3
Stictic acid (**167**) R1 = H, R2 = CHO, R3 = CH3
Norstictic acid (**168**) R1 = R3 = H, R2 = CHO
8\`-O-Methylstictic acid (**169**) R1 = R3 = CH3, R2 = CHO
8\`-O-Ethylstictic acid (**170**) R1 = CH2CH3, R3 = CH3, R2 = CHO

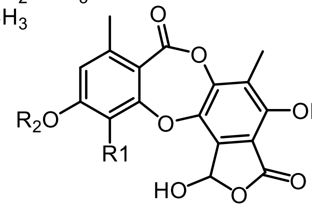

Norperististic acid (**171**) R1 = COOH, R2 = H
Cryptostictic acid (**172**) R1 = CH2OH, R2 = CH3
Peristictic acid (**173**) R1 = COOH, R2 = CH3

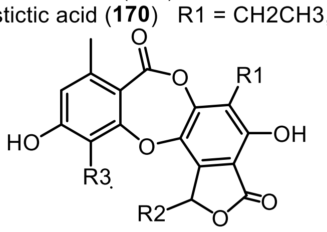

Neotricone (**174**) $R_1 = CH_3$, R_2 = H, R_3 = COOH
Conneotricone (**175**) $R_1 = CH_2OH$, R_2 = H, R_3 = COOH
Salazinic acid (**176**) $R_1 = CH_2OH$, R_2 = α-OH, R_3 = CHO
Perisalazinic acid (**177**) $R_1 = CH_2OH$, R_2 = α-OH, R_3 = COOH
8'-O-Methylsalazinic acid (**178**) $R_1 = CH_2OCH_3$, R_2 = OH, R_3 = CHO
Menegazziaic acid (**179**) $R_1 = CH_3$, R_2 = OH, $R_3 = CH_2OH$

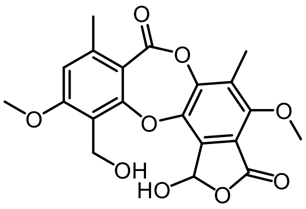

1-Hydroxy-11-(hydroxymethyl)-4,10-dimethoxy-5,8-dimethyl-7H-benzo[6,7][1,4]dioxepino [2,3-e]isobenzofuran-3,7(1H)-dione (**180**)

Figure S7. Chemical structures of depsidones (**157–180**).

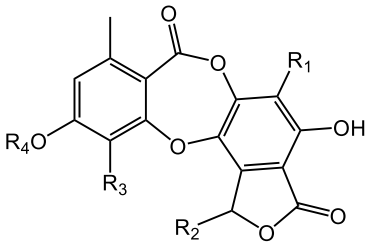

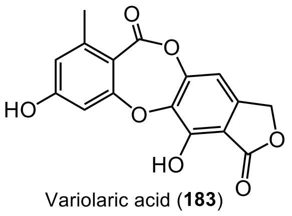

Variolaric acid (**183**)

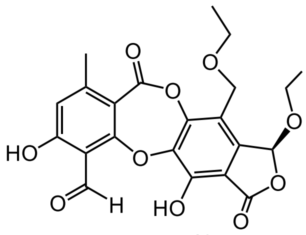

Diffractione B (**185**)

Bailesidone (**181**) $R_1 = R_4 = CH_3$, R_2 = α-CH_2COCH_3, R_3 = OH
3'-O-Demethylcryptostictinolide (**182**) $R_1 = R_2$ = H, $R_3 = CH_2OH$, $R_4 = CH_3$
Galbinic acid (**184**) $R_1 = CH_2OCOCH_3$, R_2 = OH, R_3 = CHO, R_4 = H
Salazinin A (**186**) $R_1 = CH_2OCH_2CH_3$, R_2 = β-OH, R_3 = CHO, R_4 = H

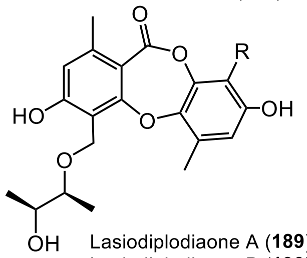

Lasiodiplodiaone A (**189**) R = H
Lasiodiplodiaone B (**190**) R = CH_3

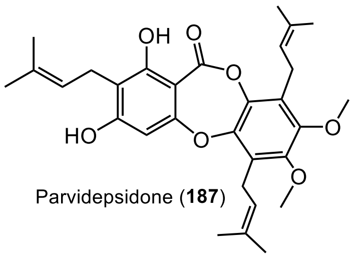

Parvidepsidone (**187**)

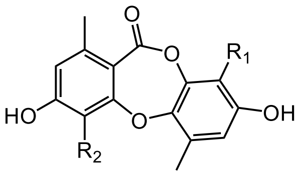

Dioxepin-11-one (**188**) $R_1 = R_2 = CH_3$
Livistone A (**191**) R_1 = H, $R_2 = CH_2OH$
Livistone B (**192**) $R_1 = CH_3$, $R_2 = CH_2OH$

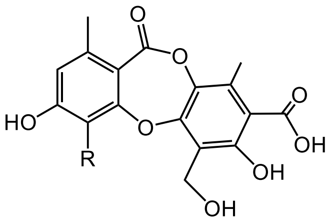

Parmosidone A (**193**) R= CHO
Parmosidone B (**194**) R = OH

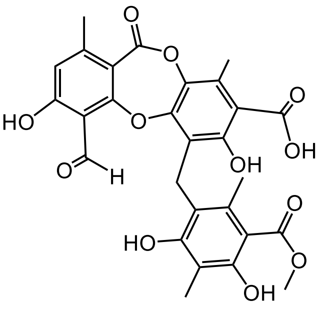

Parmosidone C (**195**) R = CH_3
Parmosidone D (**196**) R = H

Figure S8. Chemical structures of depsidones (**181–196**).

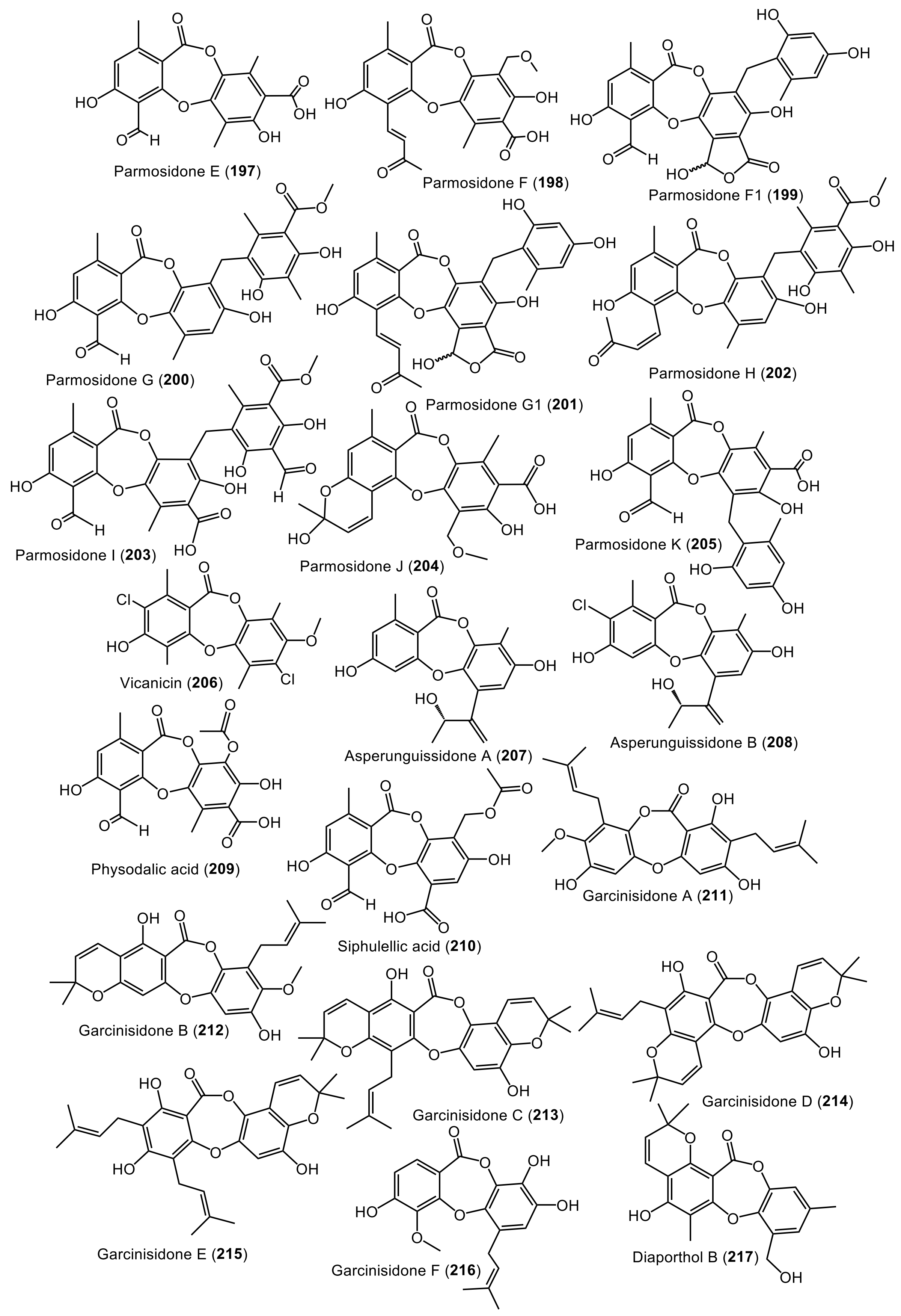

Figure S9. Chemical structures of depsidones (**197–217**).

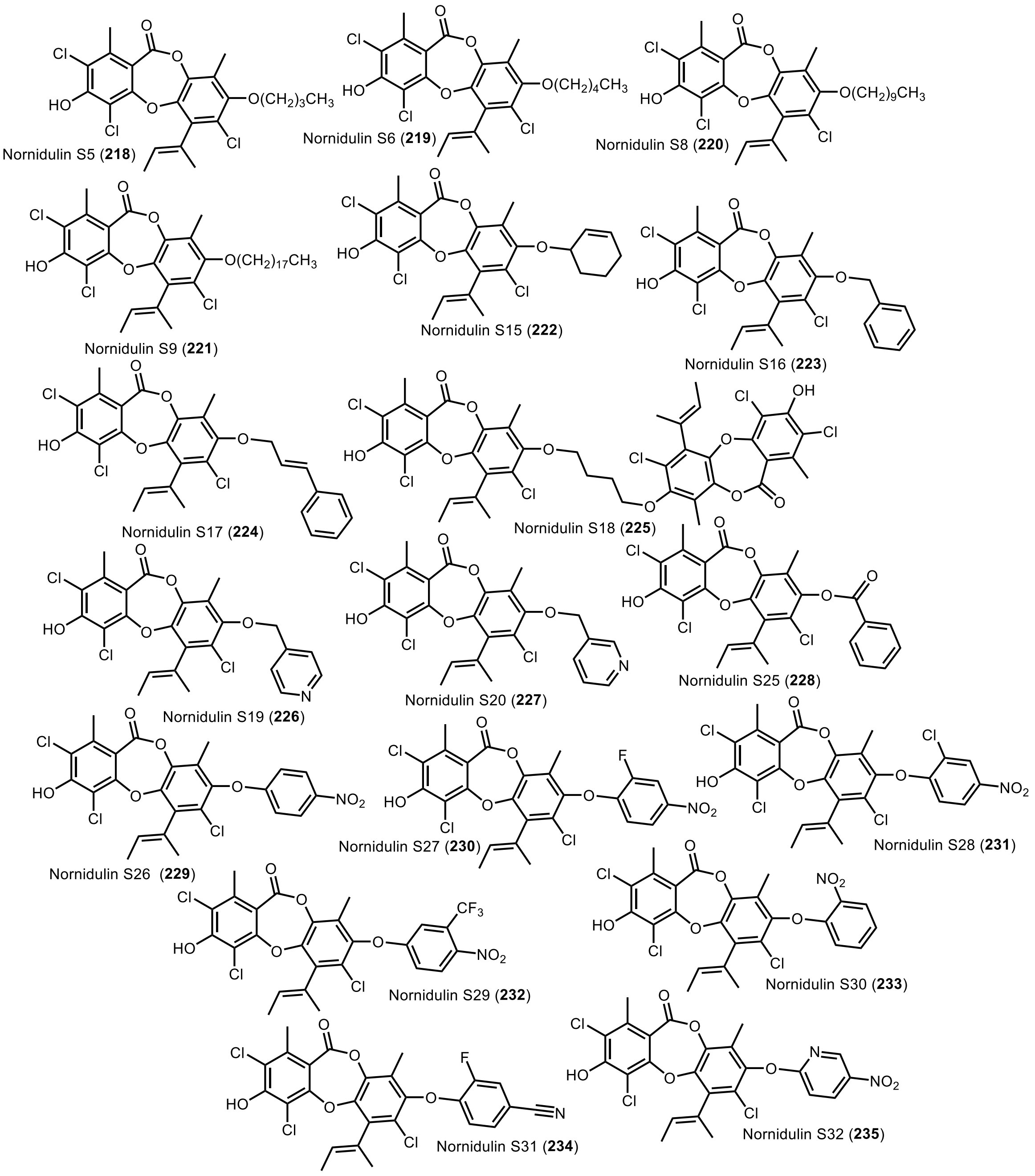

Figure S10. Chemical structures of nornidulin semisynthetic derivatives (**218–235**). *These derivatives have been named relative to nornidulin.

Simulation Interactions Diagram Report

Simulation Details

Jobname: desmond_md_job_6KPF_ref
Entry title: 6KPF_ref - prepared

CPU #	Job Type	Ensemble	Temp. [K]	Sim. Time [ns]	# Atoms	# Waters	Charge
1	mdsim	NPT	300.0	100.102	162404	48141	0

Protein Information

Tot. Residues	Prot. Chain(s)	Res. in Chain(s)	# Atoms	# Heavy Atoms	Charge
1135	'A', 'B', 'C', 'R', 'S'	[illegible]ict_values([218, 338, 57, 290, [illegible]])	17243	8842	+4

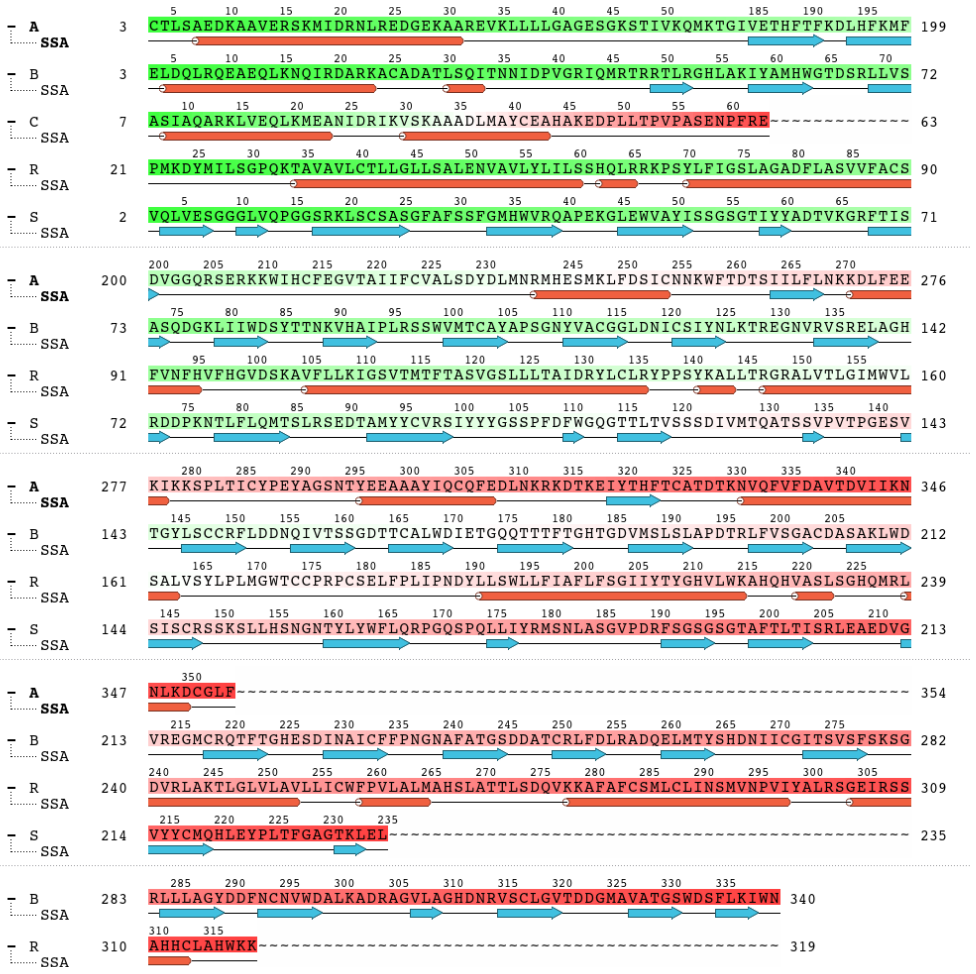

Ligand Information

SMILES	N#CCCCCCC(C)(C)c(cc1O)cc(c12)OC(C)(C)[C@H]3[C@H]2C[C@H](CO)CC3
PDB Name	'UNK'
Num. of Atoms	66 (total) 29 (heavy)
Atomic Mass	399.579 au
Charge	0
Mol. Formula	C25H37NO3
Num. of Fragments	1
Num. of Rot. Bonds	9

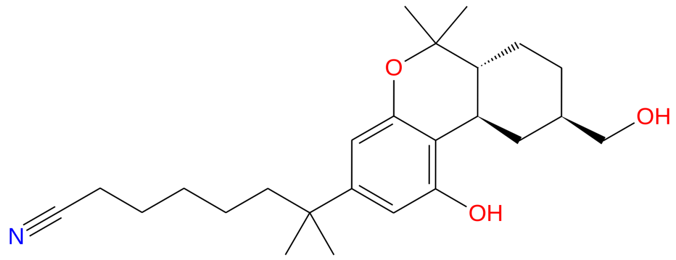

Counter Ion/Salt Information

Type	Num.	Concentration [mM]	Total Charge
Cl	138	52.120	-138
Na	134	50.609	+134

Protein-Ligand RMSD

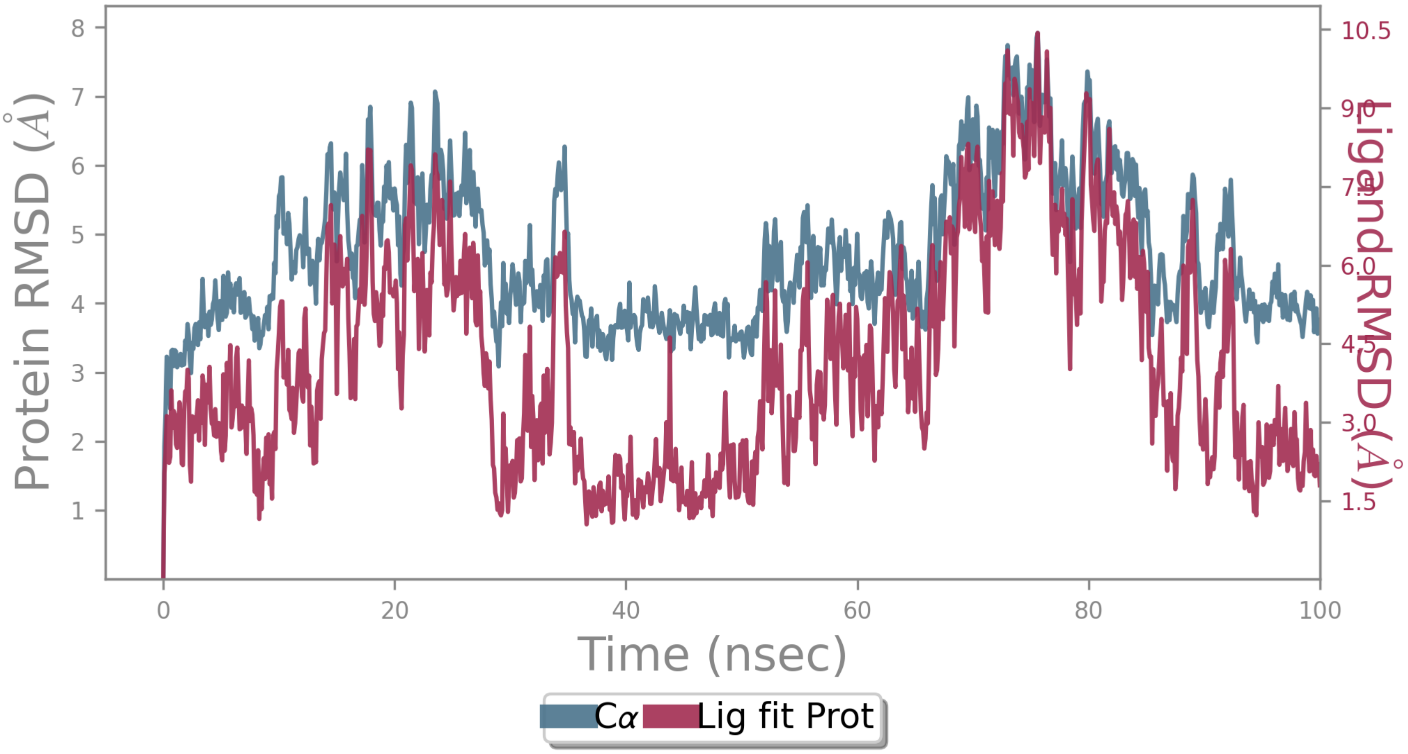

The Root Mean Square Deviation (RMSD) is used to measure the average change in displacement of a selection of atoms for a particular frame with respect to a reference frame. It is calculated for all frames in the trajectory. The RMSD for frame *x* is:

$$RMSD_x = \sqrt{\frac{1}{N}\sum_{i=1}^{N}(r_i'(t_x)) - r_i(t_{ref}))^2}$$

where *N* is the number of atoms in the atom selection; t_{ref} is the reference time, (typically the first frame is used as the reference and it is regarded as time *t*=0); and *r'* is the position of the selected atoms in frame *x* after superimposing on the reference frame, where frame *x* is recorded at time t_x. The procedure is repeated for every frame in the simulation trajectory.

Protein RMSD: The above plot shows the RMSD evolution of a protein (left *Y*-axis). All protein frames are first aligned on the reference frame backbone, and then the RMSD is calculated based on the atom selection. Monitoring the RMSD of the protein can give insights into its structural conformation throughout the simulation. RMSD analysis can indicate if the simulation has equilibrated — its fluctuations towards the end of the simulation are around some thermal average structure. Changes of the order of 1-3 Å are perfectly acceptable for small, globular proteins. Changes much larger than that, however, indicate that the protein is undergoing a large conformational change during the simulation. It is also important that your simulation converges — the RMSD values stabilize around a fixed value. If the RMSD of the protein is still increasing or decreasing on average at the end of the simulation, then your system has not equilibrated, and your simulation may not be long enough for rigorous analysis.

Ligand RMSD: Ligand RMSD (right *Y*-axis) indicates how stable the ligand is with respect to the protein and its binding pocket. In the above plot, 'Lig fit Prot' shows the RMSD of a ligand when the protein-ligand complex is first aligned on the protein backbone of the reference and then the RMSD of the ligand heavy atoms is measured. If the values observed are significantly larger than the RMSD of the protein, then it is likely that the ligand has diffused away from its initial binding site.

Protein RMSF

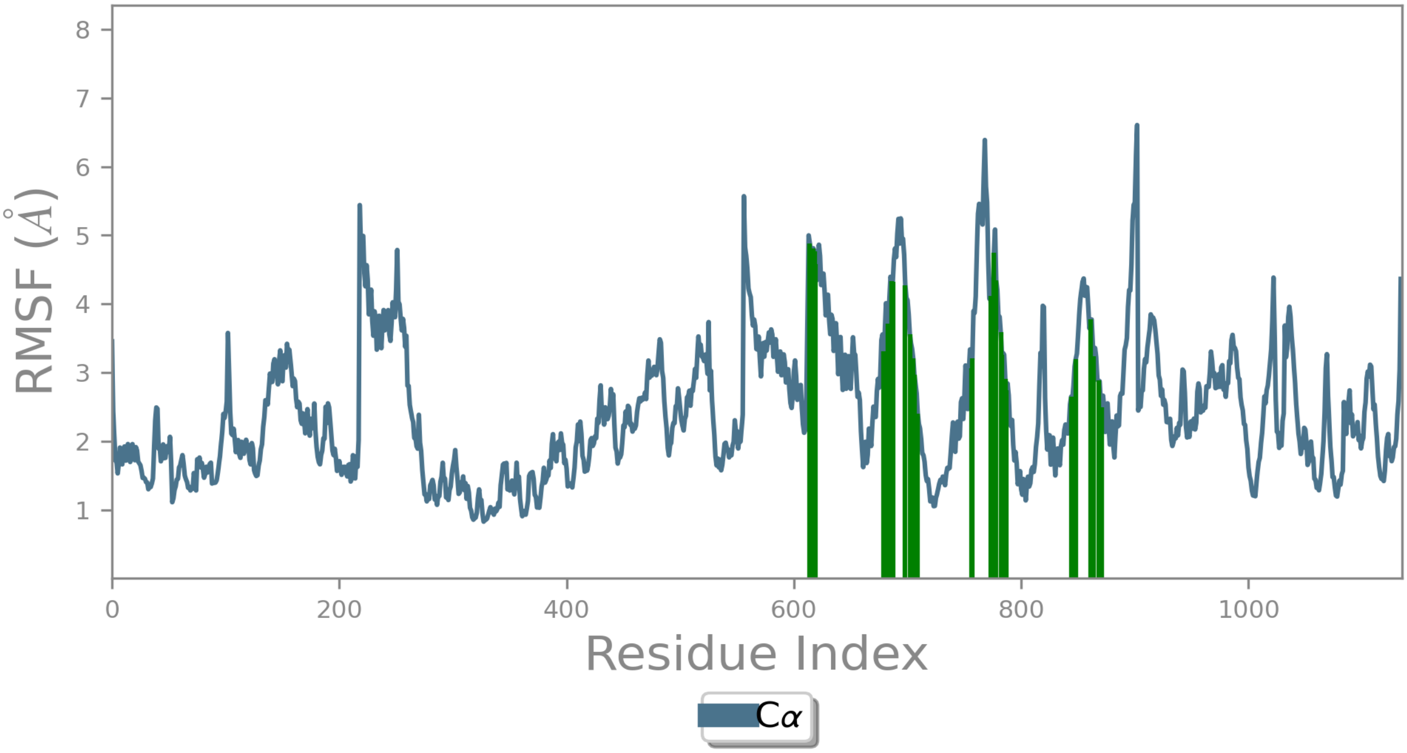

The Root Mean Square Fluctuation (RMSF) is useful for characterizing local changes along the protein chain. The RMSF for residue *i* is:

$$RMSF_i = \sqrt{\frac{1}{T}\sum_{t=1}^{T} < (r_i'(t)) - r_i(t_{ref}))^2 >}$$

where T is the trajectory time over which the RMSF is calculated, t_{ref} is the reference time, r_i is the position of residue *i*; *r'* is the position of atoms in residue *i* after superposition on the reference, and the angle brackets indicate that the average of the square distance is taken over the selection of atoms in the residue.

On this plot, peaks indicate areas of the protein that fluctuate the most during the simulation. Typically you will observe that the tails (*N*- and *C*-terminal) fluctuate more than any other part of the protein. Secondary structure elements like alpha helices and beta strands are usually more rigid than the unstructured part of the protein, and thus fluctuate less than the loop regions.

<u>Ligand Contacts</u>: Protein residues that interact with the ligand are marked with green-colored vertical bars.

Protein Secondary Structure

% Helix	% Strand	% Total SSE
30.78	24.48	55.26

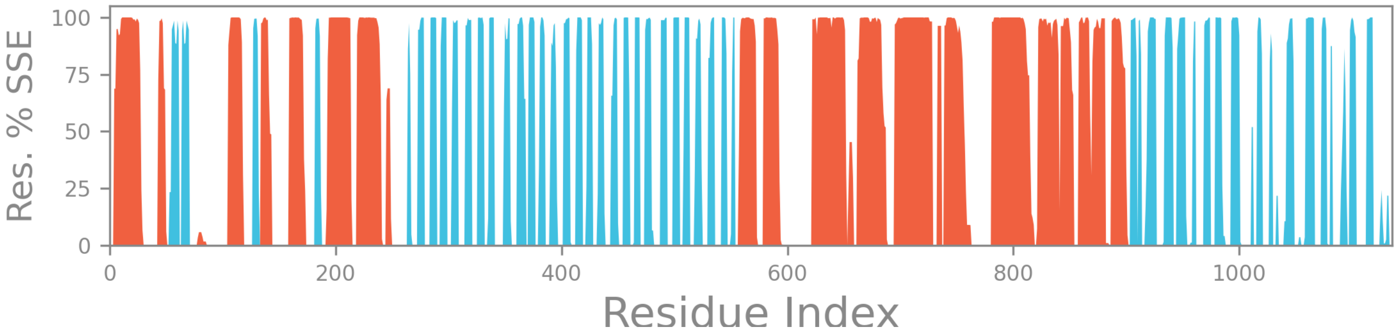

Protein secondary structure elements (SSE) like alpha-helices and beta-strands are monitored throughout the simulation. The plot above reports SSE distribution by residue index throughout the protein structure. The plot below summarizes the SSE composition for each trajectory frame over the course of the simulation, and the plot at the bottom monitors each residue and its SSE assignment over time.

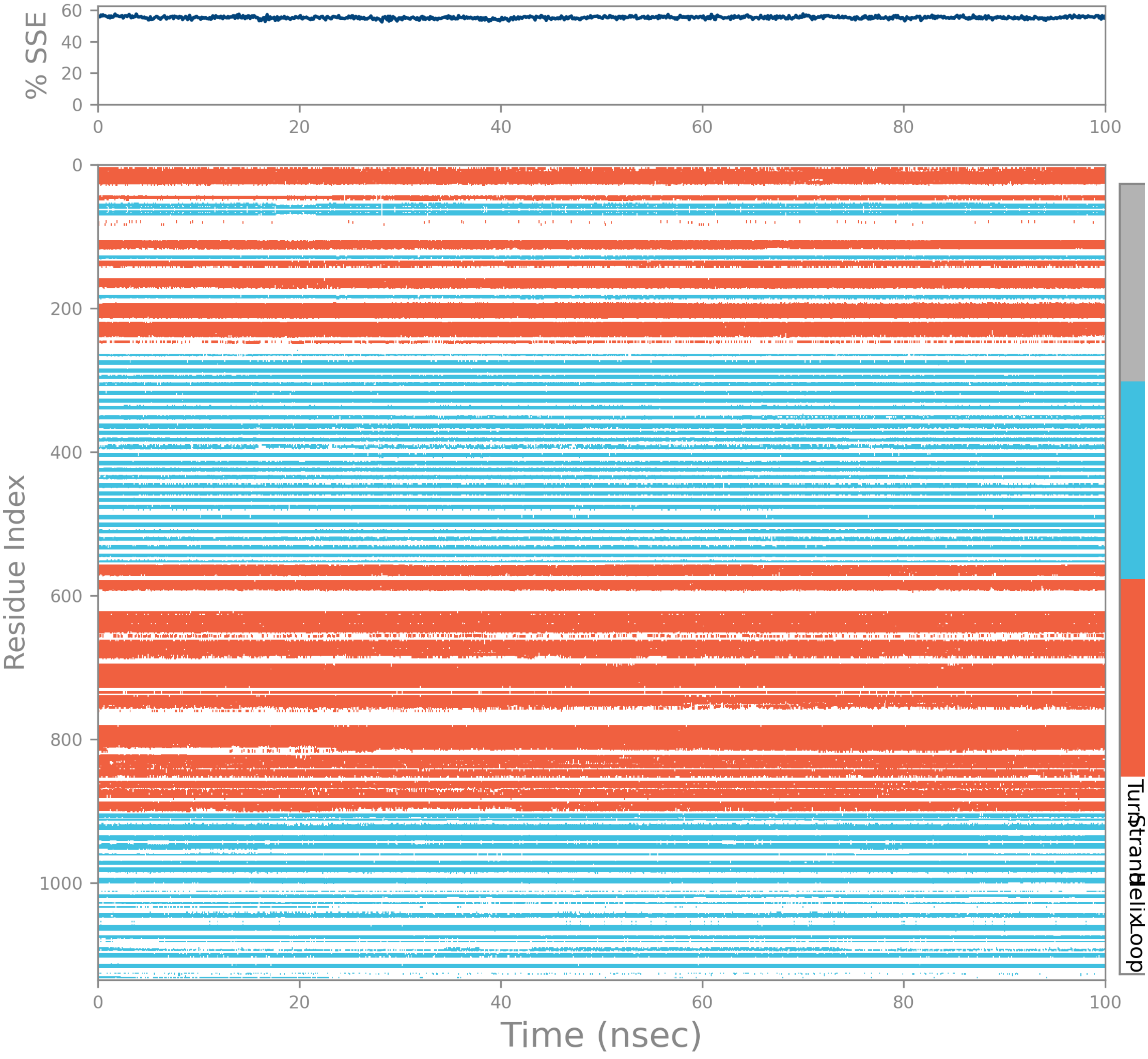

Ligand RMSF

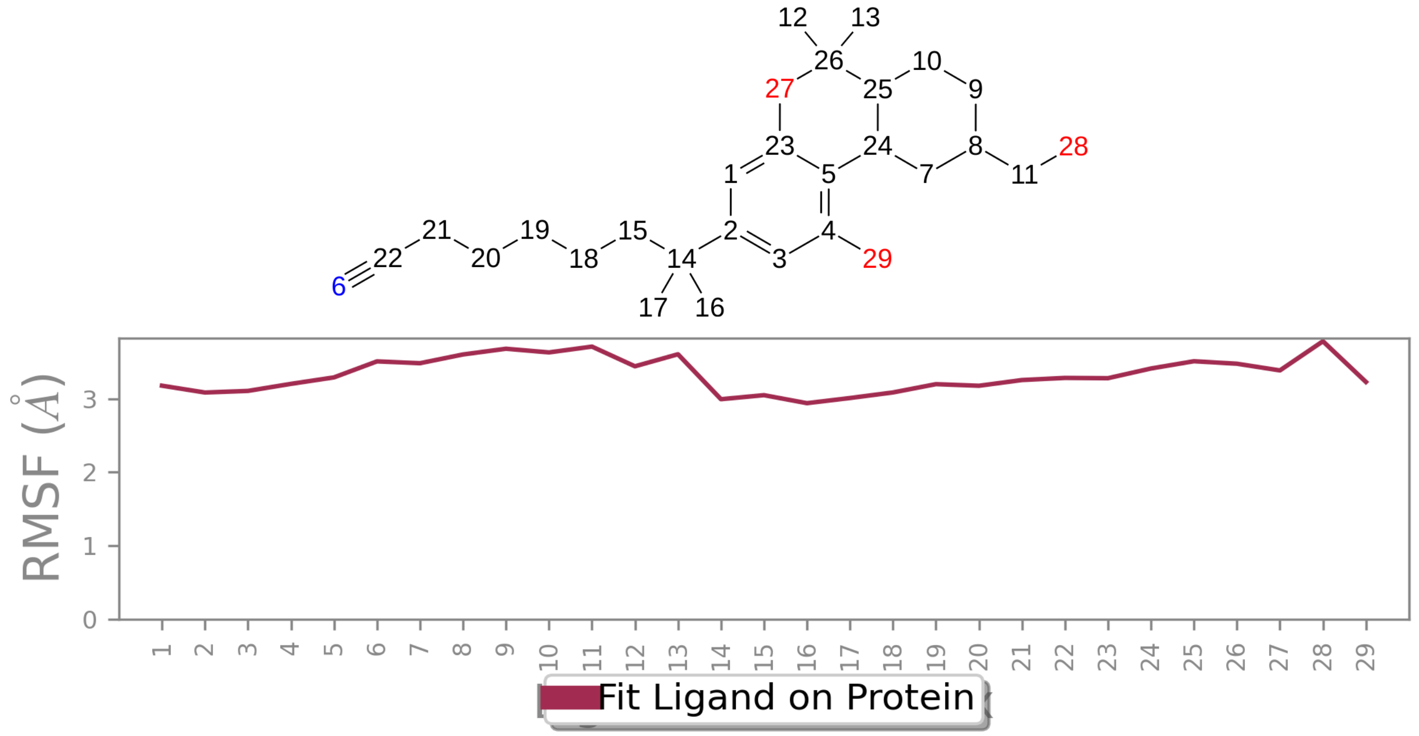

The Ligand Root Mean Square Fluctuation (L-RMSF) is useful for characterizing changes in the ligand atom positions. The RMSF for atom *i* is:

$$RMSF_i = \sqrt{\frac{1}{T}\sum_{t=1}^{T}(r'_i(t)) - r_i(t_{ref}))^2}$$

where *T* is the trajectory time over which the RMSF is calculated, t_{ref} is the reference time (usually for the first frame, and is regarded as the *zero* of time); *r* is the position of atom *i* in the reference at time t_{ref}, and *r'* is the position of atom *i* at time *t* after superposition on the reference frame.

Ligand RMSF shows the ligand's fluctuations broken down by atom, corresponding to the 2D structure in the top panel. The ligand RMSF may give you insights on how ligand fragments interact with the protein and their entropic role in the binding event. In the bottom panel, the 'Fit Ligand on Protein' line shows the ligand fluctuations, with respect to the protein. The protein-ligand complex is first aligned on the protein backbone and then the ligand RMSF is measured on the ligand heavy atoms.

Protein-Ligand Contacts

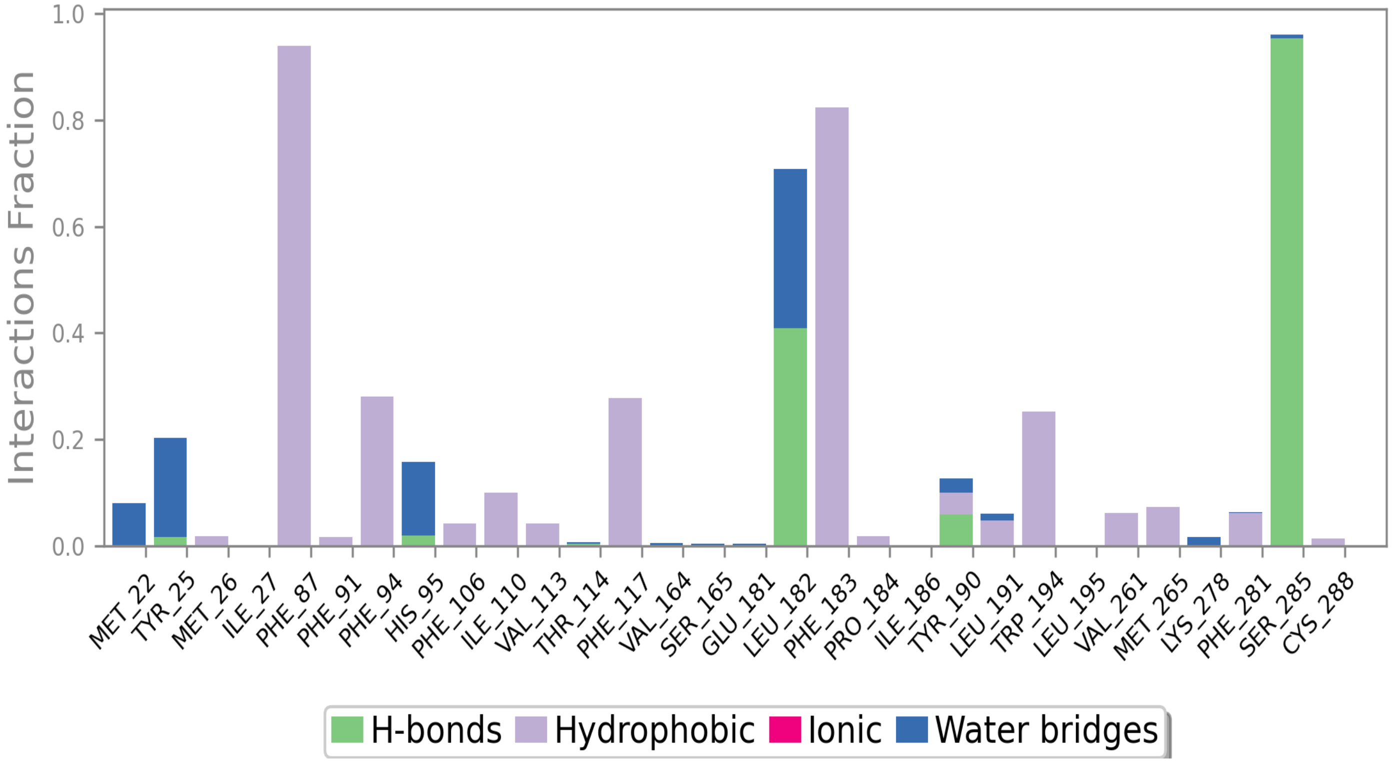

Protein interactions with the ligand can be monitored throughout the simulation. These interactions can be categorized by type and summarized, as shown in the plot above. Protein-ligand interactions (or 'contacts') are categorized into four types: Hydrogen Bonds, Hydrophobic, Ionic and Water Bridges. Each interaction type contains more specific subtypes, which can be explored through the 'Simulation Interactions Diagram' panel. The stacked bar charts are normalized over the course of the trajectory: for example, a value of 0.7 suggests that 70% of the simulation time the specific interaction is maintained. Values over 1.0 are possible as some protein residue may make multiple contacts of same subtype with the ligand.

Hydrogen Bonds: (H-bonds) play a significant role in ligand binding. Consideration of hydrogen-bonding properties in drug design is important because of their strong influence on drug specificity, metabolization and adsorption. Hydrogen bonds between a protein and a ligand can be further broken down into four subtypes: backbone acceptor; backbone donor; side-chain acceptor; side-chain donor.
The current geometric criteria for protein-ligand H-bond is: distance of 2.5 Å between the donor and acceptor atoms (D—H···A); a donor angle of ≥120° between the donor-hydrogen-acceptor atoms (D—H···A); and an acceptor angle of ≥90° between the hydrogen-acceptor-bonded_atom atoms (H···A—X).

Hydrophobic contacts: fall into three subtypes: π-Cation; π-π; and Other, non-specific interactions. Generally these type of interactions involve a hydrophobic amino acid and an aromatic or aliphatic group on the ligand, but we have extended this category to also include π-Cation interactions.
The current geometric criteria for hydrophobic interactions is as follows: π-Cation — Aromatic and charged groups within 4.5 Å; π-π — Two aromatic groups stacked face-to-face or face-to-edge; Other — A non-specific hydrophobic sidechain within 3.6 Å of a ligand's aromatic or aliphatic carbons.

Ionic interactions: or polar interactions, are between two oppositely charged atoms that are within 3.7 Å of each other and do not involve a hydrogen bond. We also monitor Protein-Metal-Ligand interactions, which are defined by a metal ion coordinated within 3.4 Å of protein's and ligand's heavy atoms (except carbon). All ionic interactions are broken down into two subtypes: those mediated by a protein backbone or side chains.

Water Bridges: are hydrogen-bonded protein-ligand interactions mediated by a water molecule. The hydrogen-bond geometry is slightly relaxed from the standard H-bond definition.
The current geometric criteria for a protein-water or water-ligand H-bond are: a distance of 2.8 Å between the donor and acceptor atoms (D—H···A); a donor angle of ≥110° between the donor-hydrogen-acceptor atoms (D—H···A); and an acceptor angle of ≥90° between the hydrogen-acceptor-bonded_atom atoms (H···A—X).

Protein-Ligand Contacts (cont.)

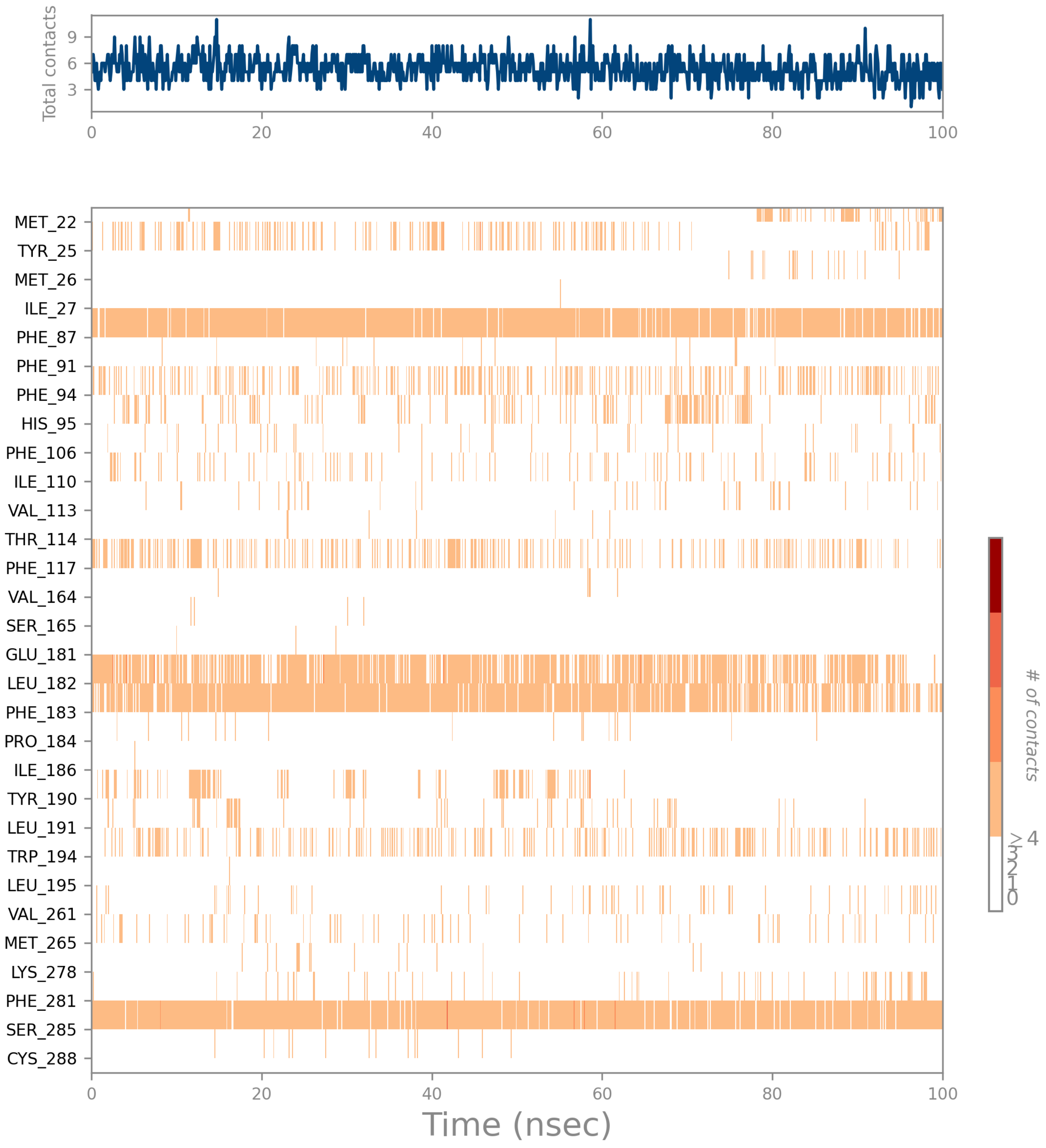

A timeline representation of the interactions and contacts (**H-bonds**, **Hydrophobic**, **Ionic**, **Water bridges**) summarized in the previous page. The top panel shows the total number of specific contacts the protein makes with the ligand over the course of the trajectory. The bottom panel shows which residues interact with the ligand in each trajectory frame. Some residues make more than one specific contact with the ligand, which is represented by a darker shade of orange, according to the scale to the right of the plot.

Ligand-Protein Contacts

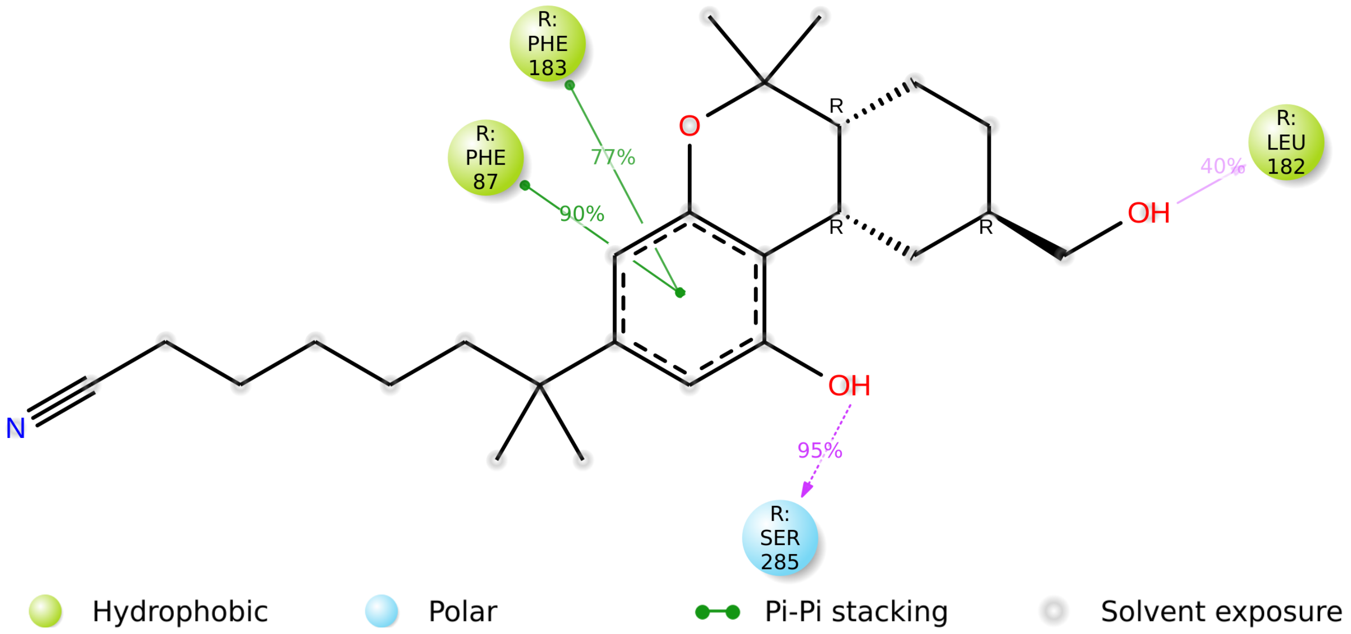

A schematic of detailed ligand atom interactions with the protein residues. Interactions that occur more than **30.0%** of the simulation time in the selected trajectory (0.00 through 100.00 nsec), are shown.
Note: it is possible to have interactions with >100% as some residues may have multiple interactions of a single type with the same ligand atom. For example, the ARG side chain has four H-bond donors that can all hydrogen-bond to a single H-bond acceptor.

Ligand Torsion Profile

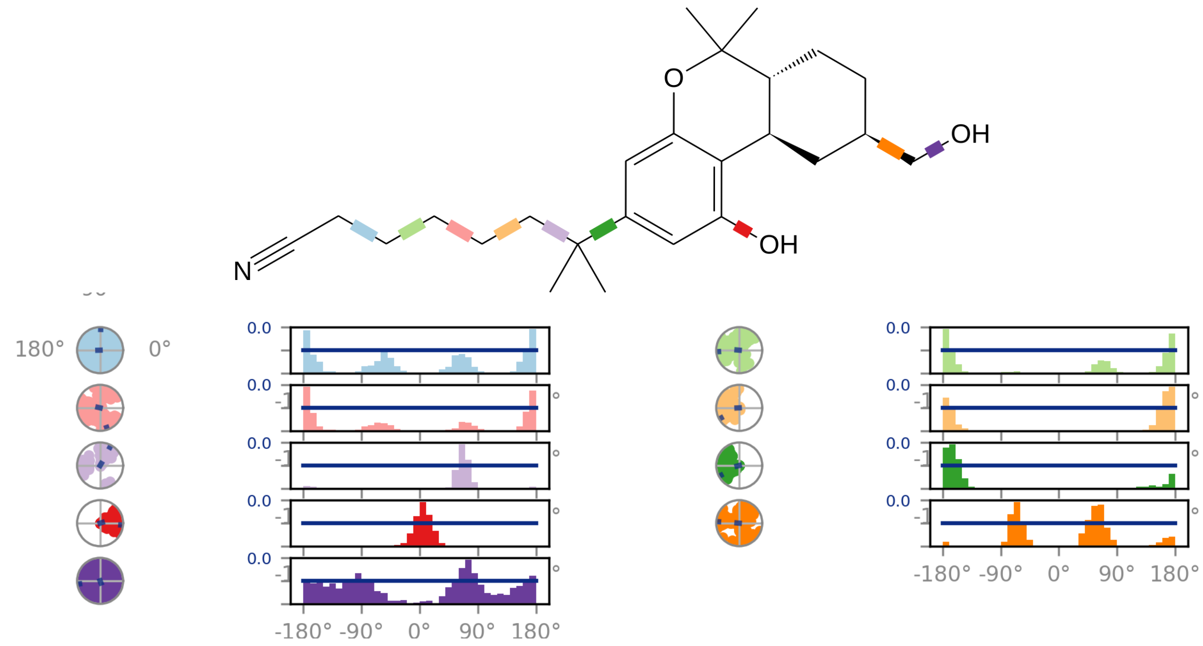

The ligand torsions plot summarizes the conformational evolution of every rotatable bond (RB) in the ligand throughout the simulation trajectory (0.00 through 100.00 nsec). The top panel shows the 2d schematic of a ligand with color-coded rotatable bonds. Each rotatable bond torsion is accompanied by a dial plot and bar plots of the same color.

Dial (or radial) plots describe the conformation of the torsion throughout the course of the simulation. The beginning of the simulation is in the center of the radial plot and the time evolution is plotted radially outwards.

The bar plots summarize the data on the dial plots, by showing the probability density of the torsion. If torsional potential information is available, the plot also shows the potential of the rotatable bond (by summing the potential of the related torsions). The values of the potential are on the left *Y*-axis of the chart, and are expressed in *kcal/mol*. Looking at the histogram and torsion potential relationships may give insights into the conformational strain the ligand undergoes to maintain a protein-bound conformation.

Ligand Properties

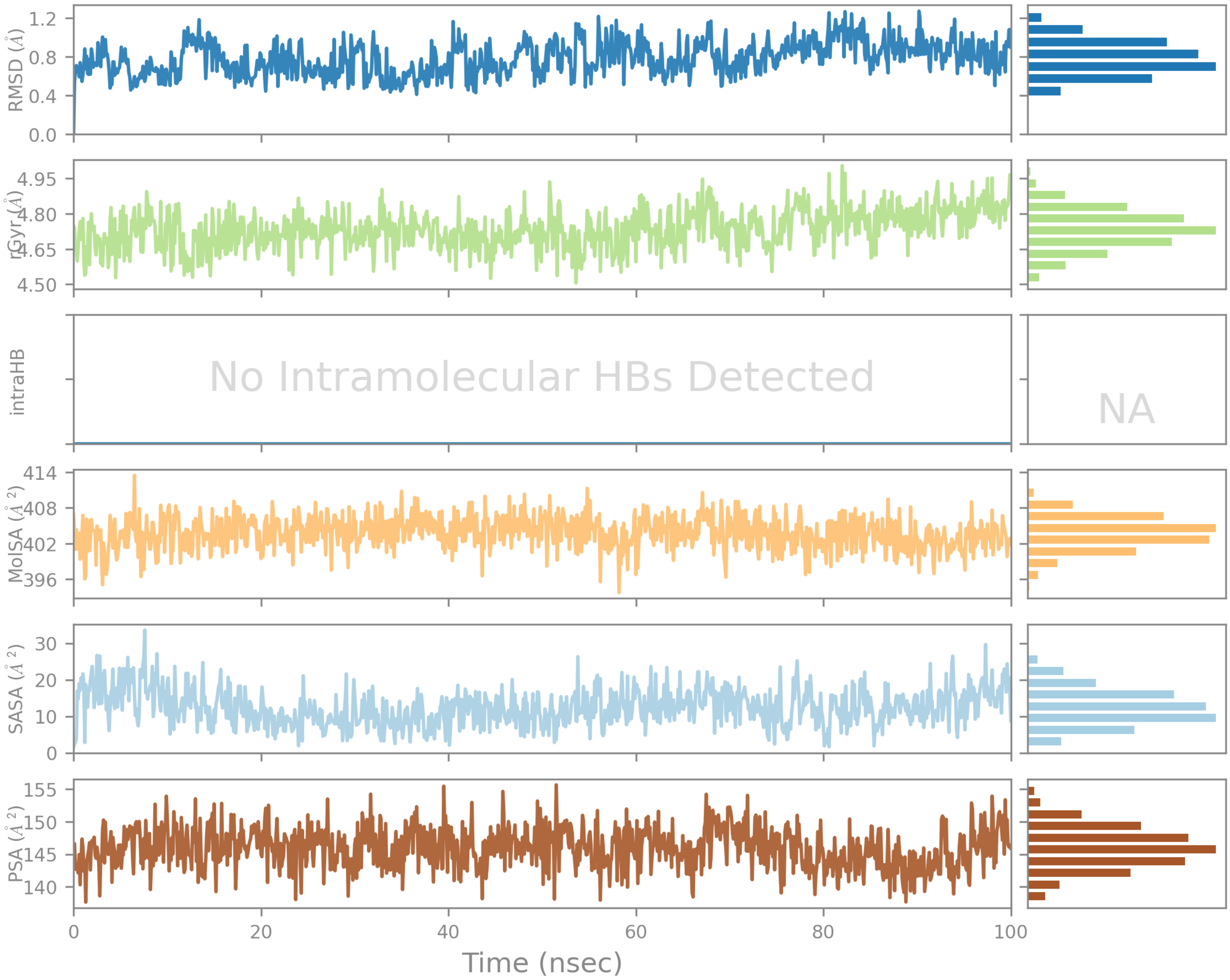

Ligand RMSD: Root mean square deviation of a ligand with respect to the reference conformation (typically the first frame is used as the reference and it is regarded as time *t*=0).

Radius of Gyration (rGyr): Measures the 'extendedness' of a ligand, and is equivalent to its principal moment of inertia.

Intramolecular Hydrogen Bonds (intraHB): Number of internal hydrogen bonds (HB) within a ligand molecule.

Molecular Surface Area (MolSA): Molecular surface calculation with 1.4 Å probe radius. This value is equivalent to a van der Waals surface area.

Solvent Accessible Surface Area (SASA): Surface area of a molecule accessible by a water molecule.

Polar Surface Area (PSA): Solvent accessible surface area in a molecule contributed only by oxygen and nitrogen atoms.

Simulation Interactions Diagram Report

Simulation Details

Jobname: desmond_md_job_6KPF_Boremexin_C
Entry title: Boremexin C

CPU #	Job Type	Ensemble	Temp. [K]	Sim. Time [ns]	# Atoms	# Waters	Charge
1	mdsim	NPT	300.0	100.102	162206	48083	0

Protein Information

Tot. Residues	Prot. Chain(s)	Res. in Chain(s)	# Atoms	# Heavy Atoms	Charge
1135	'A', 'B', 'C', 'R', 'S'	dict_values([218, 338, 57, 290, 232])	17236	8842	-3

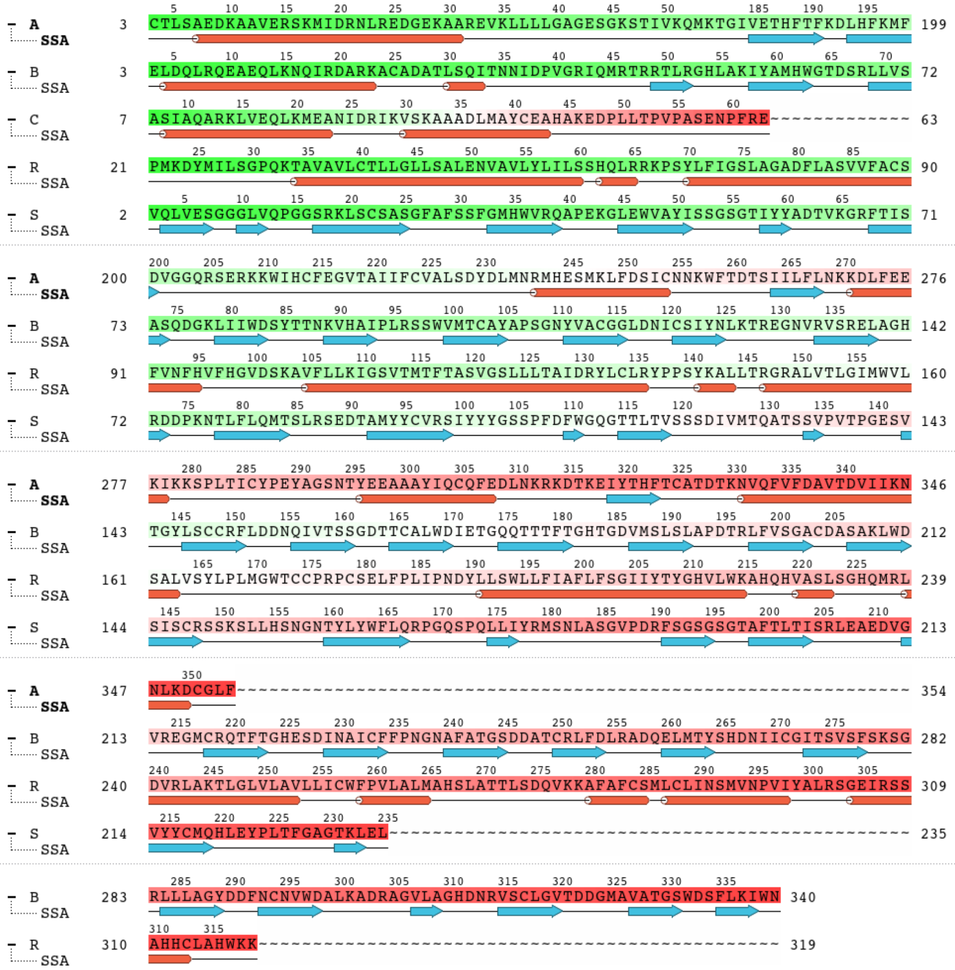

Ligand Information

SMILES	COC(=O)[C@H](O)/C=C/c(c(O)c(c1C)OC)c(c12)Oc3c(OC2=O)cc(O)cc3C

PDB Name	'UNK'
Num. of Atoms	50 (total) 30 (heavy)
Atomic Mass	416.388 au
Charge	0
Mol. Formula	C21H20O9
Num. of Fragments	2
Num. of Rot. Bonds	8

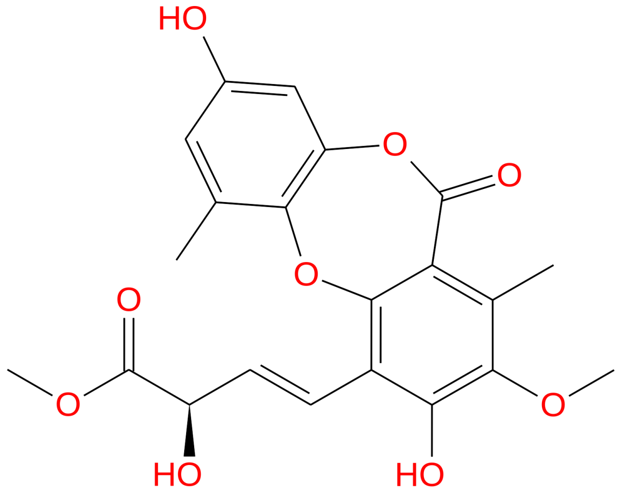

Counter Ion/Salt Information

Type	Num.	Concentration [mM]	Total Charge
Na	137	51.804	+137
Cl	134	50.670	-134

Protein-Ligand RMSD

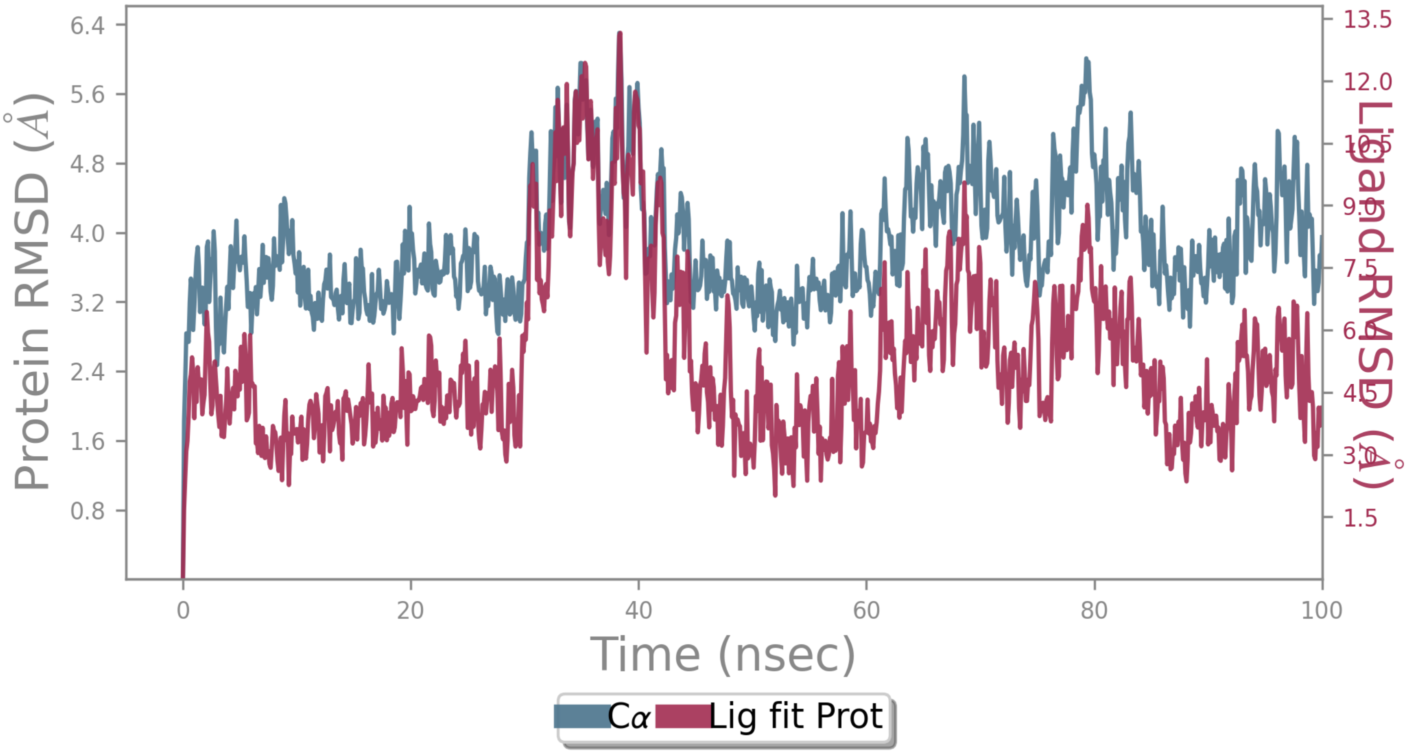

The Root Mean Square Deviation (RMSD) is used to measure the average change in displacement of a selection of atoms for a particular frame with respect to a reference frame. It is calculated for all frames in the trajectory. The RMSD for frame *x* is:

$$RMSD_x = \sqrt{\frac{1}{N}\sum_{i=1}^{N}(r'_i(t_x)) - r_i(t_{ref}))^2}$$

where *N* is the number of atoms in the atom selection; t_{ref} is the reference time, (typically the first frame is used as the reference and it is regarded as time *t*=0); and *r'* is the position of the selected atoms in frame *x* after superimposing on the reference frame, where frame *x* is recorded at time t_x. The procedure is repeated for every frame in the simulation trajectory.

Protein RMSD: The above plot shows the RMSD evolution of a protein (left *Y*-axis). All protein frames are first aligned on the reference frame backbone, and then the RMSD is calculated based on the atom selection. Monitoring the RMSD of the protein can give insights into its structural conformation throughout the simulation. RMSD analysis can indicate if the simulation has equilibrated — its fluctuations towards the end of the simulation are around some thermal average structure. Changes of the order of 1-3 Å are perfectly acceptable for small, globular proteins. Changes much larger than that, however, indicate that the protein is undergoing a large conformational change during the simulation. It is also important that your simulation converges — the RMSD values stabilize around a fixed value. If the RMSD of the protein is still increasing or decreasing on average at the end of the simulation, then your system has not equilibrated, and your simulation may not be long enough for rigorous analysis.

Ligand RMSD: Ligand RMSD (right *Y*-axis) indicates how stable the ligand is with respect to the protein and its binding pocket. In the above plot, 'Lig fit Prot' shows the RMSD of a ligand when the protein-ligand complex is first aligned on the protein backbone of the reference and then the RMSD of the ligand heavy atoms is measured. If the values observed are significantly larger than the RMSD of the protein, then it is likely that the ligand has diffused away from its initial binding site.

Protein RMSF

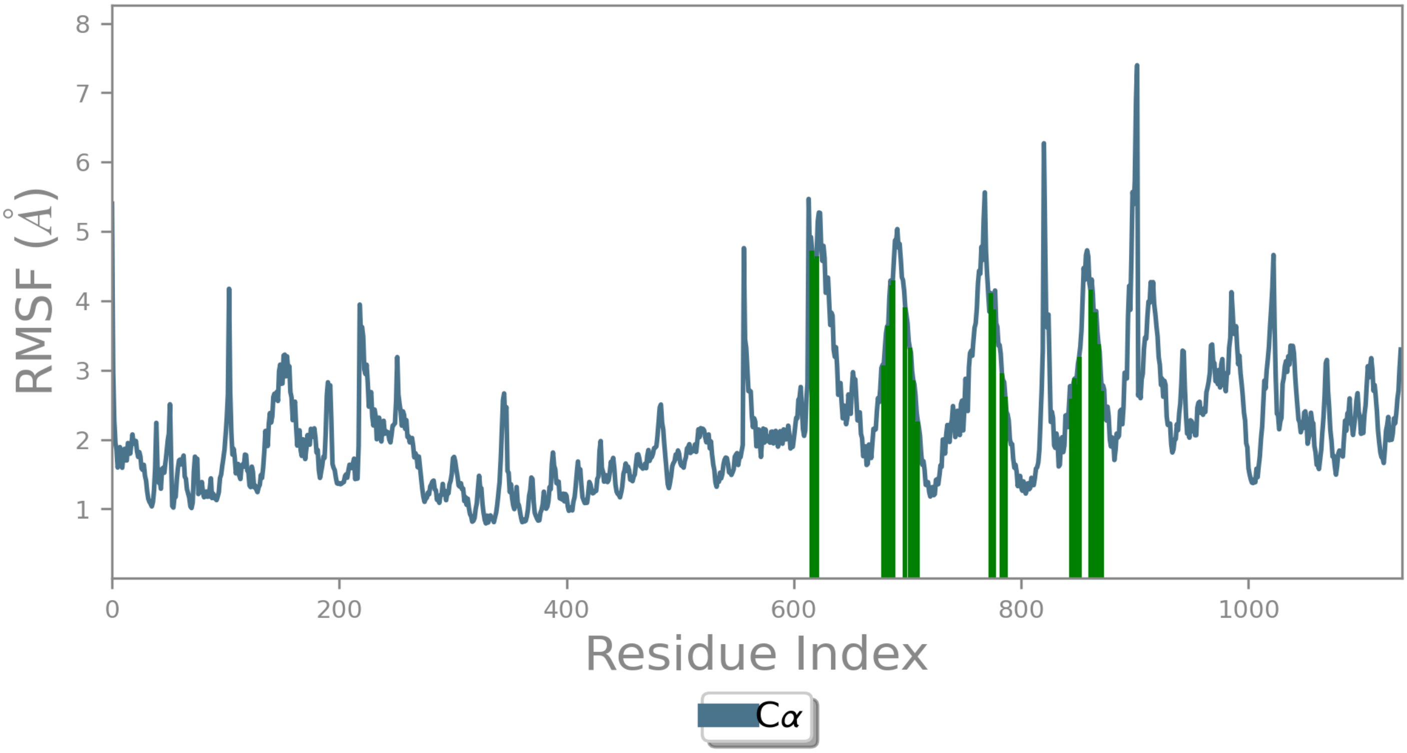

The Root Mean Square Fluctuation (RMSF) is useful for characterizing local changes along the protein chain. The RMSF for residue *i* is:

$$RMSF_i = \sqrt{\frac{1}{T}\sum_{t=1}^{T} < (r_i'(t)) - r_i(t_{ref}))^2 >}$$

where *T* is the trajectory time over which the RMSF is calculated, t_{ref} is the reference time, r_i is the position of residue *i*; *r'* is the position of atoms in residue *i* after superposition on the reference, and the angle brackets indicate that the average of the square distance is taken over the selection of atoms in the residue.

On this plot, peaks indicate areas of the protein that fluctuate the most during the simulation. Typically you will observe that the tails (*N*- and *C*-terminal) fluctuate more than any other part of the protein. Secondary structure elements like alpha helices and beta strands are usually more rigid than the unstructured part of the protein, and thus fluctuate less than the loop regions.

Ligand Contacts: Protein residues that interact with the ligand are marked with green-colored vertical bars.

Protein Secondary Structure

% Helix	% Strand	% Total SSE
30.78	24.47	55.25

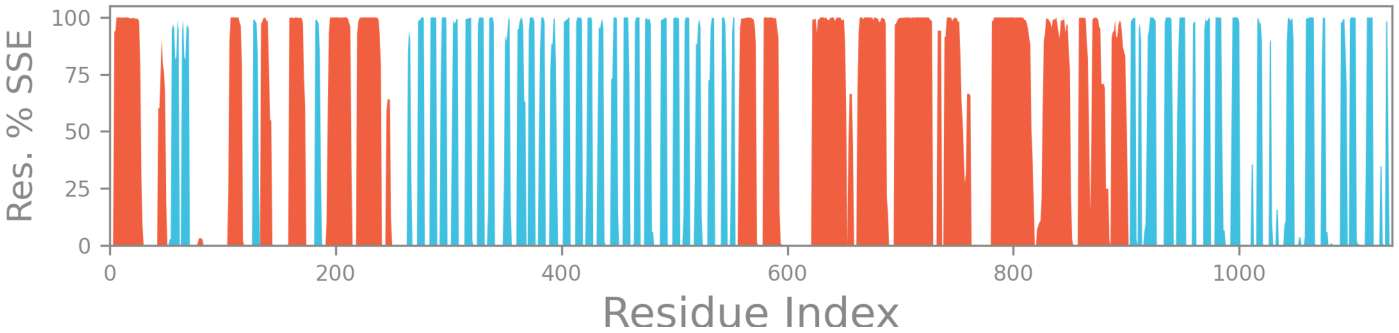

Protein secondary structure elements (SSE) like alpha-helices and beta-strands are monitored throughout the simulation. The plot above reports SSE distribution by residue index throughout the protein structure. The plot below summarizes the SSE composition for each trajectory frame over the course of the simulation, and the plot at the bottom monitors each residue and its SSE assignment over time.

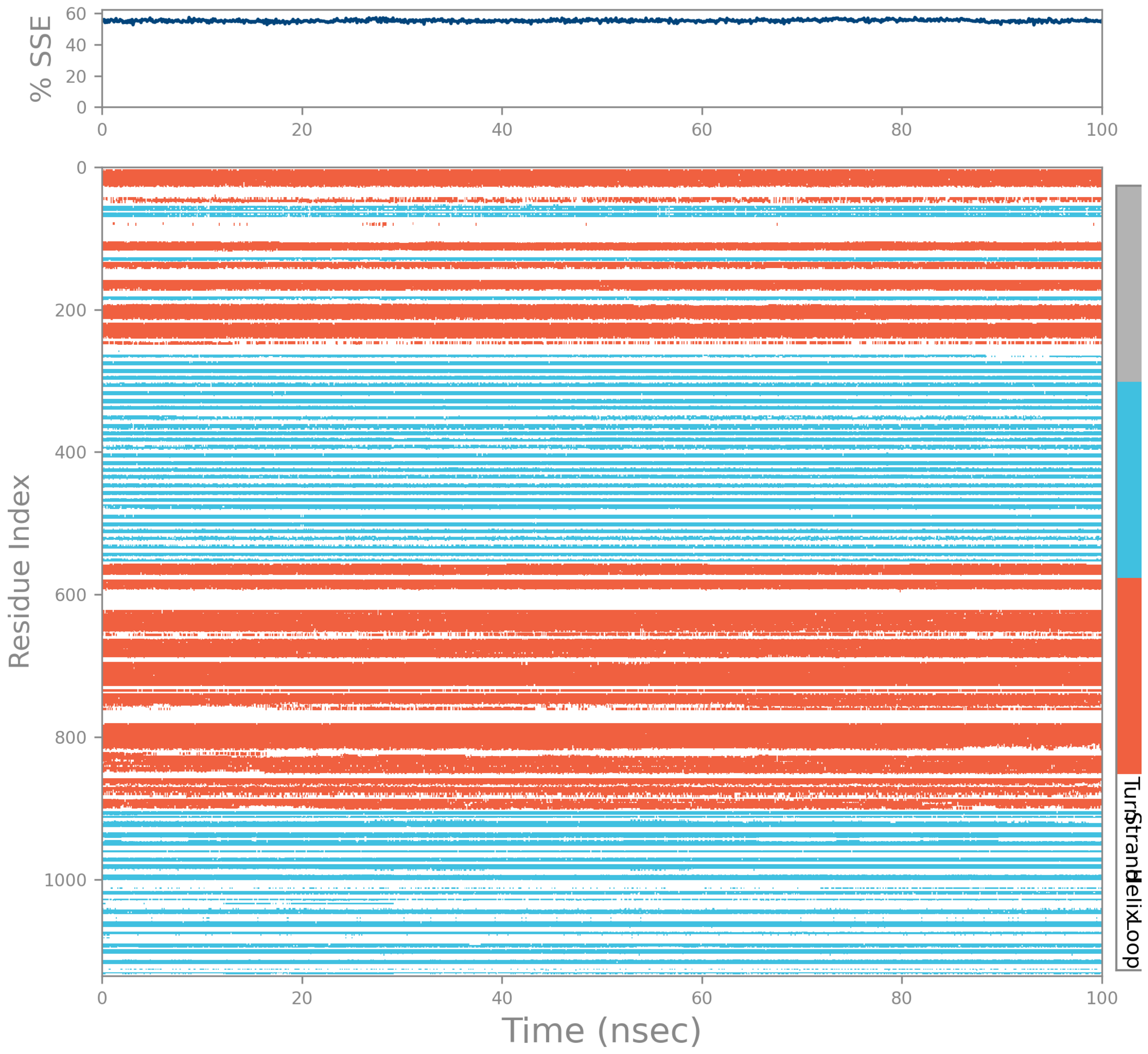

Ligand RMSF

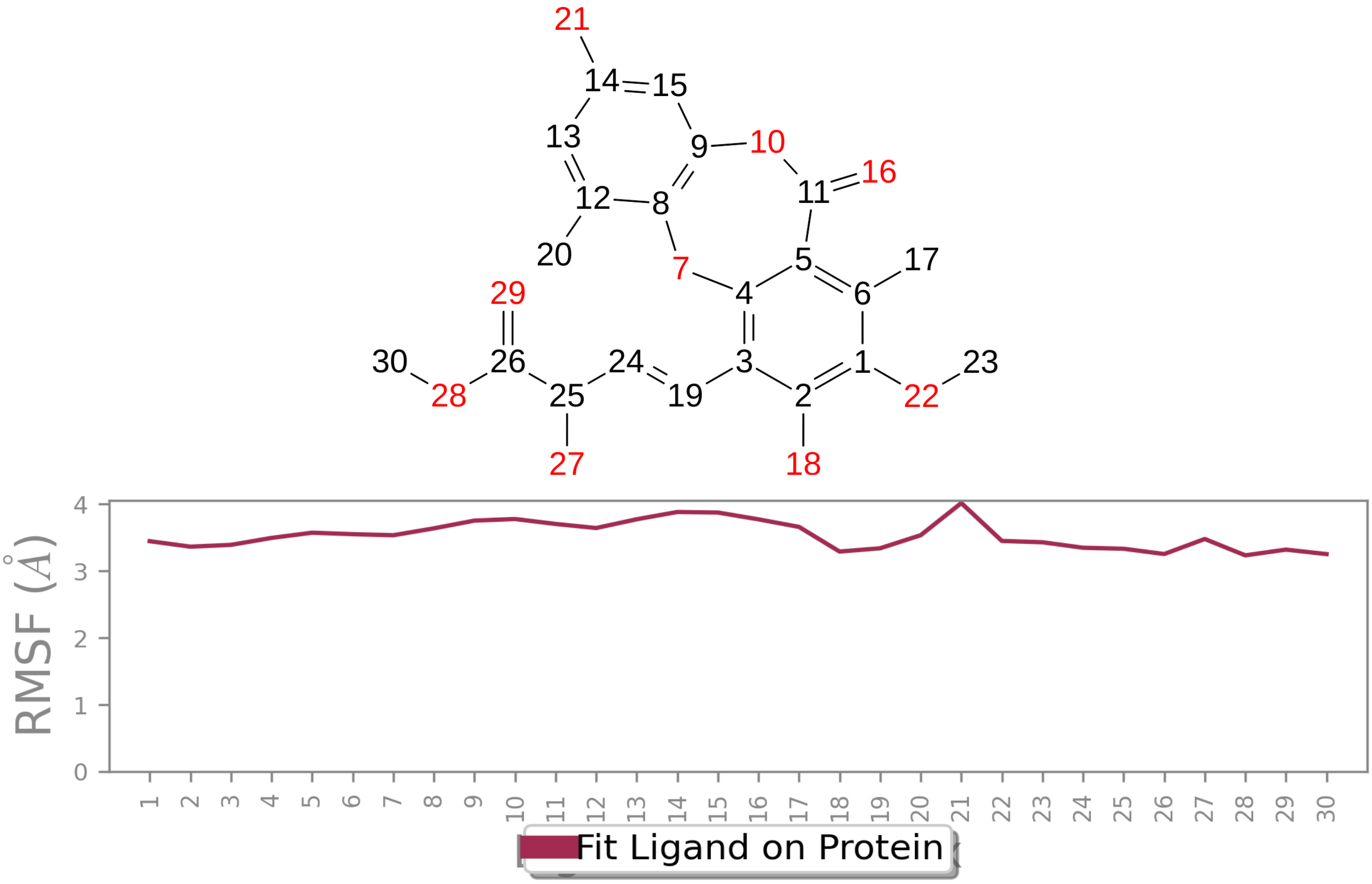

The Ligand Root Mean Square Fluctuation (L-RMSF) is useful for characterizing changes in the ligand atom positions. The RMSF for atom *i* is:

$$RMSF_i = \sqrt{\frac{1}{T}\sum_{t=1}^{T}(r_i'(t)) - r_i(t_{ref}))^2}$$

where *T* is the trajectory time over which the RMSF is calculated, t_{ref} is the reference time (usually for the first frame, and is regarded as the *zero* of time); *r* is the position of atom *i* in the reference at time t_{ref}, and *r'* is the position of atom *i* at time *t* after superposition on the reference frame.

Ligand RMSF shows the ligand's fluctuations broken down by atom, corresponding to the 2D structure in the top panel. The ligand RMSF may give you insights on how ligand fragments interact with the protein and their entropic role in the binding event. In the bottom panel, the 'Fit Ligand on Protein' line shows the ligand fluctuations, with respect to the protein. The protein-ligand complex is first aligned on the protein backbone and then the ligand RMSF is measured on the ligand heavy atoms.

Protein-Ligand Contacts

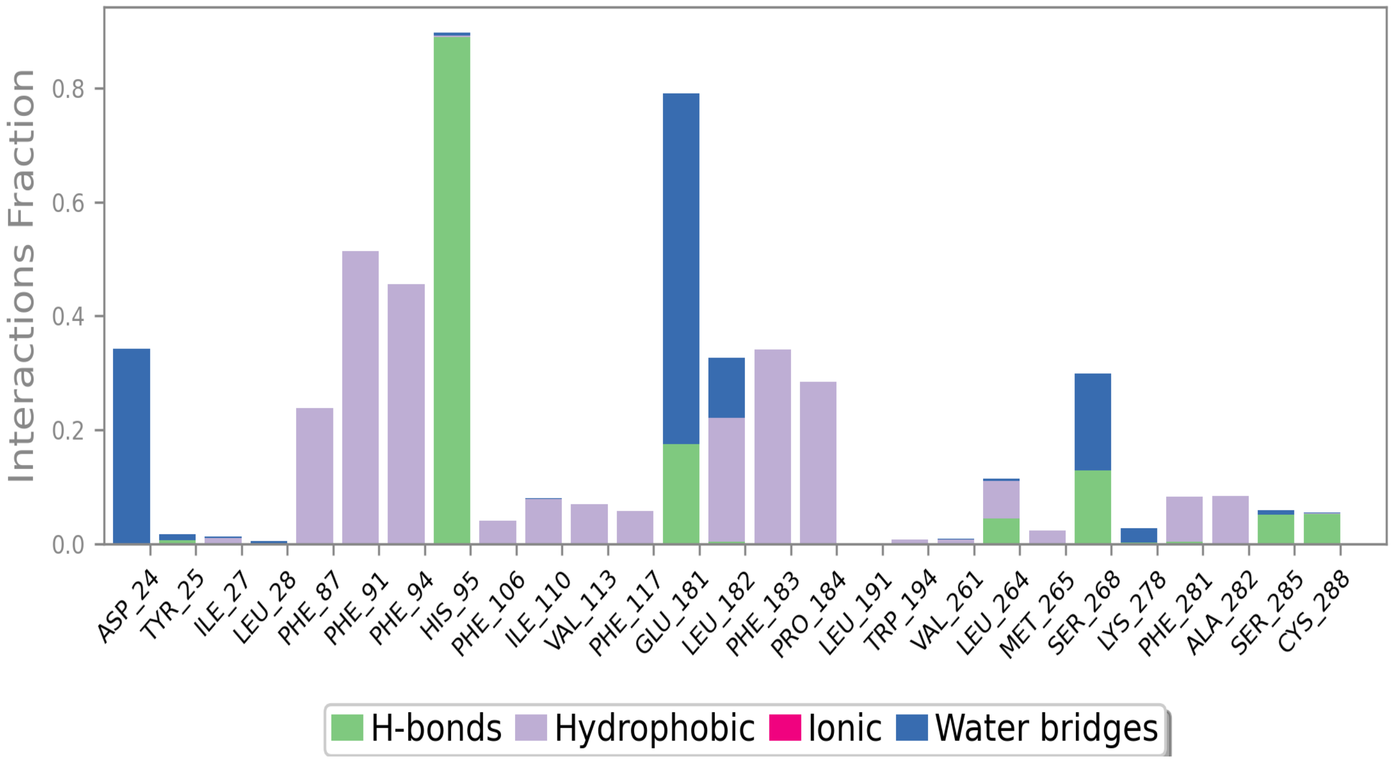

Protein interactions with the ligand can be monitored throughout the simulation. These interactions can be categorized by type and summarized, as shown in the plot above. Protein-ligand interactions (or 'contacts') are categorized into four types: Hydrogen Bonds, Hydrophobic, Ionic and Water Bridges. Each interaction type contains more specific subtypes, which can be explored through the 'Simulation Interactions Diagram' panel. The stacked bar charts are normalized over the course of the trajectory: for example, a value of 0.7 suggests that 70% of the simulation time the specific interaction is maintained. Values over 1.0 are possible as some protein residue may make multiple contacts of same subtype with the ligand.

Hydrogen Bonds: (H-bonds) play a significant role in ligand binding. Consideration of hydrogen-bonding properties in drug design is important because of their strong influence on drug specificity, metabolization and adsorption. Hydrogen bonds between a protein and a ligand can be further broken down into four subtypes: backbone acceptor; backbone donor; side-chain acceptor; side-chain donor.
The current geometric criteria for protein-ligand H-bond is: distance of 2.5 Å between the donor and acceptor atoms (D—H···A); a donor angle of ≥120° between the donor-hydrogen-acceptor atoms (D—H···A); and an acceptor angle of ≥90° between the hydrogen-acceptor-bonded_atom atoms (H···A—X).

Hydrophobic contacts: fall into three subtypes: π-Cation; π-π; and Other, non-specific interactions. Generally these type of interactions involve a hydrophobic amino acid and an aromatic or aliphatic group on the ligand, but we have extended this category to also include π-Cation interactions.
The current geometric criteria for hydrophobic interactions is as follows: π-Cation — Aromatic and charged groups within 4.5 Å; π-π — Two aromatic groups stacked face-to-face or face-to-edge; Other — A non-specific hydrophobic sidechain within 3.6 Å of a ligand's aromatic or aliphatic carbons.

Ionic interactions: or polar interactions, are between two oppositely charged atoms that are within 3.7 Å of each other and do not involve a hydrogen bond. We also monitor Protein-Metal-Ligand interactions, which are defined by a metal ion coordinated within 3.4 Å of protein's and ligand's heavy atoms (except carbon). All ionic interactions are broken down into two subtypes: those mediated by a protein backbone or side chains.

Water Bridges: are hydrogen-bonded protein-ligand interactions mediated by a water molecule. The hydrogen-bond geometry is slightly relaxed from the standard H-bond definition.
The current geometric criteria for a protein-water or water-ligand H-bond are: a distance of 2.8 Å between the donor and acceptor atoms (D—H···A); a donor angle of ≥110° between the donor-hydrogen-acceptor atoms (D—H···A); and an acceptor angle of ≥90° between the hydrogen-acceptor-bonded_atom atoms (H···A—X).

Protein-Ligand Contacts (cont.)

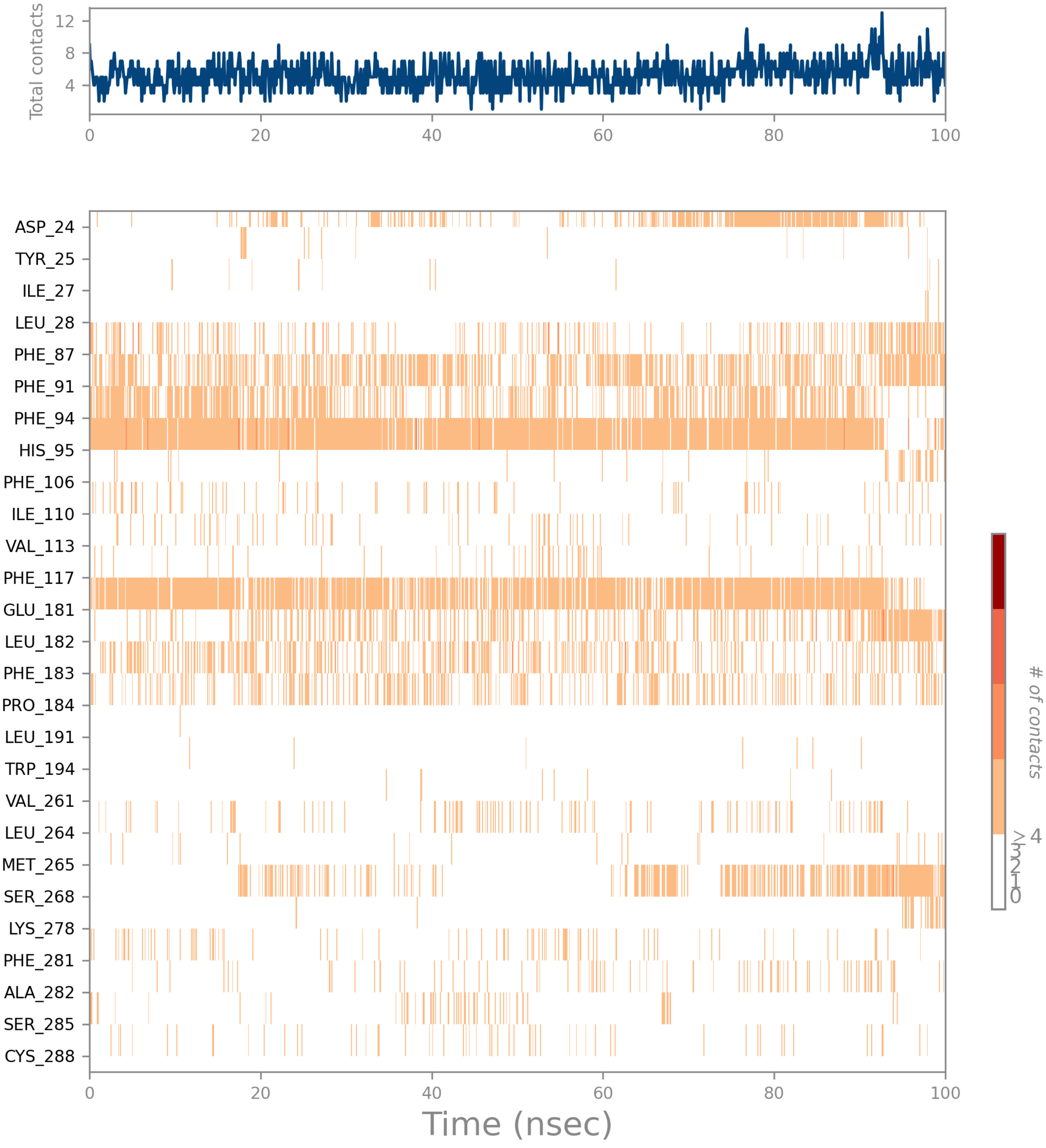

A timeline representation of the interactions and contacts (**H-bonds**, **Hydrophobic**, **Ionic**, **Water bridges**) summarized in the previous page. The top panel shows the total number of specific contacts the protein makes with the ligand over the course of the trajectory. The bottom panel shows which residues interact with the ligand in each trajectory frame. Some residues make more than one specific contact with the ligand, which is represented by a darker shade of orange, according to the scale to the right of the plot.

Ligand-Protein Contacts

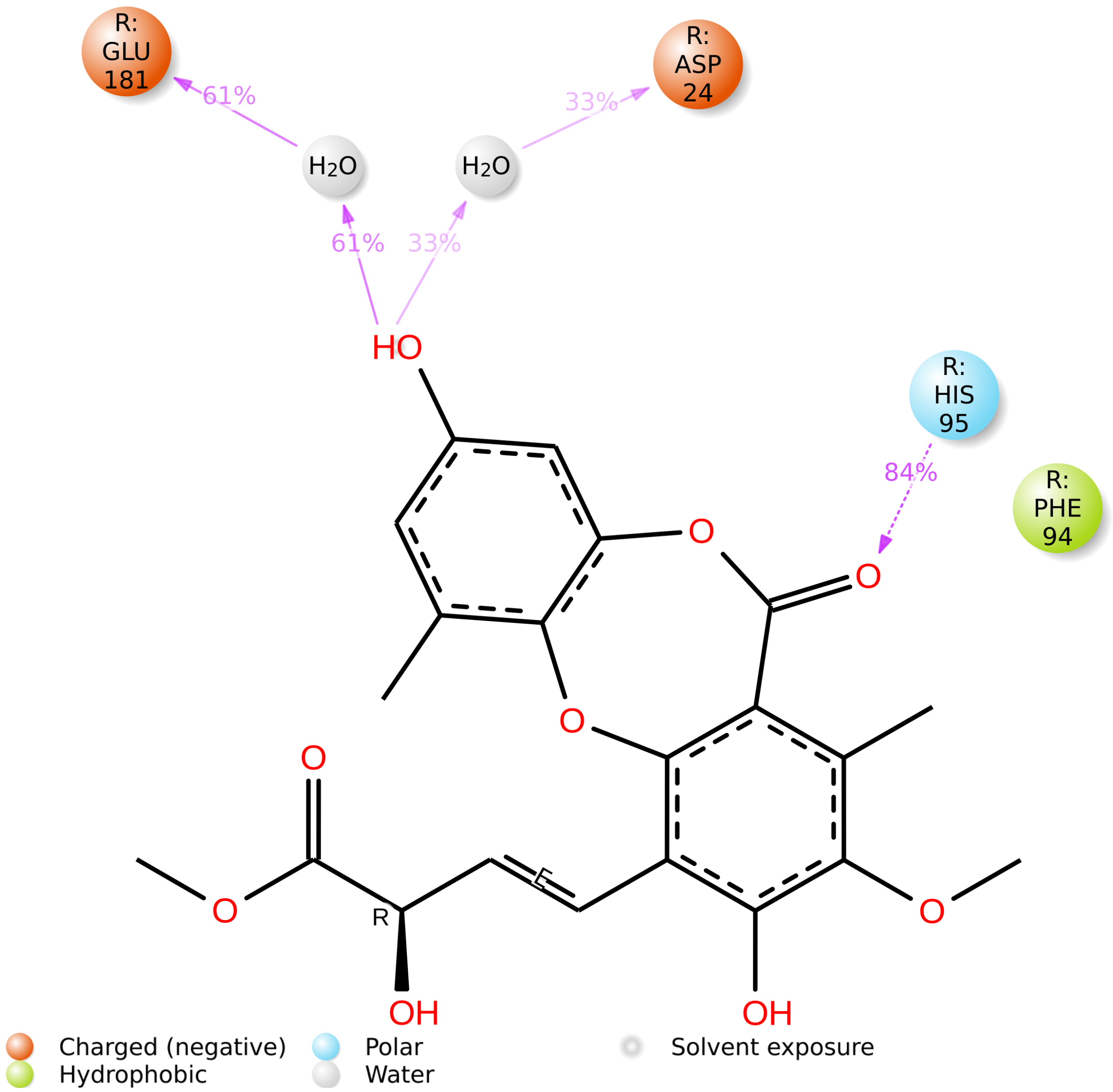

A schematic of detailed ligand atom interactions with the protein residues. Interactions that occur more than **30.0%** of the simulation time in the selected trajectory (0.00 through 100.00 nsec), are shown.
Note: it is possible to have interactions with >100% as some residues may have multiple interactions of a single type with the same ligand atom. For example, the ARG side chain has four H-bond donors that can all hydrogen-bond to a single H-bond acceptor.

Ligand Torsion Profile

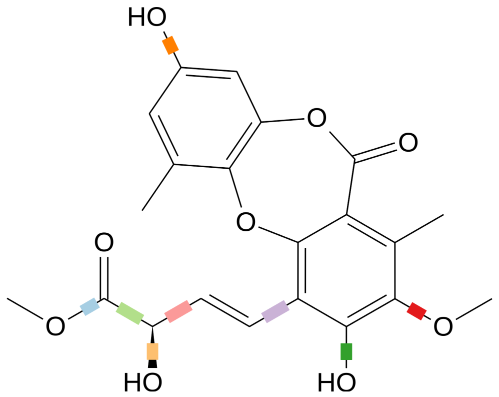

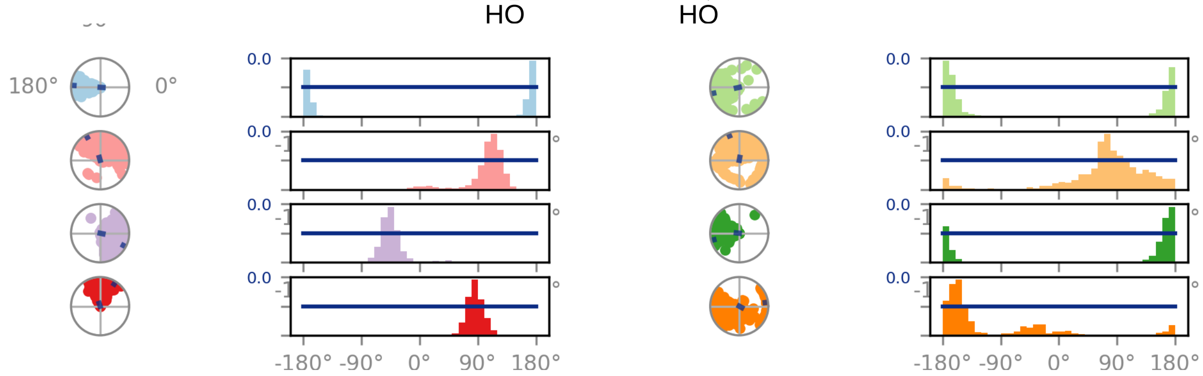

The ligand torsions plot summarizes the conformational evolution of every rotatable bond (RB) in the ligand throughout the simulation trajectory (0.00 through 100.00 nsec). The top panel shows the 2d schematic of a ligand with color-coded rotatable bonds. Each rotatable bond torsion is accompanied by a dial plot and bar plots of the same color.

Dial (or radial) plots describe the conformation of the torsion throughout the course of the simulation. The beginning of the simulation is in the center of the radial plot and the time evolution is plotted radially outwards.

The bar plots summarize the data on the dial plots, by showing the probability density of the torsion. If torsional potential information is available, the plot also shows the potential of the rotatable bond (by summing the potential of the related torsions). The values of the potential are on the left *Y*-axis of the chart, and are expressed in *kcal/mol*. Looking at the histogram and torsion potential relationships may give insights into the conformational strain the ligand undergoes to maintain a protein-bound conformation.

Ligand Properties

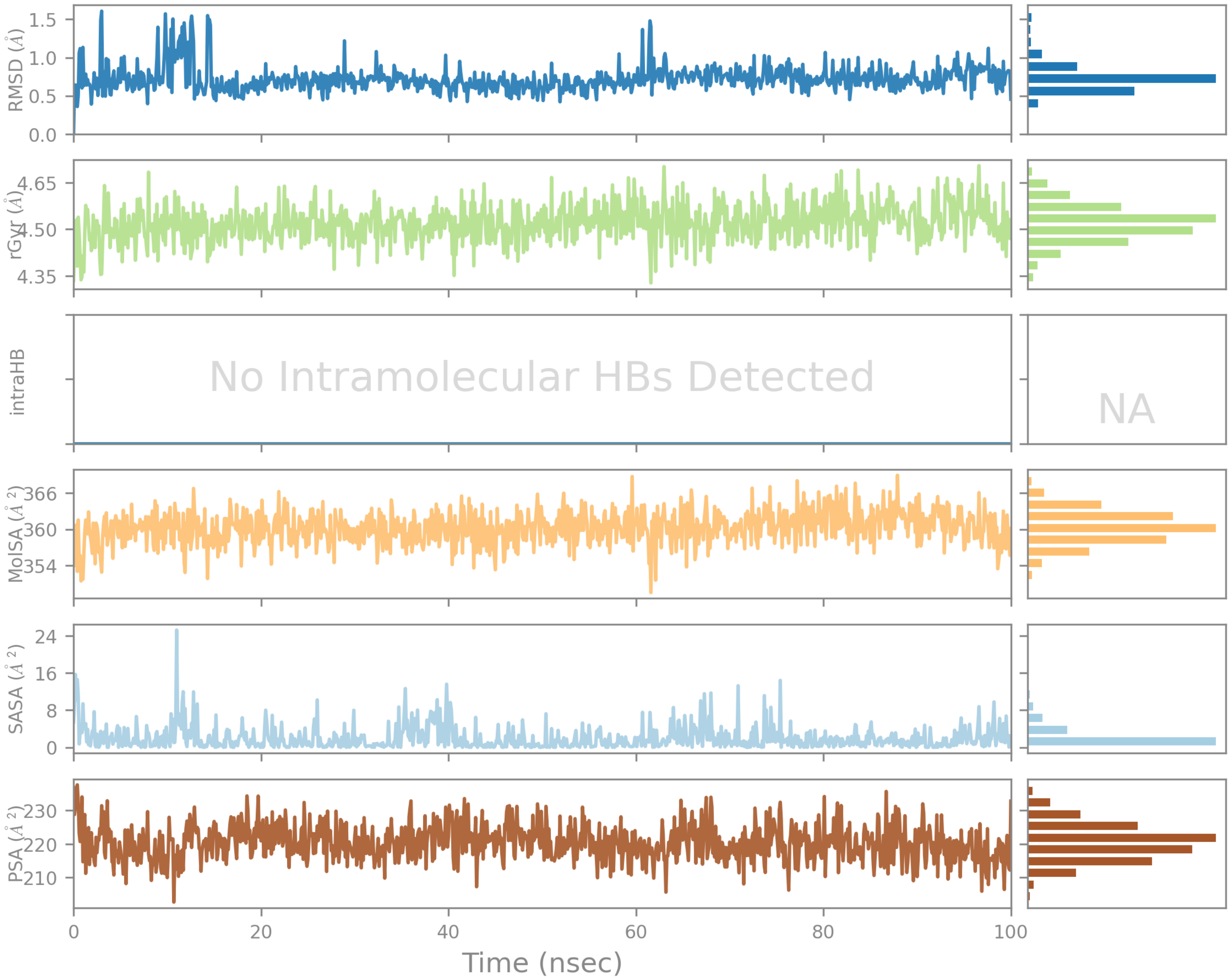

<u>Ligand RMSD</u>: Root mean square deviation of a ligand with respect to the reference conformation (typically the first frame is used as the reference and it is regarded as time *t*=0).

<u>Radius of Gyration (rGyr)</u>: Measures the 'extendedness' of a ligand, and is equivalent to its principal moment of inertia.

<u>Intramolecular Hydrogen Bonds (intraHB)</u>: Number of internal hydrogen bonds (HB) within a ligand molecule.

<u>Molecular Surface Area (MolSA)</u>: Molecular surface calculation with 1.4 Å probe radius. This value is equivalent to a van der Waals surface area.

<u>Solvent Accessible Surface Area (SASA)</u>: Surface area of a molecule accessible by a water molecule.

<u>Polar Surface Area (PSA)</u>: Solvent accessible surface area in a molecule contributed only by oxygen and nitrogen atoms.

Simulation Interactions Diagram Report

Simulation Details

Jobname: desmond_md_job_6KPF_Garcinisidone_E
Entry title: Garcinisidone E

CPU #	Job Type	Ensemble	Temp. [K]	Sim. Time [ns]	# Atoms	# Waters	Charge
1	mdsim	NPT	300.0	100.102	37832	11024	0

Protein Information

Tot. Residues	Prot. Chain(s)	Res. in Chain(s)	# Atoms	# Heavy Atoms	Charge
290	'R'	ict_values([290]	4620	2258	+13

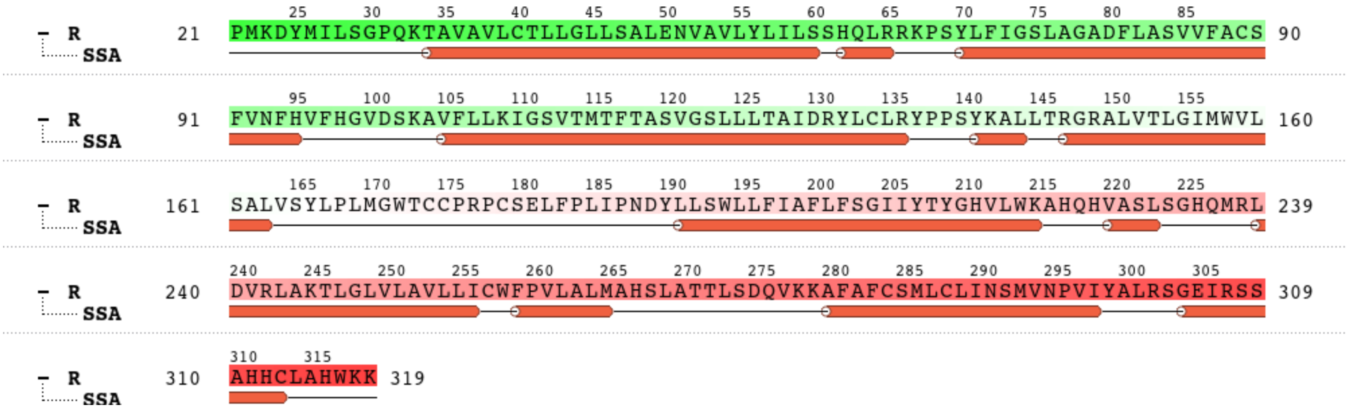

Ligand Information

SMILES	CC(C)=CCc(c1O)c(O)c(CC=C(C)C)c(O2)c1C(=O)Oc3c2cc(O)c(c34)OC(C)(C)C=C4
PDB Name	'UNK'
Num. of Atoms	65 (total) 35 (heavy)
Atomic Mass	478.547 au
Charge	0
Mol. Formula	C28H30O7
Num. of Fragments	3
Num. of Rot. Bonds	7

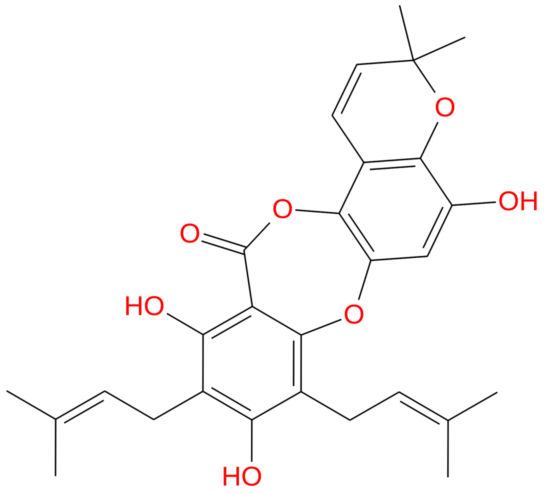

Counter Ion/Salt Information

Type	Num.	Concentration [mM]	Total Charge
Cl	44	72.569	-44
Na	31	51.128	+31

Protein-Ligand RMSD

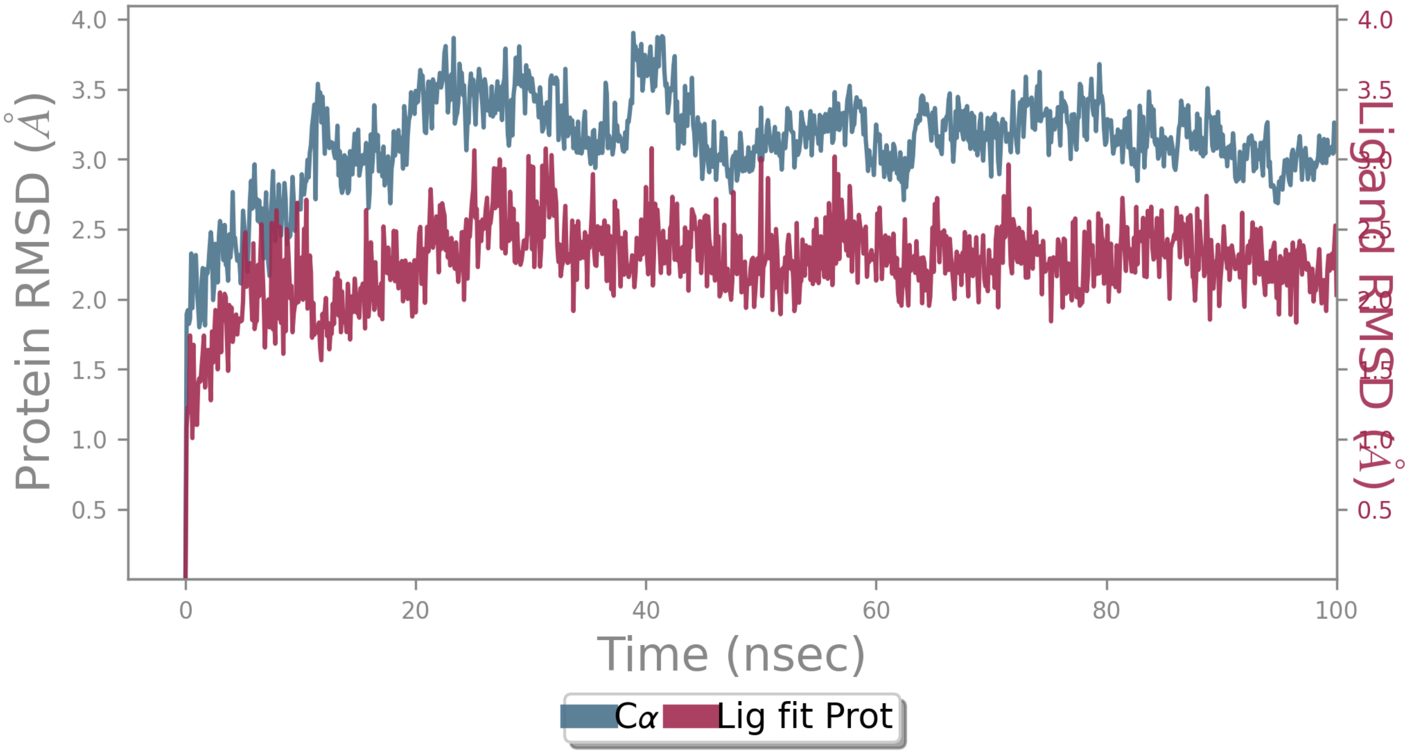

The Root Mean Square Deviation (RMSD) is used to measure the average change in displacement of a selection of atoms for a particular frame with respect to a reference frame. It is calculated for all frames in the trajectory. The RMSD for frame *x* is:

$$RMSD_x = \sqrt{\frac{1}{N}\sum_{i=1}^{N}(r'_i(t_x)) - r_i(t_{ref}))^2}$$

where *N* is the number of atoms in the atom selection; t_{ref} is the reference time, (typically the first frame is used as the reference and it is regarded as time *t*=0); and *r'* is the position of the selected atoms in frame *x* after superimposing on the reference frame, where frame *x* is recorded at time t_x. The procedure is repeated for every frame in the simulation trajectory.

Protein RMSD: The above plot shows the RMSD evolution of a protein (left *Y*-axis). All protein frames are first aligned on the reference frame backbone, and then the RMSD is calculated based on the atom selection. Monitoring the RMSD of the protein can give insights into its structural conformation throughout the simulation. RMSD analysis can indicate if the simulation has equilibrated — its fluctuations towards the end of the simulation are around some thermal average structure. Changes of the order of 1-3 Å are perfectly acceptable for small, globular proteins. Changes much larger than that, however, indicate that the protein is undergoing a large conformational change during the simulation. It is also important that your simulation converges — the RMSD values stabilize around a fixed value. If the RMSD of the protein is still increasing or decreasing on average at the end of the simulation, then your system has not equilibrated, and your simulation may not be long enough for rigorous analysis.

Ligand RMSD: Ligand RMSD (right *Y*-axis) indicates how stable the ligand is with respect to the protein and its binding pocket. In the above plot, 'Lig fit Prot' shows the RMSD of a ligand when the protein-ligand complex is first aligned on the protein backbone of the reference and then the RMSD of the ligand heavy atoms is measured. If the values observed are significantly larger than the RMSD of the protein, then it is likely that the ligand has diffused away from its initial binding site.

Protein RMSF

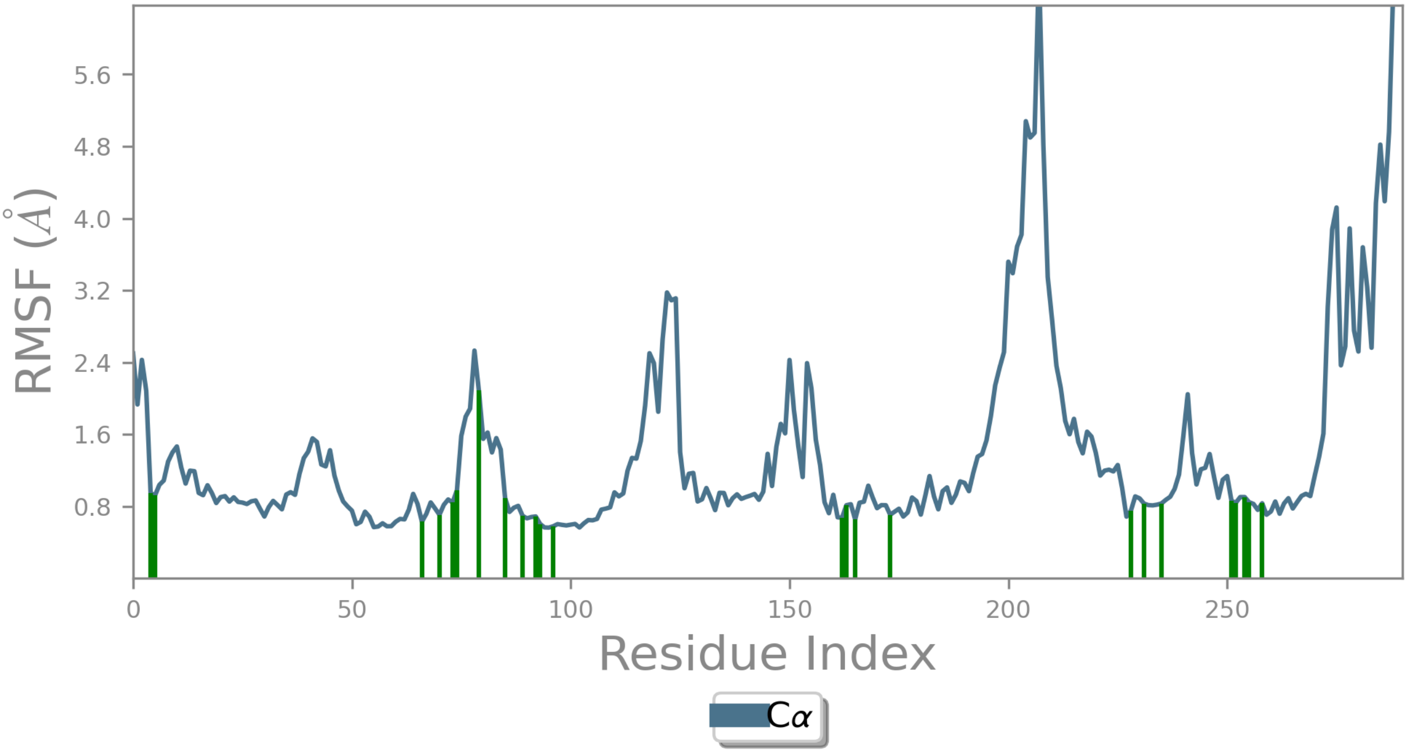

The Root Mean Square Fluctuation (RMSF) is useful for characterizing local changes along the protein chain. The RMSF for residue *i* is:

$$RMSF_i = \sqrt{\frac{1}{T}\sum_{t=1}^{T} < (r'_i(t)) - r_i(t_{ref}))^2 >}$$

where *T* is the trajectory time over which the RMSF is calculated, t_{ref} is the reference time, r_i is the position of residue *i*; *r'* is the position of atoms in residue *i* after superposition on the reference, and the angle brackets indicate that the average of the square distance is taken over the selection of atoms in the residue.

On this plot, peaks indicate areas of the protein that fluctuate the most during the simulation. Typically you will observe that the tails (*N*- and *C*-terminal) fluctuate more than any other part of the protein. Secondary structure elements like alpha helices and beta strands are usually more rigid than the unstructured part of the protein, and thus fluctuate less than the loop regions.

Ligand Contacts: Protein residues that interact with the ligand are marked with green-colored vertical bars.

Protein Secondary Structure

% Helix	% Strand	% Total SSE
69.32	0.00	69.32

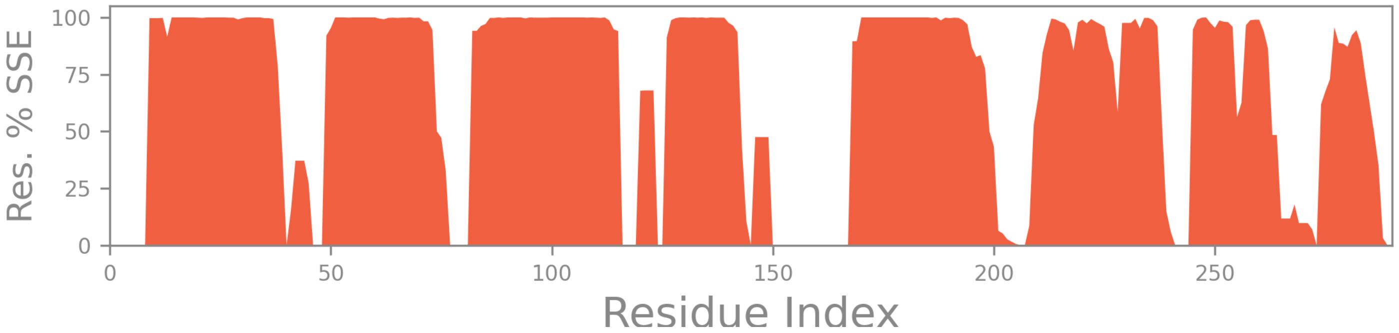

Protein secondary structure elements (SSE) like alpha-helices and beta-strands are monitored throughout the simulation. The plot above reports SSE distribution by residue index throughout the protein structure. The plot below summarizes the SSE composition for each trajectory frame over the course of the simulation, and the plot at the bottom monitors each residue and its SSE assignment over time.

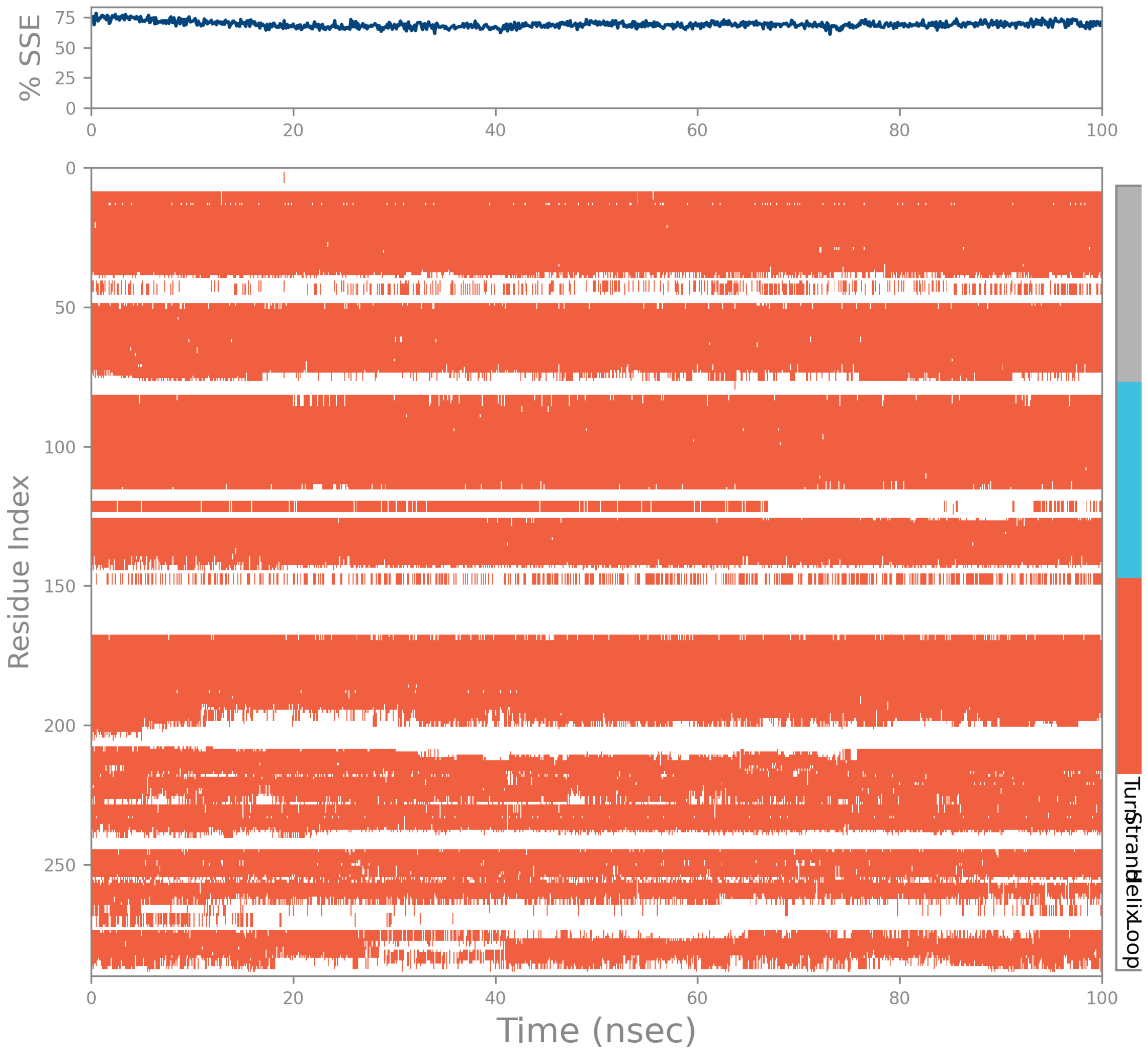

Ligand RMSF

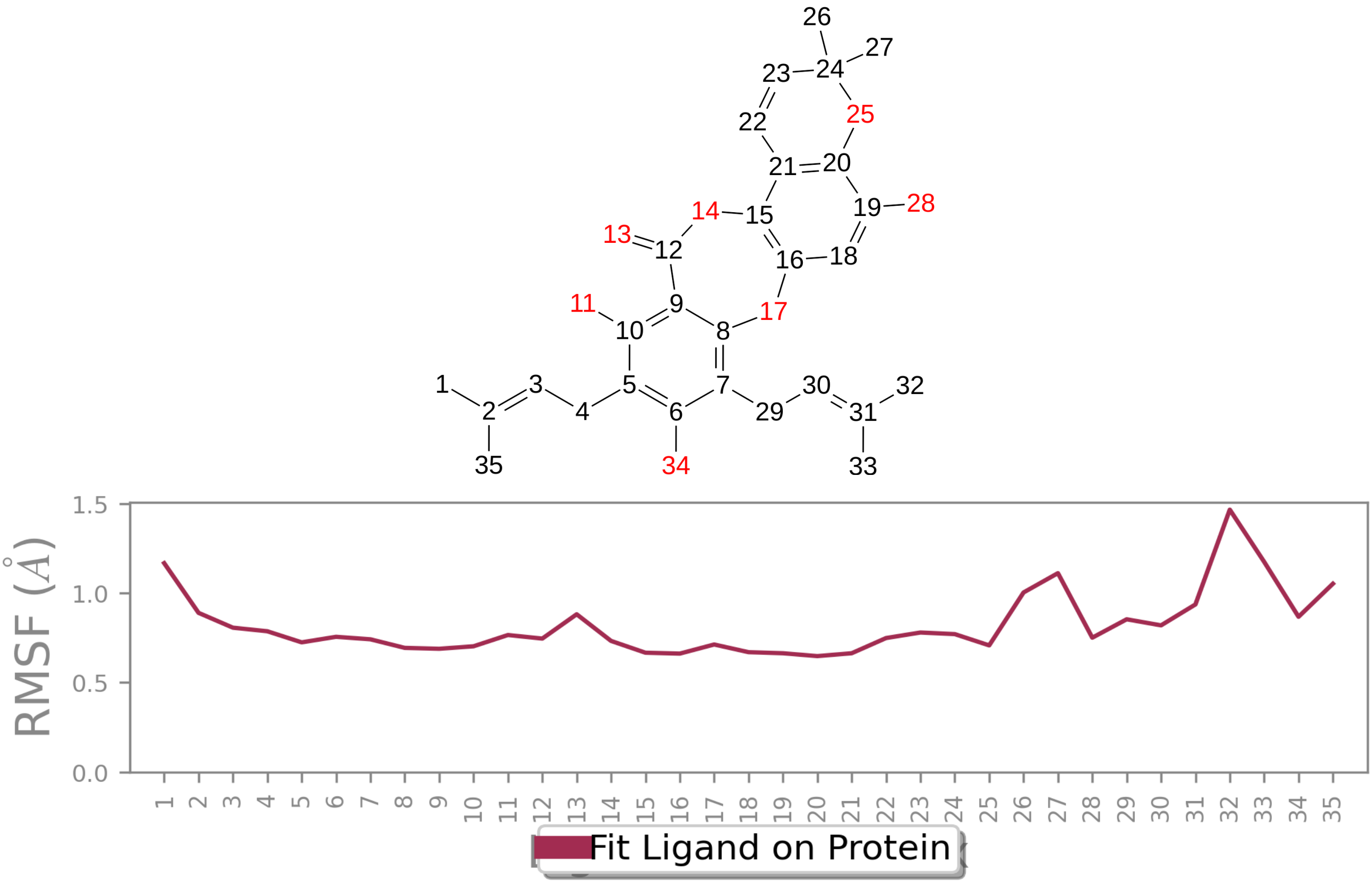

The Ligand Root Mean Square Fluctuation (L-RMSF) is useful for characterizing changes in the ligand atom positions. The RMSF for atom *i* is:

$$RMSF_i = \sqrt{\frac{1}{T}\sum_{t=1}^{T}(r'_i(t)) - r_i(t_{ref}))^2}$$

where T is the trajectory time over which the RMSF is calculated, t_{ref} is the reference time (usually for the first frame, and is regarded as the *zero* of time); r is the position of atom i in the reference at time t_{ref}, and r' is the position of atom i at time t after superposition on the reference frame.

Ligand RMSF shows the ligand's fluctuations broken down by atom, corresponding to the 2D structure in the top panel. The ligand RMSF may give you insights on how ligand fragments interact with the protein and their entropic role in the binding event. In the bottom panel, the 'Fit Ligand on Protein' line shows the ligand fluctuations, with respect to the protein. The protein-ligand complex is first aligned on the protein backbone and then the ligand RMSF is measured on the ligand heavy atoms.

Protein-Ligand Contacts

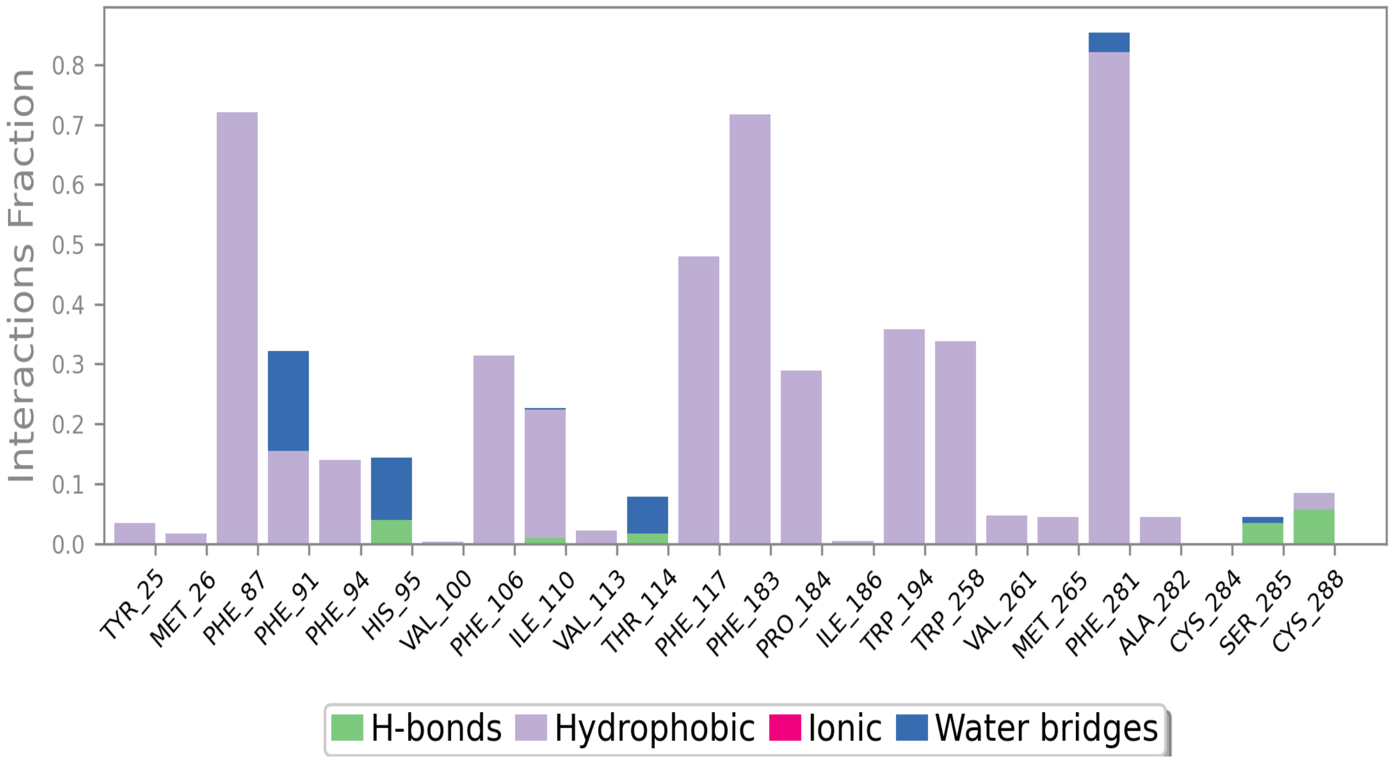

Protein interactions with the ligand can be monitored throughout the simulation. These interactions can be categorized by type and summarized, as shown in the plot above. Protein-ligand interactions (or 'contacts') are categorized into four types: Hydrogen Bonds, Hydrophobic, Ionic and Water Bridges. Each interaction type contains more specific subtypes, which can be explored through the 'Simulation Interactions Diagram' panel. The stacked bar charts are normalized over the course of the trajectory: for example, a value of 0.7 suggests that 70% of the simulation time the specific interaction is maintained. Values over 1.0 are possible as some protein residue may make multiple contacts of same subtype with the ligand.

Hydrogen Bonds: (H-bonds) play a significant role in ligand binding. Consideration of hydrogen-bonding properties in drug design is important because of their strong influence on drug specificity, metabolization and adsorption. Hydrogen bonds between a protein and a ligand can be further broken down into four subtypes: backbone acceptor; backbone donor; side-chain acceptor; side-chain donor.
The current geometric criteria for protein-ligand H-bond is: distance of 2.5 Å between the donor and acceptor atoms (D—H···A); a donor angle of ≥120° between the donor-hydrogen-acceptor atoms (D—H···A); and an acceptor angle of ≥90° between the hydrogen-acceptor-bonded_atom atoms (H···A—X).

Hydrophobic contacts: fall into three subtypes: π-Cation; π-π; and Other, non-specific interactions. Generally these type of interactions involve a hydrophobic amino acid and an aromatic or aliphatic group on the ligand, but we have extended this category to also include π-Cation interactions.
The current geometric criteria for hydrophobic interactions is as follows: π-Cation — Aromatic and charged groups within 4.5 Å; π-π — Two aromatic groups stacked face-to-face or face-to-edge; Other — A non-specific hydrophobic sidechain within 3.6 Å of a ligand's aromatic or aliphatic carbons.

Ionic interactions: or polar interactions, are between two oppositely charged atoms that are within 3.7 Å of each other and do not involve a hydrogen bond. We also monitor Protein-Metal-Ligand interactions, which are defined by a metal ion coordinated within 3.4 Å of protein's and ligand's heavy atoms (except carbon). All ionic interactions are broken down into two subtypes: those mediated by a protein backbone or side chains.

Water Bridges: are hydrogen-bonded protein-ligand interactions mediated by a water molecule. The hydrogen-bond geometry is slightly relaxed from the standard H-bond definition.
The current geometric criteria for a protein-water or water-ligand H-bond are: a distance of 2.8 Å between the donor and acceptor atoms (D—H···A); a donor angle of ≥110° between the donor-hydrogen-acceptor atoms (D—H···A); and an acceptor angle of ≥90° between the hydrogen-acceptor-bonded_atom atoms (H···A—X).

Protein-Ligand Contacts (cont.)

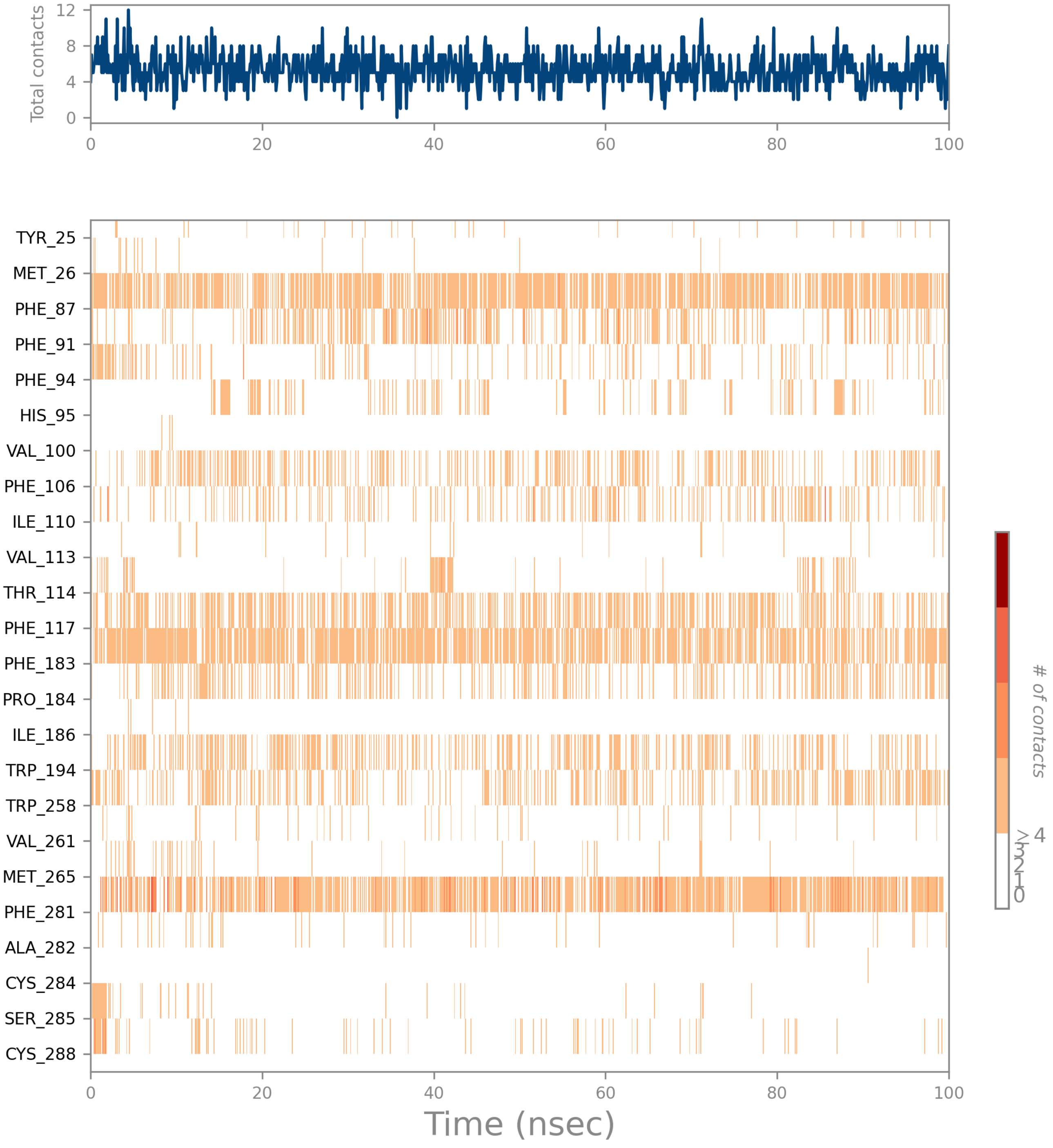

A timeline representation of the interactions and contacts (**H-bonds**, **Hydrophobic**, **Ionic**, **Water bridges**) summarized in the previous page. The top panel shows the total number of specific contacts the protein makes with the ligand over the course of the trajectory. The bottom panel shows which residues interact with the ligand in each trajectory frame. Some residues make more than one specific contact with the ligand, which is represented by a darker shade of orange, according to the scale to the right of the plot.

Schrödinger

Ligand-Protein Contacts

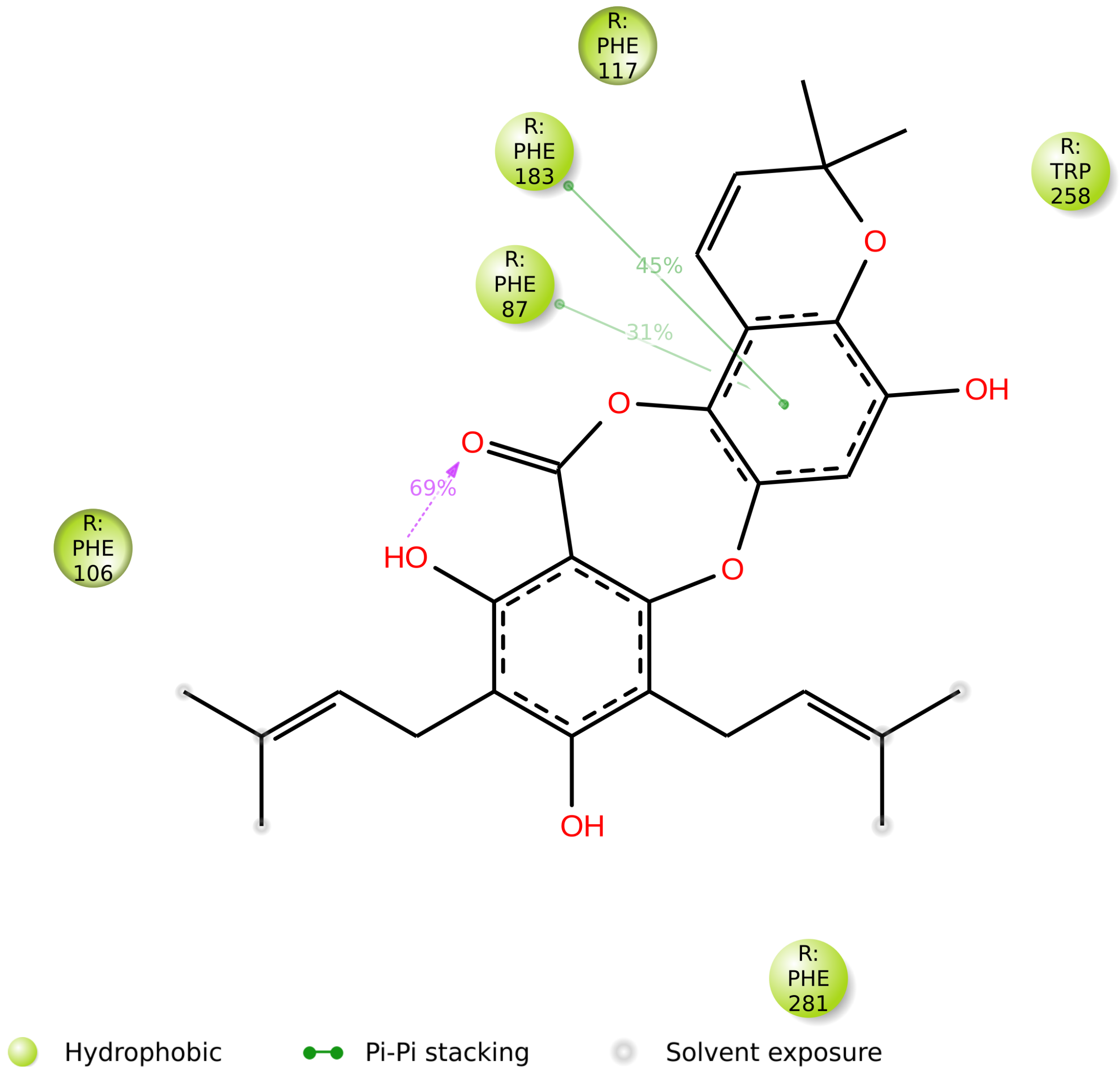

A schematic of detailed ligand atom interactions with the protein residues. Interactions that occur more than **30.0%** of the simulation time in the selected trajectory (0.00 through 100.00 nsec), are shown.
Note: it is possible to have interactions with >100% as some residues may have multiple interactions of a single type with the same ligand atom. For example, the ARG side chain has four H-bond donors that can all hydrogen-bond to a single H-bond acceptor.

Ligand Torsion Profile

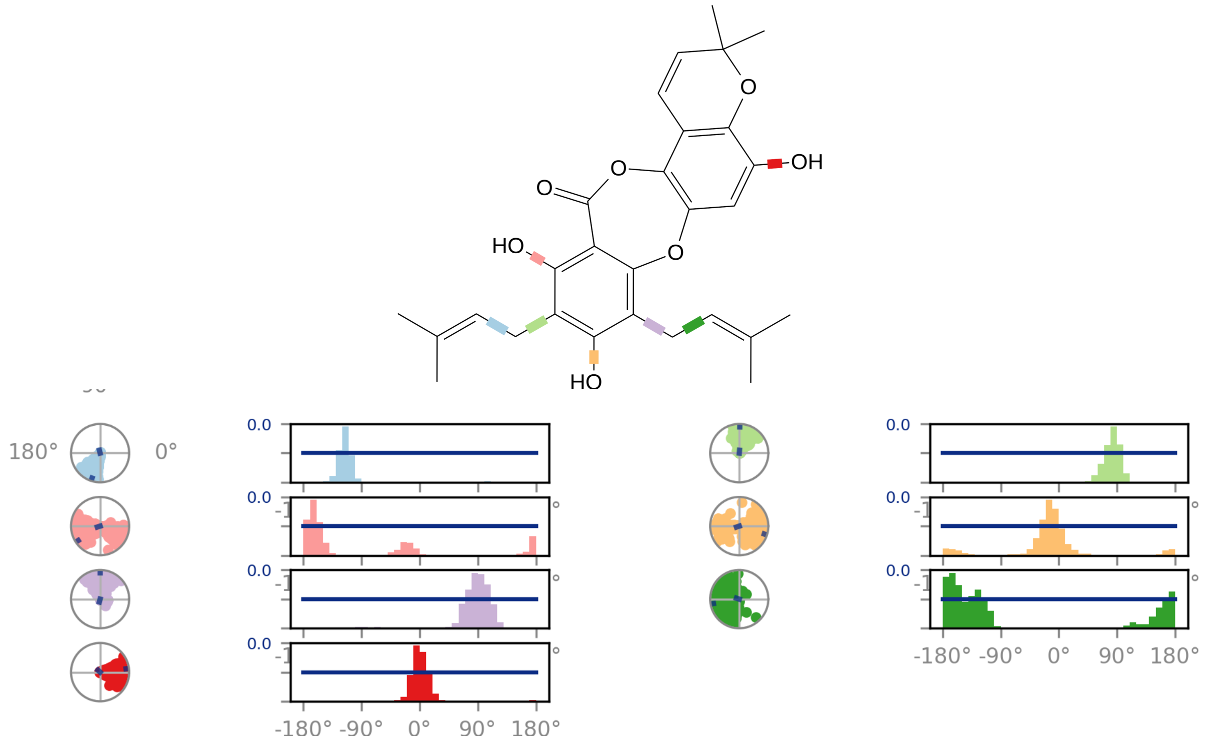

The ligand torsions plot summarizes the conformational evolution of every rotatable bond (RB) in the ligand throughout the simulation trajectory (0.00 through 100.00 nsec). The top panel shows the 2d schematic of a ligand with color-coded rotatable bonds. Each rotatable bond torsion is accompanied by a dial plot and bar plots of the same color.

Dial (or radial) plots describe the conformation of the torsion throughout the course of the simulation. The beginning of the simulation is in the center of the radial plot and the time evolution is plotted radially outwards.

The bar plots summarize the data on the dial plots, by showing the probability density of the torsion. If torsional potential information is available, the plot also shows the potential of the rotatable bond (by summing the potential of the related torsions). The values of the potential are on the left *Y*-axis of the chart, and are expressed in *kcal/mol*. Looking at the histogram and torsion potential relationships may give insights into the conformational strain the ligand undergoes to maintain a protein-bound conformation.

Ligand Properties

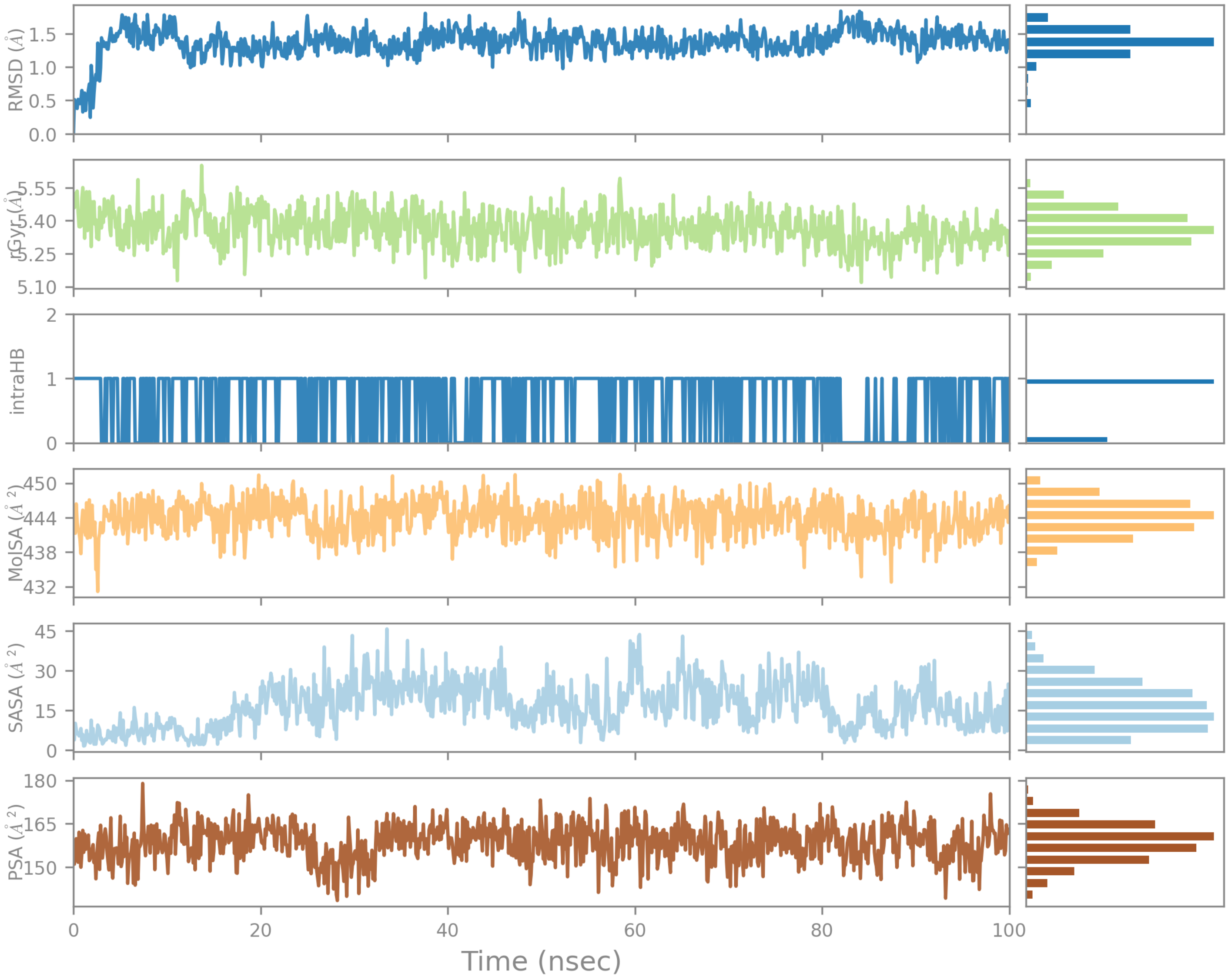

Ligand RMSD: Root mean square deviation of a ligand with respect to the reference conformation (typically the first frame is used as the reference and it is regarded as time *t*=0).

Radius of Gyration (rGyr): Measures the 'extendedness' of a ligand, and is equivalent to its principal moment of inertia.

Intramolecular Hydrogen Bonds (intraHB): Number of internal hydrogen bonds (HB) within a ligand molecule.

Molecular Surface Area (MolSA): Molecular surface calculation with 1.4 Å probe radius. This value is equivalent to a van der Waals surface area.

Solvent Accessible Surface Area (SASA): Surface area of a molecule accessible by a water molecule.

Polar Surface Area (PSA): Solvent accessible surface area in a molecule contributed only by oxygen and nitrogen atoms.
